# Supplementary material for: Impact of employing primary healthcare professionals in emergency department triage on patient flow outcomes: a systematic review and meta-analysis
Source: BMJ Open. 2022 Apr 19;12(4):e052850. doi: 10.1136/bmjopen-2021-052850 (PMC9058787; doi:10.1136/bmjopen-2021-052850)
Supplement: Supplementary data [file bmjopen-2021-052850supp001.pdf]

Appendix Methods:

The National Institute for Health and Care Excellence (NICE) quality appraisal tool has four domains: characteristics of the population, allocation methods, outcomes, and analyses. Each domain has multiple questions for which there are five response options: (1) study has been conducted in such a way to minimize the risk of bias (++), (2) study has not addressed all potential sources of bias (+), (3) significant sources of bias persists in the study (-), (4) not reported or (5) not applicable. A fifth domain summarizes the overall quality of the included study based on the assessments of the four domains. The overall quality of each included study was assessed as either low quality (-), moderate quality (+) or high quality (++), based on adjudications made on the four individual domains for that study.

Appendix Results:

1. ***Patient triage acuity rating***: Various scales such as Emergency Severity Index (ESI), Australasian Triage Scale (ATS), the Canadian Triage or Acuity Scale (CTAS) or triage acuity rating scale were used to assess the acuity levels patients arriving at the ED. Six included studies[1-6] (15%) reported triaging patients who were triage category 3-5 (one study involved <10% of category 3 patients, two studies[2,7] involved >20% of category 3 patients, and three studies[1,4,5] did not report the percentage of patients in each category 3-5). Five included studies[8-12] (10%), reported triaging both low and high acuity patients (with approximately 90% of category 3-5 patients). Fourteen (35%) studies[13-26] did not report acuity levels of patients.

2. ***Time to physician initial assessment (PIA) sub-grouped by various PHCP interventions:***

   Of the 14 studies, the majority[18,26,32,42,48,61,63,66,67] (n = 9) reported the effect of NP team triage on PIA, and the rest reported either the effect of GP team triage[54,69] (n = 2) or nurse

triage-plus[39,51,60] (n=3) on PIA, respectively. All studies in NP team triage group showed a decrease in PIA (median [range]= -21.7 minutes [-2.3 to -50]) favoring the intervention group. In the nurse triage-plus group all except one[51] showed a decrease in PIA (median [range]= -2.4 minutes [-2 to -31]), Among the two studies[54,69] in GP team triage group, one prospective CBA interventional study[54] reported statistically significant decrease (-18 minutes) in PIA favoring the intervention group. Whereas the second cross-sectional observational study[69] showed an increase (4.43 minutes) in PIA (reported as statistically significant), favoring the traditional nurse-led triage model.

3. ***Emergency department length of stay (ED LOS) sub-grouped by various PHCP interventions:***

Twenty studies[7,8,13-19,21-31] reported on the effect of nurse triage-plus on ED LOS, nine studies[1,2,4-6,10,32-34] reported on the effect of NP team triage on ED LOS, and one study[11] on the effect of GP team triage on ED LOS. Seventeen studies[7,8,13,15-19,21,23-25,27-31] in the nurse triage-plus model reported a decrease (median = -18 minutes) in ED LOS favoring the intervention group. All nine studies[1,2,4-6,10,32-34] in the NP team triage model showed a decrease (median = -28.50 minutes) in ED LOS favoring the intervention group. One study in the GP team triage model did not show any significant difference in ED LOS between comparison groups.

Four studies reported percentage of patients discharged within benchmark times (ED specific). Rogers et al. reported 41% of patients discharged from the ED within one hour in the NP team triage group compared to only 16% patients discharged within one hour in the traditional nurse-led triage group. Tsai et al.[35] reported that 30% of low-acuity patients in the NP team triage group discharged in 90 minutes compared to 12% in the

traditional nurse-led triage group. Day et al.[1] reported that 85.7% of patients discharged under 6 hours in the NP team triage group compared to 80.1% in the traditional nurse-led triage group. Uthman et al.[36] reported that 98.1% of patients discharged under 4 hours in the in the NP team triage group compared to 94.7% in the traditional nurse-led triage group.

4. ***Effect of PHCP intervention on number of repeat ED visits***

Zager et al.6 reported a 5% decrease in ED visits in the NP team triage group (conducted triage, medical screening exam (MSE) and discharged low-acuity patients with a same day appointment at the GP clinic co-located with the ED) compared to the traditional nurse-led triage model (statistical significance not reported). Day et al.1 investigating NP team triage (provider at triage model) reported 2194 ED visits (over 6 weeks) during pre-intervention period compared to 1699 patient visits (over one month) during the post-intervention period (statistical significance not reported). Tucker et al.34 investigated the effect of NP team triage on ED visits and reported an increase in the number of patients visiting ED by 51 visits per month (statistical significance not reported) compared to the traditional nurse-led triage model. Bersselaar et al.37 investigated the effect of GP team triage and x-ray requests (at the emergency care access point (ECAP) in which ED and GP work together) on ED visits, and reported that 68% of patient visits were treated by the GP without ED referral leading to a reduction of 4.5% annual ED patient visits. Kool et al.38, a CBA study, investigated the effect of GP team triage at the integrated emergency post (IEP) with a joint reception for the ED and a GP clinic on ED visits compared to the control sites that are not IEP (traditional nurse-led triage model), and reported a statistically significant decrease (6257 to 5715) in the number of patient visits

at the ED at IEPs and an statistically significant increase (3985 to 4321) in the number of ED attendances at the control sites. Gaucher et al20 reported that number of return ED visits decreased from 8.1% to 6.1% in the nurse triage-plus group compared to the traditional nurse-led triage model.

5. ***Effect of PHCP intervention on patient satisfaction***

Kool et al.[37] reported no differences in patient satisfaction between patients who visited IEPs (GP team triage) compared to those who visited ED's at control sites, but patients who were phone triaged at the IEP were more satisfied (statistically significant) compared to the control sites EDs[37]. Tucker et al.[34] investigated the effect of NP team triage on ED visits and reported that patient satisfaction remained high (greater than 90%; statistical significance not reported) compared to the traditional nurse-led triage model. Gardner et al.[32] reported that with NP team triage, 62-65% of patients were more satisfied with their ED LOS, PIA and quality of care compared to traditional nurse-led triage model. Hayden et al.[2] investigated the impact of NP team triage (provider at triage model) on patient satisfaction and reported that patient satisfaction decreased slightly in the post-intervention period compared to the pre-intervention period but this decrease was not statistically significant. Five[7,12,15,16,25] studies reported an increase in patient satisfaction scores in the nurse triage-plus model compared to the traditional nurse-led triage model, whereas one[18] study reported no difference between groups.

6. ***Effect of PHCP intervention on time to triage***

One RCT[28] showed a non-significant decrease in time to triage in the nurse triage-plus group compared to traditional nurse-led triage group. Two pre-post studies[33,38] reported the effect of NP team triage on time to triage compared to the traditional nurse-led triage

model. MacKenzie et al.[38] reported statistically significant decrease (pre-intervention time to triage (Median: 4; IQR: (2, 10)); post-intervention time to triage (Median: 3; IQR: (1, 8)) favoring the intervention. Rogers et al.[33] reported that 98% percentage of patients in the NP team triage intervention group were triaged within 15 minutes compared to the comparison group (75% of patients triaged within 15 minutes).

Appendix Table 1: Inclusion and exclusion criteria

	**Inclusion criteria**	**Exclusion criteria**
Population	Patient population (children and adults of any age) visiting the ED	
Intervention	Any ED triage intervention or strategy involving primary healthcare providers (family physicians/general practitioner (GP), nurse practitioner (NP), or nurse given increased authority)	Studies reporting triage intervention involving emergency physicians (ED MD) or exclusively physician assistants
Comparator	Traditional nurse-led triage (standard care)	
Outcomes	*Primary outcomes*: Time to provider initial assessment	
	Secondary outcomes: ED LOS, proportion of patients that left without being seen (LWBS), ED length of stay patient satisfaction, proportion of patients leaving against medical advice (LAMA), time to triage, and number of ED visits.	
Study Design	Any comparative study design (randomized and quasi-randomized clinical trials, non-randomized controlled clinical trial/controlled before and after studies (CBA), case control studies, controlled cohort studies, interrupted time series, pre-post intervention/uncontrolled before and after studies)	Reviews, commentary, case reports, editorials, historical articles, non-human studies

Appendix Table 2: Medline search strategy

Database: Ovid MEDLINE(R) and Epub Ahead of Print, In-Process & Other Non-Indexed Citations and Daily <1946 to January 10, 2020>
Search Strategy:
----

1 exp primary health care/ (140156)
2 physicians, family/ (15853)
3 family practice/ (64029)
4 Physicians, Primary Care/ (2703)
5 general practice/ (11719)
6 general practitioners/ (6381)
7 (primary adj2 (care or health*)).ti,ab,kf. (129329)
8 ((general or family) adj (practice* or practitioner*)).ti,ab,kf. (84810)
9 (GP or GPs).ti,ab,kf. (51935)
10 nurse practitioners/ (16770)
11 primary care nursing/ (392)
12 family nursing/ (1349)
13 community mental health services/ (17905)
14 ((family or community or primary or ambulatory or triage) adj2 (medic* or doctor* or physician* or health* or nurs*)).ti,ab,kf. (68438)
15 Ambulatory Care/ (40524)
16 (ambulatory adj2 care).ti,ab,kf. (11413)
17 Health Services, Indigenous/ (2817)
18 Cultural Competency/ (4632)
19 Culturally Competent Care/ (830)
20 Medicine, Traditional/ (10299)
21 (trauma adj inform*).ti,ab,kf. (617)
22 (aborigin* or indigenous or native).ti,ab,kf. (225451)
23 ((after or out) adj2 hour*).ti,ab,kf. (137459)
24 or/1-23 (917850)
25 exp Emergency Service, Hospital/ (67418)
26 Emergency Medical Services/ (39232)
27 emergency treatment/ (10025)
28 Trauma centers/ (9210)
29 Triage/ (10240)
30 ((emergency or emergent or urgent) adj2 (care or healthcare or department* or unit or units or room* or treatment* or ward or service)).ti,ab,kf. (121497)
31 ("accident and emergency" or "accident & emergency" or ED or EDs or ER or A&E).ti,ab,kf. (162648)
32 (triage adj2 (centre or centres or center or centers or department? or unit or units)).ti,ab,kf. (538)
33 (emergency adj2 (care or healthcare or department? or unit or units or room? or treatment? or care or visit? or utilization or admit or admission?)).ti,ab,kf. (112731)
34 ("accident and emergency" or "accident & emergency" or emergency service?).ti,ab,kf. (10865)
35 (trauma adj2 (centre or centres or center or centers or department? or unit or units)).ti,ab,kf. (15573)
36 (triage adj2 (centre or centres or center or centers or department? or unit or units)).ti,ab,kf. (538)

37 (emergency adj2 (visit? or care or admit or admission?)).ti,ab,kf. (26760)
38 (urgent adj2 (care or healthcare or health care)).ti,ab,kf. (2099)
39 ((semiurgent or semi-urgent or nonemergen$ or non-emergen$) adj2 (treatment? or care or visit?)).ti,ab,kf. (289)
40 ((emergency or non-emergency or nonemergency or urgent or non-urgent or nonurgent or semi-urgent or semiurgent) adj2 patient?).ti,ab,kf. (11636)
41 or/25-40 (367776)
42 organizational efficiency/ (20744)
43 workflow/ (3295)
44 Waiting lists/ (10724)
45 ((wait or waiting) adj2 (time or times or list or lists)).ti. (3351)
46 ((wait or waiting or throughput or service or treatment) adj2 (time or times or list or lists) adj10 (reduce? or reduction or eliminat$ or lower or fewer or intervention or policy or policies or reform$ or effectiveness or impact or improv$ or organi?ational$ or quality or save or saving)).ab. (3119)
47 ((decrease or reduce or streamline or less or minimize or shorten or eliminate or cut or enhance or facilitate or speed or better or accelerate or optimize or reform or delay or change or faster or impact$ or assess$ or eliminat$ or improv$ or lower$ or reduc$) adj3 patient? wait$).ti,ab,kf. (303)
48 CROWDING/ (2930)
49 crowd$.ti,ab,kf. (16513)
50 congest$.ti,ab,kf. (61747)
51 overcrowd$.ti,ab,kf. (3425)
52 gridlock$.ti,ab,kf. (180)
53 queue$.ti,ab,kf. (1011)
54 overload$.ti,ab. (39413)
55 "access block$".ti,ab,kf. (166)
56 (throughput or through-put).ti,ab,kf. (87262)
57 warehous$.ti,ab,kf. (2303)
58 ("left without being seen" or "leave$ without being seen" or lwbs).ti,ab,kf. (284)
59 (patient adj2 elop$).ti,ab,kf. (16)
60 (ambulance$ adj2 diver$).ti,ab,kf. (194)
61 (ambulance$ adj2 redirect$).ti,ab,kf. (3)
62 "fast track$".ti,ab,kf. (3500)
63 delay$.ti,ab,kf. (428757)
64 ("patient flow$" or "flow of patient$").ti,ab,kf. (4939)
65 defer$.ti,ab,kf. (23198)
66 (over* adj3 (capacit$ or occupanc$)).ti,ab,kf. (4603)
67 (lama or (leave$ adj4 ("medical advice" or treatment$)) or (left adj4 ("medical advice" or treatment$))).ti,ab,kf. (8393)
68 ((hallway or corridor) adj2 (care or medicine)).ti,ab,kf. (6)
69 or/42-68 (776721)
70 24 and 41 and 69 (3799)

Appendix Table 3: Grey literature sources

**Grey literature sources**
BMJ Open Quality (https://bmjopenquality.bmj.com) and a Google Custom Search of the following websites:<br>Canadian Foundation for Healthcare Improvement (www.cfhi-fcass.ca), Institute for Healthcare Improvement (www.ihi.org), Agency for Healthcare Research and Quality (www.ahrq.gov), NHS Improvement (https://improvement.nhs.uk), International Society for Quality in Health Care (www.isqua.org), Health Quality Ontario (www.hqontario.ca), Saskatchewan Health Quality Council (https://hqc.sk.ca), Health Quality Council of Alberta (www.hqca.ca), BC Patient Safety & Quality Council (https://bcpsqc.ca), Australian Commission on Safety and Quality in Health Care (www.safetyandquality.gov.au), and Health Quality & Safety Commission New Zealand (www.hqsc.govt.nz).

Appendix Table 4: Quality assessment scores of included studies

**Study ID**	**1.1**	**1.2**	**1.3**	**2.1**	**2.2**	**2.3**	**2.4**	**2.5**	**2.6**	**2.7**	**2.8**	**2.9**	**3.1**	**3.2**	**3.3**	**3.4**	**3.5**	**3.6**	**4.1**	**4.2**	**4.3**	**4.4**	**4.5**	**4.6**	**5.1**	**5.2**
Celona, 2018	2+	2+	-	-	2+	-	-	2+	2+	NR	-	2+	2+	2+	2+	2+	2+	2+	NR	NR	NR	+	+	-	-	-
Cheung, 2002	2+	2+	NR	-	2+	-	NR	2+	NR	NR	-	-	2+	+	2+	2+	2+	2+	NR	NR	NR	+	+	-	-	-
Day, 2013	2+	2+	-	-	2+	-	-	2+	2+	NR	-	2+	2+	2+	2+	2+	2+	2+	NR	NR	NR	2+	+	2+	-	-
Edwards, 2011	2+	2+	2+	-	2+	-	-	+	2+	NR	-	2+	2+	2+	2+	2+	2+	2+	NR	NR	NR	2+	+	-	-	-
Gardner, 2018	+	2+	+	-	2+	-	-	2+	2+	NR	-	2+	2+	2+	2+	2+	2+	2+	NR	NR	NR	2+	+	2+	-	+
Hayden, 2014	+	+	+	-	2+	-	-	2+	2+	NR	-	2+	2+	2+	2+	2+	2+	2+	+	NR	NR	2+	2+	+	-	+
Lee, 2016	2+	+	2+	2+	2+	2+	+	2+	2+	NR	2+	2+	+	+	2+	2+	2+	2+	+	NR	2+	2+	2+	2+	+	+
Lindley Jones, 2000	2+	2+	2+	2+	2+	+	NR	2+	2+	NR	2+	+	2+	2+	2+	2+	2+	2+	NR	NR	NR	+	+	2+	+	2+
Love, 2012	2+	+	+	-	2+	-	-	2+	2+	NR	-	2+	2+	2+	2+	2+	2+	2+	NR	NR	NR	+	+	-	-	+
Mackenzie, 2015	2+	2+	2+	-	2+	-	-	2+	2+	NR	-	2+	2+	2+	2+	2+	2+	2+	-	NR	NR	2+	+	+	-	2+
Parris, 1997	2+	2+	-	-	2+	-	-	2+	2+	NR	-	2+	+	2+	2+	2+	2+	2+	NR	NR	NR	+	+	2+	-	-
Pierce, 2016	2+	+	-	-	2+	NR	-	2+	2+	NR	-	2+	2+	2+	2+	2+	2+	2+	NR	NR	NR	+	NR	NR	-	-
Rogers, 2004	2+	+	NR	-	2+	-	-	2+	2+	NR	-	2+	2+	2+	2+	2+	2+	2+	NR	NR	NR	+	-	-	-	+
Shrimpling, 2002	2+	2+	+	-	2+	-	-	2+	2+	NR	-	2+	+	2+	2+	2+	2+	2+	NR	NR	NR	+	NR	-	-	+
Thurston, 1996	2+	+	2+	+	2+	NR	NR	+	2+	NR	+	2+	+	+	2+	2+	2+	2+	NR	NR	2+	2+	+	2+	+	+

Tsai, 2012	2+	+	+	-	2+	-	-	2+	2+	NR	-	2+	2+	2+	2+	2+	2+	2+	NR	NR	NR	2+	2+	2+	-	+
Tucker, 2015	2+	+	+	-	2+	-	-	2+	2+	NR	-	2+	2+	2+	2+	2+	2+	2+	NR	NR	NR	+	NR	-	-	+
Uthman, 2018	2+	2+	+	-	2+	NR	NR	2+	2+	NR	+	2+	2+	2+	2+	2+	2+	2+	2+	NR	NR	2+	2+	2+	-	+
van den Bersselaar, 2018	2+	2+	+	-	2+	-	NR	2+	2+	NR	-	2+	2+	2+	2+	2+	2+	2+	NR	NR	NR	+	+	-	-	+
van Gils-van Rooij, 2018	+	2+	2+	-	2+	-	NR	2+	2+	NR	-	2+	2+	2+	2+	2+	2+	2+	+	NR	NR	+	2+	+	-	+
Zager, 2018	2+	2+	+	-	2+	-	-	2+	2+	NR	-	2+	2+	2+	2+	2+	2+	2+	NR	NR	NR	+	NR	+	-	+
Kool, 2008	2+	+	-	-	2+	-	-	2+	2+	NR	-	2+	+	-	2+	2+	2+	2+	+	NR	NR	2+	2+	+	-	-
Al Abri, 2020	2+	2+	+	-	2+	-	-	2+	NR	NR	-	2+	2+	2+	2+	2+	2+	2+	+	NR	NR	-	2+	2+	+	+
Ho, 2018	2+	2+	+	+	2+	NR	-	2+	2+	NR	2+	2+	2+	2+	2+	2+	2+	2+	2+	2+	2+	2+	2+	2+	-	+
Li, 2018	+	2+	+	-	2+	-	-	2+	NR	NR	-	2+	2+	2+	2+	2+	2+	2+	-	NR	NR	-	2+	+	-	+
Hackman, 2015	+	+	+	-	2+	-	-	2+	NR	NR	-	2+	2+	2+	2+	2+	2+	2+	-	NR	NR	2+	2+	2+	-	+
Klassen, 1993	2+	2+	+	2+	2+	+	+	2+	NR	NR	2+	2+	2+	2+	2+	2+	2+	2+	2+	-	NR	-	2+	+	-	+
Al Khadi, 2017	+	+	+	-	2+	-	-	2+	NR	NR	-	2+	2+	2+	2+	2+	2+	2+	-	NR	NR	-	2+	+	-	+
Ashurst, 2014	+	2+	+	-	2+	-	-	2+	NR	NR	-	2+	+	2+	2+	2+	2+	2+	-	2+	2+	-	2+	+	-	+
Fan, 2006	+	2+	+	2+	2+	+	+	2+	2+	NR	2+	+	+	+	2+	2+	2+	2+	2+	2+	2+	2+	2+	2+	+	+
Sikkenga, 2016	+	+	+	-	2+	-	-	2+	2+	NR	-	2+	2+	2+	2+	2+	2+	2+	-	NR	NR	NR	2+	2+	-	+
Lee, 1996	+	+	+	-	2+	-	-	2+	2+	NR	-	2+	2+	2+	2+	2+	2+	2+	-	NR	NR	-	2+	2+	-	+

Dixon, 2014	+	2+	+	2+	2+	+	-	2+	2+	NR	2+	2+	2+	2+	2+	2+	2+	2+	2+	-	2+	NR	2+	2+	-	+
Lee, 2014	+	2+	+	+	2+	NR	+	2+	2+	NR	2+	2+	+	2+	2+	2+	2+	2+	2+	NR	2+	+	2+	+	+	+
Adam, 2014	+	2+	+	+	2+	2+	+	2+	2+	NR	2+	2+	2+	2+	2+	2+	2+	2+	2+	NR	2+	+	2+	2+	+	+
Fontanel, 2011	+	2+	+	-	2+	-	-	2+	2+	NR	-	NR	2+	2+	2+	2+	2+	2+	+	NR	NR	NR	NR	2+	-	+
Gaucher, 2010	+	2+	+	-	2+	-	-	2+	2+	NR	-	2+	2+	2+	2+	2+	2+	2+	-	NR	2+	2+	2+	2+	-	+
Demarco, 2010	+	2+	+	-	2+	-	-	2+	2+	NR	-	2+	2+	2+	2+	2+	2+	2+	-	NR	NR	-	2+	+	-	+
Jobe, 2013	+	2+	+	+	2+	NR	NR	2+	NR	NR	2+	2+	2+	+	2+	2+	2+	2+	2+	2+	NR	-	-	-	-	+
Lijuan, 2017	2+	2+	+	-	2+	-	-	2+	2+	NR	-	2+	+	2+	2+	2+	2+	2+	-	NR	NR	-	2+	+	-	+

Appendix Figure 1: Effectiveness of PHCP interventions on time to provide initial assessment (in minutes) sub-grouped by interventions.

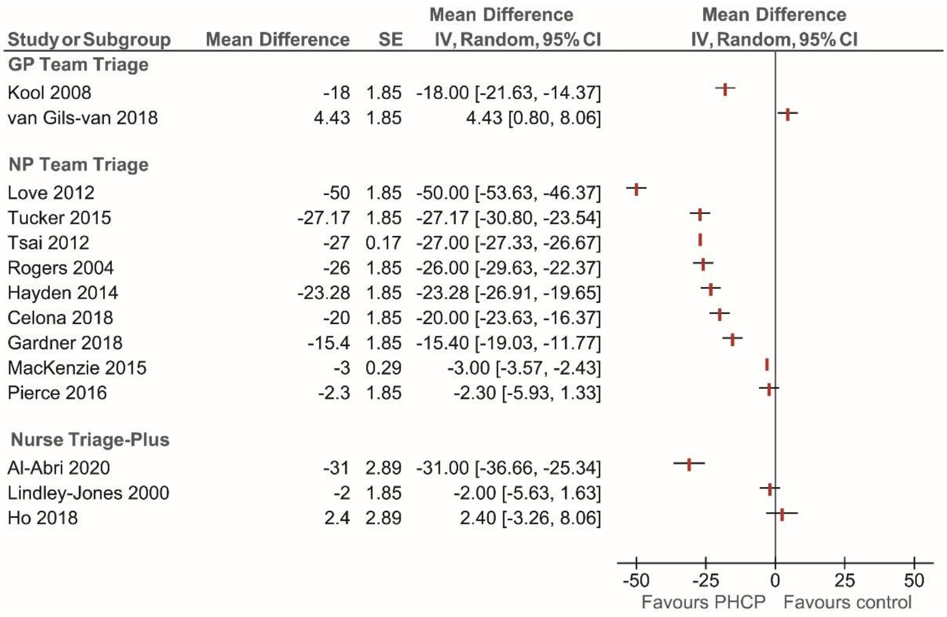

The horizontal black lines represent 95% confidence intervals and the red dots in the middle represents point estimates (mean difference).

Appendix Figure 2: Effectiveness of PHCP interventions on achieving benchmark time to provider initial assessment

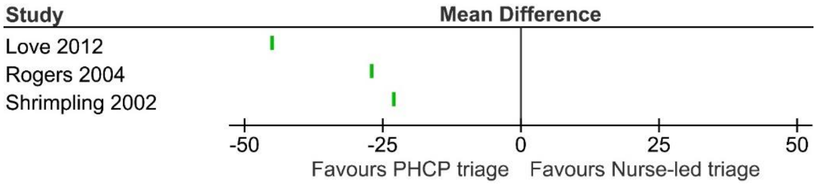

The horizontal black lines represent 95% confidence intervals and the red dots in the middle represents point estimates (mean difference).

Appendix Figure 3: Effectiveness of PHCP interventions on ED LOS (in minutes) sub-grouped by interventions.

Study or Subgroup	Mean Difference	SE	Mean Difference IV, Random, 95% CI
**GP Team Triage**			
van Gils-van 2018	4.52	23.9	4.52 [-42.32, 51.36]
**NP Team Triage**			
Day 2013	-37	0.94	-37.00 [-38.84, -35.16]
Gardner 2018	-34	4.8	-34.00 [-43.41, -24.59]
Hayden 2014	-16.65	23.9	-16.65 [-63.49, 30.19]
MacKenzie 2015	-20	1.13	-20.00 [-22.21, -17.79]
Pierce 2016	-28.5	23.9	-28.50 [-75.34, 18.34]
Rogers 2004	-22	23.9	-22.00 [-68.84, 24.84]
Tsai 2012	-58	0.83	-58.00 [-59.63, -56.37]
Tucker 2015	-18.3	23.9	-18.30 [-65.14, 28.54]
Zager 2018	-102	23.9	-102.00 [-148.84, -55.16]
**Nurse Triage-Plus**			
Adam 2014	-11	5.2	-11.00 [-21.19, -0.81]
Al-Abri 2020	-97	23.9	-97.00 [-143.84, -50.16]
Ashurst 2014	-6.5	23.9	-6.50 [-53.34, 40.34]
Cheung 2002	-46	23.9	-46.00 [-92.84, 0.84]
Demarco 2010	-193	23.9	-193.00 [-239.84, -146.16]
Dixon 2014	-55	23.9	-55.00 [-101.84, -8.16]
Fan 2006	-6.7	7.22	-6.70 [-20.85, 7.45]
Fontanel 2011	-64	23.9	-64.00 [-110.84, -17.16]
Hackman 2015	-10	4.03	-10.00 [-17.90, -2.10]
Ho 2018	-13	4.8	-13.00 [-22.41, -3.59]
Klassen 1993	-18	6.3	-18.00 [-30.35, -5.65]
Lee 1996	-18.6	23.9	-18.60 [-65.44, 28.24]
Lee 2014	-18.59	3.19	-18.59 [-24.84, -12.34]
Lee 2016	-28	23.9	-28.00 [-74.84, 18.84]
Li 2018	-15	0.96	-15.00 [-16.88, -13.12]
Parris 1997	-6	23.9	-6.00 [-52.84, 40.84]
Thurston 1996	-15	2.3	-15.00 [-19.51, -10.49]

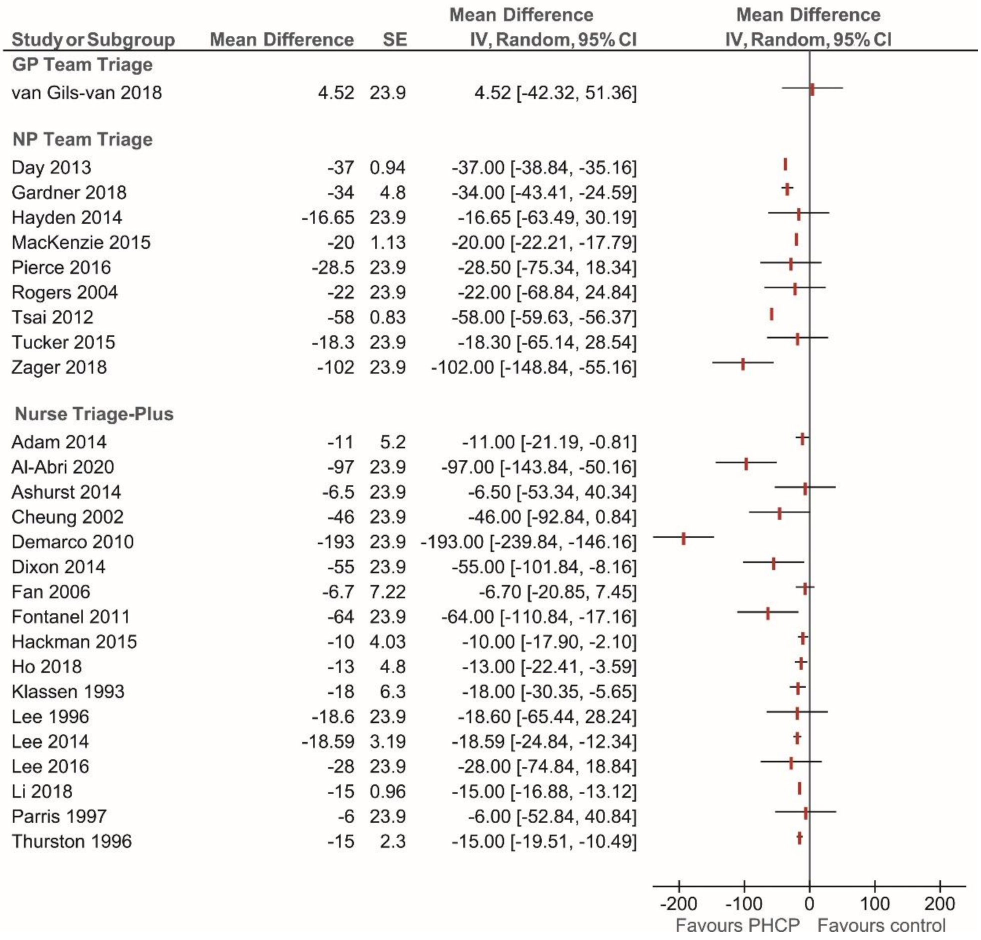

The horizontal black lines represent 95% confidence intervals and the red dots in the middle represents point estimates (mean difference).

References

1. Day TE, Al-Roubaie AR, Goldlust EJ. Decreased length of stay after addition of healthcare provider in emergency department triage: a comparison between computer-simulated and real-world interventions. *Emerg Med J.* 2013;30(2):134-138.
2. Hayden C, Burlingame P, Thompson H, Sabol VK. Improving patient flow in the emergency department by placing a family nurse practitioner in triage: a quality-improvement project. *Journal of Emergency Nursing.* 2014;40(4):346-351.
3. Lindley-Jones M, Finlayson BJ. Triage nurse requested x rays--are they worthwhile? *J Accid Emerg Med.* 2000;17(2):103-107.
4. Pierce BA, Gormley D. Are Split Flow and Provider in Triage Models in the Emergency Department Effective in Reducing Discharge Length of Stay? *Journal of Emergency Nursing.* 2016;42(6):487-491.
5. Tsai VW, Sharieff GQ, Kanegaye JT, Carlson LA, Harley J. Rapid medical assessment: improving pediatric emergency department time to provider, length of stay, and left without being seen rates. *Pediatric Emergency Care.* 2012;28(4):354-356.
6. Zager K, Taylor YJ. Discharge to medical home: A new care delivery model to treat non-urgent cases in a rural emergency department. *Healthcare.* 2018;21:21.
7. Lee WW, Filiatrault L, Abu-Laban RB, Rashidi A, Yau L, Liu N. Effect of Triage Nurse Initiated Radiography Using the Ottawa Ankle Rules on Emergency Department Length of Stay at a Tertiary Centre. *CJEM.* 2016;18(2):90-97.
8. Cheung WWH, Heeney L, Pound JL. An advance triage system. *Accident & Emergency Nursing.* 2002;10(1):10-16.
9. Love RA, Murphy JA, Lietz TE, Jordan KS. The effectiveness of a provider in triage in the emergency department: a quality improvement initiative to improve patient flow. *Advanced Emergency Nursing Journal.* 2012;34(1):65-74.
10. MacKenzie RS, Burmeister DB, Brown JA, et al. Implementation of a rapid assessment unit (intake team): impact on ED length of stay. *American Journal of Emergency Medicine.* 2015;33(2):291-293.
11. van Gils-van Rooij ESJ, Meijboom BR, Broekman SM, Yzermans CJ, de Bakker DH. Is patient flow more efficient in Urgent Care Collaborations? *European Journal of Emergency Medicine.* 2018;25(1):58-64.
12. Lijuan Z. Advanced Triage Protocols in the Emergency Department. *Advanced Triage Protocols in the Emergency Department.* 2017:1-1.
13. Adam H, Tamim H, Altamimi S, et al. Effect of triage nurse ordered distal extremity X-rays on emergency department length of stay: A randomized controlled trial. *Academic Emergency Medicine.* 2014;21(5 SUPPL. 1):S195.
14. Al Kadhi O, Manley K, Natarajan M, et al. A renal colic fast track pathway to improve waiting times and outcomes for patients presenting to the emergency department. *Open access emergency medicine : OAEM.* 2017;9:53-55.
15. Ashurst JV, Nappe T, Digiambattista S, et al. Effect of triage-based use of the Ottawa foot and ankle rules on the number of orders for radiographic imaging. *The Journal of the American Osteopathic Association.* 2014;114(12):890-897.
16. Demarco F, Gerardo CJ, Boardwine A, et al. Effect of a nurse rapid intake initiative on patient length of stay and satisfaction: A project IMPACT initiative. *Academic Emergency Medicine.* 2010;17(SUPPL. 1):S93-S94.

17. Dixon A, Clarkin C, Barrowman N, Correll R, Osmond MH, Plint AC. Reduction of radial-head subluxation in children by triage nurses in the emergency department: A cluster-randomized controlled trial. *CMAJ.* 2014;186(9):E317-E323.
18. Fan J, Woolfrey K. The effect of triage-applied Ottawa Ankle Rules on the length of stay in a Canadian urgent care department: a randomized controlled trial. *Academic emergency medicine : official journal of the Society for Academic Emergency Medicine.* 2006;13(2):153-157.
19. Fontanel A, Besson C, Gallegos C, Vallot C, Droal D. Can X-rays be prescribed by a nurse in an emergency department? *European Journal of Emergency Medicine.* 2011;18(5):310-311.
20. Gaucher N, Bailey B, Gravel J. Triage nurses' counselling influences return visits of children leaving the emergency department before being seen by a physician. *Academic Emergency Medicine.* 2010;17(SUPPL. 1):S118-S119.
21. Hackman JL, Roth ED, Gaddis ML, Gratton MC. The effect of a nurse-initiated chest pain protocol on disposition time: A retrospective review. *Annals of Emergency Medicine.* 2015;66(4 SUPPL. 1):S9-S10.
22. Jobé J, Vandercleyen C, Ghuysen A, D'Orio V. Prospective study of an advanced nurse triage for a target pathology at the admission in the emergency department. *Acta clinica belgica.* 2013;68(6):482-.
23. Klassen TP, Ropp LJ, Sutcliffe T, et al. A randomized, controlled trial of radiograph ordering for extremity trauma in a pediatric emergency department. *Annals of emergency medicine.* 1993;22(10):1524-1529.
24. Lee KM, Wong TW, Chan R, Lau CC, Fu YK, Fung KH. Accuracy and efficiency of X-ray requests initiated by triage nurses in an accident and emergency department. *Accident and emergency nursing.* 1996;4(4):179-181.
25. Lee WW, Filiatrault L, Abu-Laban RB, Rashidi A, Yau L, Liu N. Effect of triage nurse initiated radiography using the Ottawa Ankle Rules on emergency department length of stay at a tertiary care center. *Canadian Journal of Emergency Medicine.* 2014;16(SUPPL. 1):S38.
26. Sikkenga T, Dumkow L, Draper H, et al. Implementation of a nursing triage order to improve utilization of rapid diagnostic testing for chlamydia and gonorrhea in the emergency department. *Open Forum Infectious Diseases.* 2016;3(Supplement 1).
27. Al Abri FH, Muliira JK, Al Awaisi H. Effect of triage nurse-led application of the Ottawa Ankle Rules on number of radiographic tests and length of stay in selected emergency departments in Oman. *Japan journal of nursing science : JJNS.* 2020;17(1):e12270.
28. Ho JK-M, Chau JP-C, Chan JT-S, Yau CH-Y. Nurse-initiated radiographic-test protocol for ankle injuries: A randomized controlled trial. *International emergency nursing.* 2018;41:1-6.
29. Li Y, Lu Q, Du H, Zhang J, Zhang L. The Impact of Triage Nurse-ordered Diagnostic Studies on Pediatric Emergency Department Length of Stay. *Indian journal of pediatrics.* 2018;85(10):849-854.
30. Parris W, McCarthy S, Kelly AM, Richardson S. Do triage nurse-initiated X-rays for limb injuries reduce patient transit time? *Accid Emerg Nurs.* 1997;5(1):14-15.
31. Thurston J, Field S. Should accident and emergency nurses request radiographs? Results of a multicentre evaluation. *J Accid Emerg Med.* 1996;13(2):86-89.
32. Gardner RM, Friedman NA, Carlson M, Bradham TS, Barrett TW. Impact of revised triage to improve throughput in an ED with limited traditional fast track population. *American Journal of Emergency Medicine.* 2018;36(1):124-127.
33. Rogers T, Ross N, Spooner D. Evaluation of a 'see and treat' pilot study introduced to an emergency department. *Accident and Emergency Nursing.* 2004;12(1):24-27.
34. Tucker A, Bernard M. Making the Case for Nurse Practitioners in the Emergency Department: A Clinical Case Study. *Advanced Emergency Nursing Journal.* 2015;37(4):308-312.

35. Tsai VW, Sharieff GQ, Kanegaye JT, Carlson LA, Harley J. Rapid medical assessment: improving pediatric emergency department time to provider, length of stay, and left without being seen rates. *Pediatric emergency care.* 2012;28(4):354-356.
36. Uthman OA, Walker C, Lahiri S, et al. General practitioners providing non-urgent care in emergency department: a natural experiment. *BMJ Open.* 2018;8(5):e019736.
37. Kool RB, Homberg DJ, Kamphuis HC. Towards integration of general practitioner posts and accident and emergency departments: a case study of two integrated emergency posts in the Netherlands. *BMC Health Services Research.* 2008;8:225.
38. MacKenzie RS, Burmeister DB, Brown JA, et al. Implementation of a rapid assessment unit (intake team): impact on ED length of stay. *Am J Emerg Med.* 2015;33(2):291-293.
