# Supplementary material for: Impact of employing primary healthcare professionals in emergency department triage on patient flow outcomes: a systematic review and meta-analysis
Source: BMJ Open. 2022 Apr 19;12(4):e052850. doi: 10.1136/bmjopen-2021-052850 (PMC9058787; doi:10.1136/bmjopen-2021-052850)
Supplement: Author's manuscript [file bmjopen-2021-052850.draft_revisions.pdf]

BMJ Open is committed to open peer review. As part of this commitment we make the peer review history of every article we publish publicly available.

When an article is published we post the peer reviewers' comments and the authors' responses online. We also post the versions of the paper that were used during peer review. These are the versions that the peer review comments apply to.

The versions of the paper that follow are the versions that were submitted during the peer review process. They are not the versions of record or the final published versions. They should not be cited or distributed as the published version of this manuscript.

BMJ Open is an open access journal and the full, final, typeset and author-corrected version of record of the manuscript is available on our site with no access controls, subscription charges or pay-per-view fees (http://bmjopen.bmj.com).

If you have any questions on BMJ Open's open peer review process please email info.bmjopen@bmj.com

BMJ Open

The impact of employing primary healthcare professionals in emergency department triage on patient flow outcomes: A systematic review and meta-analysis

Journal:	*BMJ Open*
Manuscript ID	bmjopen-2021-052850
Article Type:	Original research
Date Submitted by the Author:	23-May-2021
Complete List of Authors:	Jeyaraman, Maya; University of Manitoba, George and Fay Yee Center for Healthcare Innovation<br>Alder, Rachel; Max Rady College of Medicine, Rady Faculty of Health Sciences, University of Manitoba<br>Copstein, Leslie; George and Fay Yee Center for Healthcare Innovation, University of Manitoba<br>Al-Yousif, Nameer; George and Fay Yee Center for Healthcare Innovation, University of Manitoba<br>Suss, Roger; Department of Family Medicine, Rady Faculty of Health Sciences, University of Manitoba<br>Zarychanski, Ryan ; Department of Medical Oncology and Hematology, Cancer Care Manitoba<br>Doupe, Malcolm; Department of Community Health Sciences, University of Manitoba, Winnipeg, Manitoba<br>Berthelot, Simon; Centre de recherche du CHU de Québec-Université Laval, Axe Santé des populations et Pratiques optimales en santé<br>Mireault, Jean; HEC Pôle santé, Université de Montréal<br>Tardif, Patrick; Department of Emergency Medicine, Cité de la santé de Laval<br>Askin, Nicole; WRHA Virtual Library, University of Manitoba<br>Buchel, Tamara; Manitoba College of Family Physicians<br>Rabbani, Rasheda; George and Fay Yee Center for Healthcare Innovation, University of Manitoba<br>Beaudry, Thomas; Patient and Public Engagement Collaborative Partnership, George & Fay Yee Center for Healthcare Innovation<br>Hartwell, Melissa; rimary and Integrated Health care Innovation Network<br>Shimmin, Carolyn; George and Fay Yee Center for Healthcare Innovation<br>Edwards, Jeanette; Community Health Quality and Learning, Shared Health Manitoba<br>Halas , Gayle; Manitoba Primary and Integrated Health care Innovation Network<br>Sevcik, William; Department of Emergency Medicine, Faculty of Medicine & Dentistry, University of Alberta<br>Tricco, Andrea; Knowledge Translation Program, St. Michael's Hospital Li Ka Shing Knowledge Institute, Unity Health Toronto<br>Chochinov, Alecs; epartment of Emergency Medicine, Faculty of Medicine, University of Manitoba<br>Rowe, Brian; Department of Emergency Medicine, Faculty of Medicine & Dentistry, University of Alberta; School of Public Health, University of

	Alberta<br>Abou-Setta, Ahmed; George and Fay Yee Center for Healthcare Innovation, University of Manitoba
Keywords:	ACCIDENT & EMERGENCY MEDICINE, INTENSIVE & CRITICAL CARE, Adult intensive & critical care < INTENSIVE & CRITICAL CARE

SCHOLARONE™ Manuscripts

I, the Submitting Author has the right to grant and does grant on behalf of all authors of the Work (as defined in the below author licence), an exclusive licence and/or a non-exclusive licence for contributions from authors who are: i) UK Crown employees; ii) where BMJ has agreed a CC-BY licence shall apply, and/or iii) in accordance with the terms applicable for US Federal Government officers or employees acting as part of their official duties; on a worldwide, perpetual, irrevocable, royalty-free basis to BMJ Publishing Group Ltd ("BMJ") its licensees and where the relevant Journal is co-owned by BMJ to the co-owners of the Journal, to publish the Work in this journal and any other BMJ products and to exploit all rights, as set out in our licence.

The Submitting Author accepts and understands that any supply made under these terms is made by BMJ to the Submitting Author unless you are acting as an employee on behalf of your employer or a postgraduate student of an affiliated institution which is paying any applicable article publishing charge ("APC") for Open Access articles. Where the Submitting Author wishes to make the Work available on an Open Access basis (and intends to pay the relevant APC), the terms of reuse of such Open Access shall be governed by a Creative Commons licence – details of these licences and which Creative Commons licence will apply to this Work are set out in our licence referred to above.

Other than as permitted in any relevant BMJ Author's Self Archiving Policies, I confirm this Work has not been accepted for publication elsewhere, is not being considered for publication elsewhere and does not duplicate material already published. I confirm all authors consent to publication of this Work and authorise the granting of this licence.

For peer review only

The impact of employing primary healthcare professionals in emergency department triage on patient flow outcomes: A systematic review and meta-analysis

Review Authors: Maya M. Jeyaraman[1], Rachel N. Alder[2], Leslie Copstein[1], Nameer Al-Yousif[1], Roger Suss[3], Ryan Zarychanski[4], Malcolm Doupe[5], Simon Berthelot[6], Jean Mireault[7], Patrick Tardif[8], Nicole Askin[9], Tamara Buchel[10], Rasheda Rabbani[1], Thomas Beaudry[11], Melissa Hartwell[12], Carolyn Shimmin[1], Jeanette Edwards[13], Gayle Halas[14], William Sevcik[15], Andrea C. Tricco[16], Alecs Chochinov[17], Brian H. Rowe[15,18], Ahmed M. Abou-Setta[1]

Affiliations:

[1] George & Fay Yee Center for Healthcare Innovation, University of Manitoba, Winnipeg, Manitoba;

[2] Max Rady College of Medicine, Rady Faculty of Health Sciences, University of Manitoba, Winnipeg, Manitoba;

[3] Department of Family Medicine, Rady Faculty of Health Sciences, University of Manitoba, Winnipeg, Manitoba;

[4] Department of Medical Oncology and Hematology, Cancer Care Manitoba, Winnipeg, Manitoba;

[5] Department of Community Health Sciences, University of Manitoba, Winnipeg, Manitoba;

[6] Centre de recherche du CHU de Québec-Université Laval, Axe Santé des populations et Pratiques optimales en santé, Laval, Québec;

[7] HEC Pôle santé, Université de Montréal, Montreal, Quebec;

[8] Department of Emergency Medicine, Cité de la santé de Laval, Laval, Quebec;

[9] WRHA Virtual Library, University of Manitoba, Winnipeg, Manitoba;

[10] Manitoba College of Family Physicians, Winnipeg, Manitoba;

[11] Patient and Public Engagement Collaborative Partnership, George & Fay Yee Center for Healthcare Innovation, Winnipeg, Manitoba;

[12] Primary and Integrated Health care Innovation Network, Edmonton, Alberta;

[13] Community Health Quality and Learning, Shared Health Manitoba, Winnipeg, Manitoba;

[14] Manitoba Primary and Integrated Health care Innovation Network, Winnipeg, Manitoba;

[15] Department of Emergency Medicine, Faculty of Medicine & Dentistry, University of Alberta, Edmonton, Alberta;

For peer review only - http://bmjopen.bmj.com/site/about/guidelines.xhtml

For peer review only

[16] Knowledge Translation Program, St. Michael's Hospital Li Ka Shing Knowledge Institute, Unity Health Toronto, Toronto, Ontario;

[17] Department of Emergency Medicine, Faculty of Medicine, University of Manitoba, Winnipeg, Manitoba;

[18] School of Public Health, University of Alberta, Edmonton, Alberta;

all in *Canada.*

.

Corresponding author: Maya Jeyaraman, MD PhD
George & Fay Yee Center for Healthcare Innovation
753 McDermot Ave, Winnipeg MB R3E 0T6
Email: maya.jeyaraman@umanitoba.ca
Phone: (204) 594-5362; Fax: (204) 594-5394.

Short Title: Impact of employing primary healthcare professionals in emergency department triage.

Key Words: ED overcrowding, ED Patient flow, ED Triage, Primary healthcare provider

Total word count: 4277 words; **Abstract**: 352 words**; Figures**: 4; **Tables**: 3

For peer review only

Abstract

Objectives: To identify, critically-appraise and summarize evidence on the impact of employing primary healthcare professionals (PHCPs: family physicians/general practitioners (GP), nurse practitioners (NP) and nurses with increased authority) in the emergency department (ED) triage, on patient flow outcomes.

Methods: We searched Medline (Ovid), EMBASE (Ovid), Cochrane Library (Wiley) and CINAHL (EBSCO) (inception to January 2020). Our primary outcome was the time to provider initial assessment (PIA). Secondary outcomes included time to triage, proportion of patients leaving without being seen (LWBS), length of stay (ED LOS), proportion of patients leaving against medical advice (LAMA), number of repeat ED visits, and patient satisfaction. Two independent reviewers selected studies, extracted data, and assessed study quality using the NICE quality assessment tool.

Results: From 23,973 records, 40 comparative studies including 10 randomized controlled trials (RCTs) and 13 pre-post studies were included. PHCP interventions were led by NP (n=14), GP (n=3) or nurses with increased authority (n=23) at triage. In all studies PHCP-led intervention effectiveness was compared to the traditional nurse-led triage model. Median duration of the interventions was 6 months. Study quality was generally low (confounding bias); 7 RCTs were classified as moderate quality. Most studies reported that PHCP-led triage interventions decreased the PIA (13/14), ED LOS (29/30), proportion of patients LWBS (8/10), time to triage (3/3), and repeat ED visits (5/6), and increased the patient satisfaction (8/10). The proportion of patients LAMA did not differ between groups (3/3). Evidence from RCT's (n=8) as well as other study designs showed a significant decrease in ED LOS favoring the PHCP-led interventions.

For peer review only - http://bmjopen.bmj.com/site/about/guidelines.xhtml

Conclusions: Overall, PHCP-led triage interventions improved ED patient flow metrics. There was a significant decrease in ED LOS irrespective of the study design, favoring the PHCP-led interventions. Evidence from well-designed high quality RCTs is required prior to widespread implementation.

PROSPERO trial registration number: CRD42020148053

For peer review only

For peer review only - http://bmjopen.bmj.com/site/about/guidelines.xhtml

Article Summary

Strengths and limitations of this study

- The main strength of our systematic review is that our study team engaged and collaborated with patient and public partners during the design, conduct and dissemination phases of the study by following the criteria identified for patient-oriented research which emphasizes the active and meaningful engagement of patients as research partners.
- This systematic review was conducted using the rigorous Cochrane systematic review methodology and used an a priori registered protocol.
- A limitation of this systematic review is that we did not include non-English language publications.

For peer review only

For peer review only - http://bmjopen.bmj.com/site/about/guidelines.xhtml

INTRODUCTION

Healthcare systems worldwide experience emergency departments (ED) overcrowding[1-5] which impacts the timely delivery of healthcare[6,7], patient and provider dissatisfaction[8], and other adverse outcomes[9]. ED overcrowding is a complex phenomenon and is associated with input (increased patient volume), throughput (ED boarding), and especially output (lack of hospital beds) factors, as well as system-wide influences[10]. A large volume of lower acuity patients presenting to ED leads to demand-capacity mismatch and entry block (e.g., delays in ED assessment)[10,11].

Lower acuity ED patients generally include patients: a) having low acuity triage codes; b) being discharged quickly; or c) being seen by an alternative primary healthcare provider[12]. These alternative primary healthcare providers are typically physicians (family physicians/general practitioners [GP]), nurse practitioners (NP), nurses with increased authority, or physician assistants who are legally authorized to provide or coordinate healthcare to patients[13]. Studies have reported that 8-62% of all ED presentations are lower acuity[14-17]. With ED visits increasing by 20% each year, along with a decrease in operational EDs[18], lower acuity visits may lead to unnecessary diagnostic testing, greater healthcare spending, lost opportunity for continuity of care with primary care physicians, sub-optimal care due to hurried management, and prolonged ED length of stay (LOS)[12,14,19,20]. Increased demand for ED services also leads to increased ED wait times and patients choosing to leave ED without being-seen, thus potentially compromising patient safety[18].

Worldwide, there is growing interest in interventions and strategies, either to discourage lower acuity ED visits or to reduce the impact of lower acuity visits in the ED by improving patient flow. Studies have investigated the impact of interventions such as public and patient

For peer review only - http://bmjopen.bmj.com/site/about/guidelines.xhtml

education[14], financial disincentives (higher co-payments for lower acuity ED visits)[21], increasing after-hours primary care[22], patient redirection to non-ED care alternatives[23], and advanced access[24,25] to discourage unnecessary ED utilization. Since EDs have no control over the volume of presenting patients and ED presentations continue to be on the rise[14,18], recommendations have been made to focus on strategies to improve patient flow within the ED[26]. Studies have investigated various strategies to improve ED patient flow, including triage related interventions[8,27].

While the precise role of the NPs, GPs or nurses given increased authority (all referred to as primary healthcare professionals [PHCPs]) in an ED is unclear, they may provide potential benefits to improve ED times and outcomes. Although, many primary research studies have investigated the impact of PHCP's[20,28-33] at triage on ED patient flow, to the best of our knowledge there are no systematic reviews that have summarized evidence from these studies.

The main objective of our systematic review was to identify, critically-appraise and summarize evidence on the effectiveness of employing PHCP's at ED triage to improve ED patient flow metrics.

For peer review only

For peer review only - http://bmjopen.bmj.com/site/about/guidelines.xhtml

METHODS

Using an *a priori* systematic review protocol (CRD42020148053) developed in collaboration with patient partners, we conducted this review according to guidelines enumerated in the Methodological Expectations of Cochrane Intervention Reviews (MECIR). Our systematic review is reported using the Preferred Reporting Items for Systematic reviews and Meta-Analyses (PRISMA) guideline[34].

Eligibility criteria

We included comparative studies (only English language) of any ED triage intervention that involved a PHCP and was designed to improve ED (adult and pediatric) patient flow metrics. We excluded primary studies involving exclusively emergency physicians (ED MD), such as the triage liaison physician (TLP)[27]. The primary outcome was the time to provider initial assessment (PIA: time from ED arrival to the time when the patient is first assessed by an ED provider (ED MD, NP, or a GP in the ED)). Secondary outcomes were ED length of stay (LOS: time from ED arrival to disposition), the proportion of patients who left without being seen (LWBS), proportion of patients leaving against medical advice (LAMA), time to triage, number of repeat ED visits, and patient satisfaction. The outcome measures were selected *a priori* in collaboration with the patient partners in the research team. We have reported a more detailed list of the inclusion and exclusion criteria in ***Appendix Table 1***.

Literature search methods for identifying relevant citations

In conjunction with a health librarian (TR) we designed a search strategy for Medline (Ovid) to identify literature relevant to the objective (from inception until June 2018, and later updated in

For peer review only - http://bmjopen.bmj.com/site/about/guidelines.xhtml

January 2020). Since most of the potentially relevant studies would be performed in the US, Europe and Commonwealth countries, search results were limited to English language publications. Our Medline search was peer-reviewed by a second librarian (JJ)[35], principal investigators (MJ, AMAS), and patient partners (MH, TB). Once finalized, the Medline search strategy (***Appendix Table 2***) was adapted for replication in the following databases: EMBASE (Ovid), Cochrane Library (Wiley), and CINAHL (EBSCO). An experienced librarian (NA) searched the included databases up to January 2020. The bibliographic search was supplemented with searching the grey literature (i.e., difficult to locate unpublished studies) as listed in ***Appendix Table 3.*** We also searched the reference lists of all the included publications for additional relevant studies. We used EndNote™ (Version X7, Thomson Reuters) for reference management.

Selection of sources of evidence

Two reviewers (RA & (LC or NA)) independently screened the titles and abstracts, and full texts of relevant citations using pilot tested screening forms. Any disagreement on inclusion was resolved through consensus or third party (MJ) adjudication.

Data Extraction, Data analysis and Quality assessment

Standardized data extraction forms were developed to record data from each of included studies after pilot testing. At least two review authors independently extracted baseline characteristics (RA, LC, NA), outcome data (RA, LC) and assessed methodological quality (MJ, RA, LC) on these studies. Disagreements among reviewers were resolved through consensus or third-party adjudication (MJ or AMAS). A meta-analysis of mean differences (MD) in ED times with 95%

For peer review only - http://bmjopen.bmj.com/site/about/guidelines.xhtml

confidence intervals (CIs) was planned *a priori* to derive pooled summary estimates. Heterogeneity among included studies was quantified and tested using I-squared (I^2) statistic and chi-squared statistic, respectively. An I^2 value >50% was considered high heterogeneity; we made an *a priori* methodological decision that heterogeneity indicated by I^2 > 50% was too high to justify data pooling to generate a summary measure. For studies that did not report any measure of variance we imputed the largest standard error (SE) from among the included studies. In the event that meta-analysis was not possible, the effect estimates (mean differences and SE) from included studies reporting data for the primary outcome and ED LOS were depicted in the form of a forest plot for various a priori subgroups (study designs or PHCP interventions). In these cases, where appropriate, the median of the primary study outcome was reported as the average measure.

We assessed the included studies using the National Institute for Health and Care Excellence (NICE) quality appraisal tool for quantitative studies of intervention[36] as it can be used for multiple study designs. A detailed description is reported under ***Appendix methods***.

Patient and Public Involvement

We collaborated with a diverse group of 13 patient partners (self-identified as Indigenous, Immigrant, White and/or living with disability) during the design phase and the conduct phase of this project, to refine the review question, refine the inclusion criteria, and select patient-important outcomes. Two (TB, MH) of these patient partners collaborated and supported our grant application to obtain funding for this project. During the conduct phase of this systematic review three patient partners helped refine the search strategy (by identifying missing search terms and suggesting additional search terms in the preliminary search strategy), review to

For peer review only - http://bmjopen.bmj.com/site/about/guidelines.xhtml

confirm included studies, and in knowledge dissemination (co-presented abstract at a conference and co-authoring the manuscript). We have reported the patient partner involvement in this systematic review according to GRIPP2 checklist (short form)[37].

For peer review only

For peer review only - http://bmjopen.bmj.com/site/about/guidelines.xhtml

RESULTS

We identified 23,973 relevant citations from database search, of which 40 met the inclusion criteria[18,20,26,28,29,32,33,38-70] (44 study reports). The study selection process is reported using the PRISMA study flow chart (***Figure 1***).

Study characteristics

Included studies were full-length journal articles[18,20,26,28,29,32,33,39-43,45,46,48,51,53-55,57,58,60-63,65-70] (n = 31; 77.5%), abstracts (n=8)[38,44,47,49,50,52,56,64] or thesis (n=1)[59] published from 1993 to 2020. More than half were conducted in North America[18,26,28,41-43,45,46,48-50,53,56,57,59,61,63,64,66,67,70] (n = 21; 52.5%), and the rest were conducted in Europe[20,32,33,40,47,52,54,60,65,68,69] (n = 11; 27.5%), Asia[51,55,58] (n = 3; 7.5%), Australia[29,62] (n = 2; 5%), Middle East[38,39] (n = 2; 5%) or the location was not reported[44] (n = 1; 2.5%) (***Table 1***). Most studies utilized a pre-post intervention design[18,26,29,32,33,42,43,48,59,61,66,67,70] (n = 13; 32.5%) and the remaining were characterized as randomized controlled trials (RCT)[38,45,46,51-53,56,57,60,65] (n = 10; 25%), observational retrospective cohort studies[20,28,40,49,50,58,64,68] (n = 8, 20%), controlled before and after studies (CBA)[39,47,54,63] (n = 4; 10%), quasi-randomized trials[62] (n = 1; 2.5%) observational prospective cohort studies[41,44,55] (n = 3; 7.5%), or cross-sectional observational studies[69] (n = 1; 2.5%). The median duration of intervention reported among included studies was 6 months (range: 2.5 days to 17 months). Studies were mostly conducted in urban EDs[20,26,28,29,32,33,38,39,41-46,48,50,53,55-57,60,62-68] (n = 28; 70%), one (2.5%) was conducted in a rural ED and two[54,69] (5%) were conducted in a combination of urban and rural facilities; nine[18,40,47,49,51,52,58,59,61] (22.5%) studies did not report their setting. We classified the EDs reported in the included studies into pediatric EDs (age < 18)[38,45,49,53,58,66] (n = 6; 15%), mixed EDs seeing both adults and children[18,29,41,47,51,55] (n = 6; 15%) and adult only EDs[39,42,48,57,62] (n = 5; 12.5%), depending on the age of the population that

For peer review only - http://bmjopen.bmj.com/site/about/guidelines.xhtml

the EDs served. More than half of the studies (n = 23, 57.5%) did not specifically report the age of the population that their EDs served[20,26,28,32,33,40,43,44,46,50,52,54,56,59-61,63-65,67-70]. The majority (n = 15, 38%) of included studies[20,29,32,33,39,42,48,51,54,58,62,65,67,68,70] reported enrolling only patients with triage category 4-5 (additional details reported in ***Appendix Results 1***).

The majority (82.5%) of included studies were of low methodological quality and the remaining seven (17.5%) included studies (RCTs)[38,46,51,56,57,60,65] were of a moderate methodological quality (***Table 1 & Appendix Table 4***).

We categorized the triage interventions involving PHCP reported by the included studies, in comparison to the traditional (nurse-led) triage model (***Figure 2),*** as follows: (1) *GP team-triage*[54,68,69] *(n = 3, 7%)*: where GP was involved in the ED triage (triaging or supervising triage) either seeing and treating low-acuity patients or streaming moderate to high-acuity patients to the ED MD; (2) *NP team-triage*[18-20,26,29,32,33,42,43,48,63,67,70,71] *(n =14, 35%):* where the NP was located at the ED triage area working alongside a triage nurse, either ordering investigations at triage before streaming to ED MD, seeing and treating low-acuity patients, directing low-acuity patients to a GP located within ED for treatment, or assessing patients and discharging/re-directing with a same day appointment with a GP at an adjoining GP clinic; (3) *Nurse triage-plus*[28,38-41,44-47,49-53,55-60,62,64,65] *(n = 23, 58%)*: triage nurse with increased authority (extra capacities outside of their usual scope of practice) to order investigations for patients before streaming to the ED MD. The traditional ED care model with an ED nurse-led triage followed by the ED MD assessment was considered standard of care and the comparator in all the included studies (***Figure 2)***.

For peer review only - http://bmjopen.bmj.com/site/about/guidelines.xhtml

PIA

Fourteen studies[18,26,32,39,42,48,51,54,60,61,63,66,67,69] (35%) reported the effect of PHCP triage interventions on PIA in comparison to a traditional nurse-led triage. Using a forest plot, we depicted the effectiveness of the PHCP triage interventions on PIA sub-grouped by study design (***Figure 3***). Two RCTs[51,60] (of moderate quality), reported a non-significant small decrease in PIA in the PHCP triage intervention (nurse triage-plus) group compared to the traditional nurse-led triage model (mean difference (MD) -0.36 minutes [95% CI -4.53 to 3.81]; 2 studies; I^2: 39%; P=0.20; moderate quality). Three CBA studies[39,54,63] (low quality) reported a decrease in PIA in the PHCP triage intervention group (median [range] = -18 minutes [-2.3 to -31]).

All eight pre-post studies[18,26,32,42,48,61,66,67] (low quality) reported a significant decrease in PIA (median [range] = -24.65 minutes [-3 to -50]) in the PHCP triage intervention group, compared to the traditional nurse-led triage model. Exploration of heterogeneity among pre-post studies (I^2: 100%) revealed four studies[26,42,48,61] that contributed to all the observed heterogeneity; however, we were unable to identify specific reasons for heterogeneity. A sensitivity analysis without these four studies showed a significant mean decrease of PIA by 26 minutes favoring the PHCP triage intervention group (NP team triage). One cross-sectional observational study[69] (low quality) failed to identify a difference in PIA in the PHCP intervention group (4.4 minutes). The results for PIA sub-grouped by various PHCP interventions is reported under ***Appendix Results 2.*** We have depicted the effectiveness of each of the three models of PHCP triage interventions on PIA separately using a forest plot (***Appendix Figure 1***).

Three studies[26,29,32,33] reported greater percentage of patients seen within benchmark times in the NP team triage intervention groups compared to the traditional nurse-led triage

For peer review only - http://bmjopen.bmj.com/site/about/guidelines.xhtml

model (***Appendix Figure 2***). A fourth study[29] reported that greater percentage of patients (all ATS categories) were seen within benchmark times in the NP team triage group compared to the traditional nurse-led triage group (data not shown).

ED LOS

ED LOS was reported by thirty studies (75%)[18,19,28,32,38-41,43-48,50-53,55-58,62-65,67,69-71]. Using a forest plot, we have depicted the effectiveness of the PHCP triage interventions on ED LOS sub-grouped by study design (***Figure 4***). Eight RCTs[38,45,46,51,53,56,57,65] (six[38,46,51,56,57,65] of moderate quality and two[45,53] of low quality), reported a significant decrease in ED LOS (MD -15.31 minutes [95% CI -18.35 to -12.27]; 8 studies; I^2: 0%; P<0.00001) in the PHCP triage intervention (nurse triage-plus) group compared to the traditional nurse-led triage model. The CBA studies[39,47,63] (low quality) reported a significant decrease in ED LOS (mean difference -63.17 minutes [95% CI -101.93 to -24.40]; 3 studies; I^2: 51%; P=0.001) in the PHCP triage intervention group (2 nurse-triage plus and one NP team triage) compared to the traditional nurse-led triage model, and the three retrospective cohorts[28,50,58] (low quality) also reported a significant decrease in the ED LOS (MD -13.96 minutes [95% CI -19.31 to -8.61]; 3 studies; I^2: 37%; P<0.00001) in the PHCP triage intervention group (nurse triage-plus), compared to the traditional nurse-led triage model.

Among eight pre-post studies[18,32,43,48,61,66,67,70] (low quality), all reported a decrease (5 were significant) in ED LOS (median [range] = -28 minutes [-16.65 to -102) favoring the PHCP triage intervention group (NP team triage). Exploration of heterogeneity among pre-post studies (I^2: 99%) revealed four studies[43,48,66,70] that contributed to all the observed heterogeneity; however, we were unable to identify specific reasons for heterogeneity. A sensitivity analysis

For peer review only - http://bmjopen.bmj.com/site/about/guidelines.xhtml

without these four studies showed a significant mean decrease of ED LOS by 17 minutes favoring the PHCP triage intervention group (NP team triage). One quasi-RCT[62], and one cross-sectional observational study[69] reported no significant differences in ED LOS between comparison groups. Among the three prospective observational cohorts[41,44,55], one reported significant decrease in ED LOS whereas other two reported a non-significant decrease in ED LOS favoring PHCP intervention group. The ED LOS sub-grouped by various PHCP interventions is reported under ***Appendix Results 3***. We have depicted the effectiveness of each of the three models of PHCP triage interventions on ED LOS separately, using a forest plot (***Appendix Figure 1***).

Other outcomes

Ten studies[18-20,26,42,48,58,59,67,71] reported data for percentage of patients LWBS (***Table 2).*** Eight studies reported a reduction in percentage of patient LWBS in the NP team triage intervention group, except one[18] (five[19,20,26,48,71] reported statistically significant decrease). Two[58,59] studies reported a non-significant decrease in percentage of patients LWBS in nurse triage-plus intervention group. The median effect of all estimates is a reduction in LWBS of -2.31% (IQR: -0.39, -3.77). Three pre-post studies[18,67,71] reported the effect of NP team triage intervention on percentage of patients discharged as LAMA (***Table 3***). One study showed a non-significant decrease favoring the intervention group and another two showed a non-significant increase in the percentage of patients LAMA.

Six studies[43,49,54,67,68,70] reported the impact of PHCP interventions on the number of repeat ED visits, and the majority of them reported a decrease in the number of repeat ED visits after PHCP intervention (***Appendix Results 4***). Ten studies[18,41,44,46,48,54,56,57,59,67] reported the effect of PHCP intervention on patient satisfaction, and the majority of them reported an increase

For peer review only - http://bmjopen.bmj.com/site/about/guidelines.xhtml

in patient satisfaction (***Appendix Results 5***). Three studies[32,51,71] reported the impact of PHCP interventions on the time to triage, and all of them reported a decrease in the time to triage after PHCP intervention (***Appendix Results 6***).

For peer review only

For peer review only - http://bmjopen.bmj.com/site/about/guidelines.xhtml

DISCUSSION

This systematic review has summarized the best available evidence from 40 unique comparative studies on the effectiveness of the PHCP triage interventions to improve ED patient flow metrics and mitigate the negative impacts of ED overcrowding. The findings in this systematic review shows that the PHCP-led triage interventions significantly decrease the ED LOS and lead to improvements in key ED patient flow metrics such as PIA, proportion of patients who LWBS, triage time, ED visits and patient satisfaction.

Although this systematic review highlights the positive impact of three unique PHCP triage models on key ED patient flow metrics, it is important to note that the most comprehensive evidence (data for the primary review outcome and all of the secondary outcomes) was available mainly for the nurse triage-plus and NP team triage models, with the least evidence available for the GP team triage model.

To the best of our knowledge this is the first review to investigate specific triage interventions involving NPs and GPs. Previous work had focussed specifically on the impact of TLP[27] or triage nurse ordering[72] on ED patient flow metrics. In 2011, Rowe et al.[27] investigated the impact of TLP's and reported reductions in ED LOS and PIA. However, the interventions mainly involved emergency physicians. Previously, Jennings et al[73] published a systematic review on the impact of emergency NP services in the ED and narratively concluded that although not enough data were available for meta-analysis, NPs within ED may have a positive impact on waiting times, patient satisfaction, and quality of care. Again, this review did not focus on NP at triage. A recent Cochrane review investigated the role of primary care professionals (emergency NP and GP) in the ED[74] and concluded that due to limited evidence and suspected bias in allocations of ED patients it was unclear if hiring primary care professionals would

For peer review only - http://bmjopen.bmj.com/site/about/guidelines.xhtml

decrease PIA, ED LOS, and other ED metrics. It is important to note, however, that this Cochrane review did not investigate the role of primary care professionals at ED triage.

ED wait times for care delivery is a key performance indicator in many ED settings and our systematic review findings indicate that the PHCP-led triage intervention consistently decreases ED wait times (PIA) and ED LOS. In this review, pre-post studies contributed to the majority of the evidence for effectiveness of PIA (NP team triage). Although heterogeneous and of low quality, the results indicate important potential for the role of NPs in the triage process to reduce ED wait times, improve patient satisfaction and other key ED metrics. A significant decrease in ED LOS was observed with the RCTs (median: -16.8 minutes) although this was comparatively smaller than the significant decrease observed with the CBA (median: -64 minutes) or the pre-post studies (median: -28 minutes). As the minimal clinically important difference in ED LOS is generally accepted to be approximately 30 minutes (clinically significant), the PHCP-led triage interventions could potentially have a positive impact on ED LOS, if implemented.

One may argue that similar results could be seen with ED MD at triage. Although true, the cost of adding an NP could be far less than adding an ED MD[67]. In our review, we found only three studies reporting evidence on the role of GP's in ED triage, with one CBA study[54] reporting statistically significant decrease in PIA, and a cross-sectional study[69] reporting an increase in PIA when triaged and treated by a GP. The increase in PIA, however, was reported to be due to an increase in the number of self-referrals in order to be seen by the GP involved in triage and treatment of low-acuity patients[69]. In the reported GP team triage interventions, an ED and a GP clinic were co-located and had a joint common entrance, with the GP assistant (supervised by GP) and/or a GP being responsible for the triage of patients (for both ED and GP clinic) and for the treatment of low-acuity patients. The third study[68] reported that GP team triage

For peer review only - http://bmjopen.bmj.com/site/about/guidelines.xhtml

and x-ray requests at the joint triage reduced the annual ED patient visits. High-quality studies investigating the effectiveness of GPs at ED triage would be valuable.

In our review, the evidence on effectiveness of nurse triage-plus model came mostly from moderate quality studies (RCT or CBA) and showed significant decrease in PIA, ED LOS, and an improved patient satisfaction. Many factors such as patient acuity, EMS traffic/volume and referral patterns often dictate the degree of ED crowding and each ED has their own “signature”. For example, in settings where most patients present with ambulatory, single system problems, a nurse triage with extra skills might be effective. Conversely, a nurse triage-plus intervention may be less effective when faced with the challenges of an ED setting with high volumes of trauma, EMS traffic, and high acuity patients.

It would be generally expected that the addition of any qualified staff in the ED, including addition of NP in triage, would tend to make efficiency of the ED operations better. Although we did not assess staff satisfaction in our review, it would be intuitive to think that the addition of NP in the ED triage may also help improve ED staff satisfaction. Many government-funded EDs are cash-constrained and often cannot add additional resource without strong justification and/or reducing funding elsewhere. While addition of NPs, TLPs, or GPs at triage may help, there is still lack of published comparative effectiveness and economic evaluation research to produce a clear cost-effectiveness recommendation. While comparative effectiveness research may prove logistically difficult in the ED and outcome measurements need to be granular and robust (e.g., including intended and unintended consequences), these studies are critical to developing recommendations.

Overall, the evidence synthesized by our review indicates that the PHCP-led triage interventions significantly decrease PIA or ED LOS compared to the traditional nurse-led triage

For peer review only - http://bmjopen.bmj.com/site/about/guidelines.xhtml
For peer review only

model. The studies in this review demonstrate promise to improve ED patient flow metrics by either seeing and treating non-urgent patients in the triage area, starting investigations at triage for moderate to low acuity patients, or assessing and making decision to re-direct very low-acuity patients to an adjoining GP clinic with same day appointments. All of these could mitigate ED overcrowding. Since ED wait times are multi-factorial it cannot be expected that one solution will solve such a complex problem. Each ED will need an individualized approach. Moreover, while calling for improved research quality, we believe comparative effectiveness studies with health economic outcomes are required to fully weigh the costs and benefits associated with any intervention.

We acknowledge the following limitations in interpreting the results of this systematic review. All systematic reviews are susceptible to publication and selection bias. Selection bias was minimized by using a comprehensive, peer-reviewed search strategy developed by an experienced information specialist. Selection bias was also addressed by using two independent reviewers and third-party adjudication. We evaluated the quality of each included study using the NICE Quality Appraisal Tool[36]; that is tailored to quantitative studies investigating public health interventions. A few included studies reported the effectiveness of GP team triage intervention on the review outcomes, thus limiting conclusions on GP-led triage interventions. Most of the included studies were of pre-post intervention design providing low quality evidence. Even the included RCT's were only of moderate quality, thus evidence from high quality studies is lacking, limiting the confidence that can be placed on the results. Nevertheless, irrespective of the study design, we observed a significant decrease in ED LOS favoring PHCP-led triage intervention. Although we used a comprehensive search strategy, for the sake of feasibility we did not consider non-English language studies and the possibility of missing some of the other

For peer review only - http://bmjopen.bmj.com/site/about/guidelines.xhtml

language studies remains. Despite the compressive search strategy, publication bias is likely since many operational studies never reach publication and many of those would be negative. We also encountered issues with missing data in some of the included studies and resorted to imputation techniques as we were unable to obtain data from study authors. As included studies were conducted in various countries, health systems and societal contexts, the results from one may not be compatible with evidence from other jurisdictions.

Notwithstanding the above concerns, we believe this review has many strengths, including the rigorous Cochrane systematic review methodology employed and the use of an *a priori* registered protocol. In addition, our study team included patient partners who collaborated with the investigators during the design, conduct and dissemination phases of the study. Following the criteria identified for patient-oriented research which emphasizes the active and meaningful engagement of patients as research partners, twelve diverse group of patient partners from three Canadian provinces (Manitoba, Alberta, and Quebec) were engaged from the design stage and throughout the research process around decisions and in knowledge dissemination.

CONCLUSIONS

PHCP-led triage interventions could be an effective strategy to improve ED patient flow overall by decreasing ED LOS, PIA, time to triage or ED visits, and by improving patient satisfaction. While these triage interventions may work in specific settings, each ED is unique, and policy would have to be evaluated specific to that facility and system. High quality methods are also necessary to further support PHCP's role in ED triage, and it is important for future studies to focus on cost efficiency or incremental value for money as these are critical real-world issues. Additionally, future research could focus on generating high quality evidence on the

For peer review only - http://bmjopen.bmj.com/site/about/guidelines.xhtml

effectiveness of GP triage intervention. The acceptability of a PHCP-led interventions in an ED could also be formally ascertained in future studies as experience and beliefs of ED staff may play a role in the success or failure of the policy to implement PHCPs in triage. Finally, the research gap involving rural EDs needs to be addressed.

Acknowledgements

We are very grateful for the Canadian Institutes of Health Research (CIHR) grant (NKS 158643) and the Manitoba Medical Services Foundation (#8-2018-06) and Winnipeg Foundation (#8-2018-06) for providing financial support for this project. Dr. Rowe's research was supported by a Scientific Director's Grant (SOP 168483) from CIHR. Dr. Tricco's research is funded by a Tier 2 Canada Research Chair in Knowledge Synthesis. We are also very grateful to all the patient partners who collaborated with us throughout the duration of this project. We thank Dr. Cristina Villa-Roel for her support during grant and manuscript preparation and the Emergency Medicine Research Group (EMeRG) in the Department of Emergency Medicine at the University of Alberta for in-kind resources. We are very grateful to all the patient partners who collaborated with us in the design, conduct and dissemination stages of this project. We are thankful to Frank Krupka for the kind support of this project during his time as the Executive Director of CHI. We thank Tamara Rader, Christine Nielson, and Janet Joyce for help with the search strategy for this study. We thank our partner organizations (Strategy for Patient-Oriented Research Support for People and Patient-Oriented Research and Trials units [Manitoba, Alberta and Quebec], Emergency Medicine Research Group [EMeRG], and Primary and Integrated Healthcare Innovations network, and the knowledge users (Winnipeg Regional Health Authority,

For peer review only

For peer review only - http://bmjopen.bmj.com/site/about/guidelines.xhtml

Emergency Strategic Clinical Network, Shared Health Manitoba, and Manitoba College of Family Physicians) for their kind support and feedback throughout the duration of this project.

Funding: This work was supported by research grant from Canadian Institutes of Health Research (NKS 158643), Manitoba Medical Services Foundation (# 8-2018-06) and Winnipeg Foundation (# 8-2018-06).

Competing interests: None declared.

Data sharing statement: All data relevant to the study are included in the article or uploaded as supplementary information.

Ethics Approval: Not applicable. This study is a systematic review and did not involve human participants.

Author contributions: MJ, AMAS, TB and MH contributed to the design and conception of the study. MJ drafted the manuscript with feedback from co-authors. MJ, AMAS and RR contributed to the data analysis. NA contributed to developing the search strategy. LC, RA, and NAY were involved in study selection process and in data extraction. MD, SB, JM, PT, BHR, AC, WS brought content expertise in emergency medicine. RS, GH, TB, and JE brought content expertise in family medicine. CS brought content expertise in patient engagement. ACT, RZ, MJ, and AMAS brought content expertise in systematic review methodology. All study authors approved the final manuscript.

For peer review only

Abbreviations:

ATS: Australian triage scale

COPD: Chronic obstructive pulmonary disease

CTAS: Canadian triage and acuity scale

CBA: Controlled before and after

ED: Emergency department

ESI: Emergency Severity Index

GP: General practitioner

LAMA: Leaving against medical advice

LOS: Length of stay

LWBS: Leaving without being seen

NICE: National Institute for Health and Care Excellence

NP: Nurse practitioner

PHCP: Primary healthcare provider

PRISMA: Preferred Reporting Items for Systematic reviews and Meta-Analyses

RCT: Randomized controlled trial

TT: Time to triage

PIA: Time to provider initial assessment

For peer review only

For peer review only - http://bmjopen.bmj.com/site/about/guidelines.xhtml

References

1. Hsia RY, Kellermann AL, Shen Y-C. Factors Associated With Closures of Emergency Departments in the United States. *JAMA.* 2011;305(19):1978-1985.
2. Pitts SR, Pines JM, Handrigan MT, Kellermann AL. National trends in emergency department occupancy, 2001 to 2008: effect of inpatient admissions versus emergency department practice intensity. *Annals of emergency medicine.* 2012;60(6):679-686. e673.
3. Schull MJ, Szalai JP, Schwartz B, Redelmeier DA. Emergency department overcrowding following systematic hospital restructuring trends at twenty hospitals over ten years. *Academic Emergency Medicine.* 2001;8(11):1037-1043.
4. Skinner H, Blanchard J, Elixhauser A. Trends in emergency department visits, 2006–2011: Statistical brief# 179. 2006.
5. Trzeciak S, Rivers EP. Emergency department overcrowding in the United States: an emerging threat to patient safety and public health. *Emergency Medicine Journal.* 2003;20(5):402-405.
6. Pines JM, Hollander JE. Emergency department crowding is associated with poor care for patients with severe pain. *Annals of emergency medicine.* 2008;51(1):1-5.
7. Richardson DB. Increase in patient mortality at 10 days associated with emergency department overcrowding. *Medical journal of Australia.* 2006;184(5):213-216.
8. Oredsson S, Jonsson H, Rognes J, et al. A systematic review of triage-related interventions to improve patient flow in emergency departments. *Scandinavian journal of trauma, resuscitation and emergency medicine.* 2011;19(1):43.
9. Morley C, Unwin M, Peterson GM, Stankovich J, Kinsman L. Emergency department crowding: A systematic review of causes, consequences and solutions. *PloS one.* 2018;13(8):e0203316.
10. Asplin BR, Magid DJ, Rhodes KV, Solberg LI, Lurie N, Camargo CA, Jr. A conceptual model of emergency department crowding. *Ann Emerg Med.* 2003;42(2):173-180.
11. Fatovich DM, Hirsch RL. Entry overload, emergency department overcrowding, and ambulance bypass. *Emerg Med J.* 2003;20(5):406-409.
12. Platter MEM, Kurvers RAJ, Janssen L, Verweij MMJ, Barten DG. The impact of an emergency care access point on pediatric attendances at the emergency department: An observational study. *The American journal of emergency medicine.* 2019.
13. Primary Care Provider. https://www.healthcare.gov/glossary/primary-care-provider/.
14. Uscher-Pines L, Pines J, Kellermann A, Gillen E, Mehrotra A. Deciding to visit the emergency department for non-urgent conditions: a systematic review of the literature. *The American journal of managed care.* 2013;19(1):47.
15. Morgan SR, Smith MA, Pitts SR, et al. Measuring value for low-acuity care across settings. *Am J Manag Care.* 2012;18(9):e356-363.
16. Sancton K, Sloss L, Berkowitz J, Strydom N, McCracken R. Low-acuity presentations to the emergency department: Reasons for and access to other health care providers before presentation. *Can Fam Physician.* 2018;64(8):e354-e360.
17. Uscher-Pines L, Pines J, Kellermann A, Gillen E, Mehrotra A. Emergency department visits for nonurgent conditions: systematic literature review. *Am J Manag Care.* 2013;19(1):47-59.
18. Hayden C, Burlingame P, Thompson H, Sabol VK. Improving patient flow in the emergency department by placing a family nurse practitioner in triage: a quality-improvement project. *Journal of Emergency Nursing.* 2014;40(4):346-351.
19. Tsai VW, Sharieff GQ, Kanegaye JT, Carlson LA, Harley J. Rapid medical assessment: improving pediatric emergency department time to provider, length of stay, and left without being seen rates. *Pediatric emergency care.* 2012;28(4):354-356.

For peer review only - http://bmjopen.bmj.com/site/about/guidelines.xhtml

20. Uthman OA, Walker C, Lahiri S, et al. General practitioners providing non-urgent care in emergency department: a natural experiment. *BMJ Open.* 2018;8(5):e019736.
21. Mortensen K. Copayments did not reduce medicaid enrollees' nonemergency use of emergency departments. *Health Aff (Millwood).* 2010;29(9):1643-1650.
22. Ismail SA, Gibbons DC, Gnani S. Reducing inappropriate accident and emergency department attendances:: a systematic review of primary care service interventions. *Br J Gen Pract.* 2013;63(617):e813-e820.
23. Kirkland SW, Soleimani A, Rowe BH, Newton AS. A systematic review examining the impact of redirecting low-acuity patients seeking emergency department care: is the juice worth the squeeze? *Emerg Med J.* 2019;36(2):97-106.
24. Rose KD, Ross JS, Horwitz LI. Advanced access scheduling outcomes: a systematic review. *Arch Intern Med.* 2011;171(13):1150-1159.
25. Degani N. Impact of advanced (open) access scheduling on patients with chronic diseases: an evidence-based analysis. Ont Health Technol Assess Ser [Internet]. 2013 September;13(7):1–48. Available from: https://www.hqontario.ca/Portals/0/Documents/evidence/reports/full-report-advanced-access-130911-en.pdf.
26. Love RA, Murphy JA, Lietz TE, Jordan KS. The effectiveness of a provider in triage in the emergency department: a quality improvement initiative to improve patient flow. *Advanced Emergency Nursing Journal.* 2012;34(1):65-74.
27. Rowe BH, Guo X, Villa-Roel C, et al. The role of triage liaison physicians on mitigating overcrowding in emergency departments: a systematic review. *Academic Emergency Medicine.* 2011;18(2):111-120.
28. Cheung WWH, Heeney L, Pound JL. An advance triage system. *Accident & Emergency Nursing.* 2002;10(1):10-16.
29. Edwards T. How rapid assessment at triage can improve care outcomes. *Emergency Nurse.* 2011;19(6):27-30.
30. Nash K, Nguyen H, Tillman M. Using medical screening examinations to reduce emergency department overcrowding. *Journal of Emergency Nursing.* 2009;35(2):109-113.
31. Organ K, Chinnick P, Higgison I, Stanhope B, Hoskins R, Benger J. Evaluating the introduction of a paediatric emergency nurse practitioner service. *Emergency nurse : the journal of the RCN Accident and Emergency Nursing Association.* 2005;13(7):8-11.
32. Rogers T, Ross N, Spooner D. Evaluation of a 'see and treat' pilot study introduced to an emergency department. *Accident and Emergency Nursing.* 2004;12(1):24-27.
33. Shrimpling M. Redesigning triage to reduce waiting times. *Emerg Nurse.* 2002;10(2):34-37.
34. Liberati A, Altman DG, Tetzlaff J, et al. The PRISMA statement for reporting systematic reviews and meta-analyses of studies that evaluate health care interventions: explanation and elaboration. *PLoS medicine.* 2009;6(7):e1000100.
35. McGowan J, Sampson M, Salzwedel D, Cogo E, Foerster V, Lefebvre C. PRESS Peer Review of Electronic Search Strategies: 2015 Guideline Statement. *Journal of clinical epidemiology.*75:40-46.
36. Appendix F Quality appraisal checklist-quantitative intervention studies. 2012; https://www.nice.org.uk/process/pmg4/chapter/appendix-f-quality-appraisal-checklist-quantitative-intervention-studies. Accessed July 24, 2019, 2019.
37. Staniszewska S, Brett J, Simera I, et al. GRIPP2 reporting checklists: tools to improve reporting of patient and public involvement in research. *BMJ.* 2017;358:j3453.
38. Adam H, Tamim H, Altamimi S, et al. Effect of triage nurse ordered distal extremity X-rays on emergency department length of stay: A randomized controlled trial. *Academic Emergency Medicine.* 2014;21(5 SUPPL. 1):S195.

For peer review only - http://bmjopen.bmj.com/site/about/guidelines.xhtml

39. Al Abri FH, Muliira JK, Al Awaisi H. Effect of triage nurse-led application of the Ottawa Ankle Rules on number of radiographic tests and length of stay in selected emergency departments in Oman. *Japan journal of nursing science : JJNS.* 2020;17(1):e12270.
40. Al Kadhi O, Manley K, Natarajan M, et al. A renal colic fast track pathway to improve waiting times and outcomes for patients presenting to the emergency department. *Open access emergency medicine : OAEM.* 2017;9:53-55.
41. Ashurst JV, Nappe T, Digiambattista S, et al. Effect of triage-based use of the Ottawa foot and ankle rules on the number of orders for radiographic imaging. *The Journal of the American Osteopathic Association.* 2014;114(12):890-897.
42. Celona CA, Amaranto A, Ferrer R, et al. Interdisciplinary Design to Improve Fast Track in the Emergency Department. *Advanced Emergency Nursing Journal.* 2018;40(3):198-203.
43. Day TE, Al-Roubaie AR, Goldlust EJ. Decreased length of stay after addition of healthcare provider in emergency department triage: a comparison between computer-simulated and real-world interventions. *Emerg Med J.* 2013;30(2):134-138.
44. Demarco F, Gerardo CJ, Boardwine A, et al. Effect of a nurse rapid intake initiative on patient length of stay and satisfaction: A project IMPACT initiative. *Academic Emergency Medicine.* 2010;17(SUPPL. 1):S93-S94.
45. Dixon A, Clarkin C, Barrowman N, Correll R, Osmond MH, Plint AC. Reduction of radial-head subluxation in children by triage nurses in the emergency department: A cluster-randomized controlled trial. *CMAJ.* 2014;186(9):E317-E323.
46. Fan J, Woolfrey K. The effect of triage-applied Ottawa Ankle Rules on the length of stay in a Canadian urgent care department: a randomized controlled trial. *Academic emergency medicine : official journal of the Society for Academic Emergency Medicine.* 2006;13(2):153-157.
47. Fontanel A, Besson C, Gallegos C, Vallot C, Droal D. Can X-rays be prescribed by a nurse in an emergency department? *European Journal of Emergency Medicine.* 2011;18(5):310-311.
48. Gardner RM, Friedman NA, Carlson M, Bradham TS, Barrett TW. Impact of revised triage to improve throughput in an ED with limited traditional fast track population. *American Journal of Emergency Medicine.* 2018;36(1):124-127.
49. Gaucher N, Bailey B, Gravel J. Triage nurses' counselling influences return visits of children leaving the emergency department before being seen by a physician. *Academic Emergency Medicine.* 2010;17(SUPPL. 1):S118-S119.
50. Hackman JL, Roth ED, Gaddis ML, Gratton MC. The effect of a nurse-initiated chest pain protocol on disposition time: A retrospective review. *Annals of Emergency Medicine.* 2015;66(4 SUPPL. 1):S9-S10.
51. Ho JK-M, Chau JP-C, Chan JT-S, Yau CH-Y. Nurse-initiated radiographic-test protocol for ankle injuries: A randomized controlled trial. *International emergency nursing.* 2018;41:1-6.
52. Jobé J, Vandercleyen C, Ghuysen A, D'Orio V. Prospective study of an advanced nurse triage for a target pathology at the admission in the emergency department. *Acta clinica belgica.* 2013;68(6):482-.
53. Klassen TP, Ropp LJ, Sutcliffe T, et al. A randomized, controlled trial of radiograph ordering for extremity trauma in a pediatric emergency department. *Annals of emergency medicine.* 1993;22(10):1524-1529.
54. Kool RB, Homberg DJ, Kamphuis HC. Towards integration of general practitioner posts and accident and emergency departments: a case study of two integrated emergency posts in the Netherlands. *BMC Health Services Research.* 2008;8:225.
55. Lee KM, Wong TW, Chan R, Lau CC, Fu YK, Fung KH. Accuracy and efficiency of X-ray requests initiated by triage nurses in an accident and emergency department. *Accident and emergency nursing.* 1996;4(4):179-181.

For peer review only - http://bmjopen.bmj.com/site/about/guidelines.xhtml

56. Lee WW, Filiatrault L, Abu-Laban RB, Rashidi A, Yau L, Liu N. Effect of triage nurse initiated radiography using the Ottawa Ankle Rules on emergency department length of stay at a tertiary care center. *Canadian Journal of Emergency Medicine.* 2014;16(SUPPL. 1):S38.
57. Lee WW, Filiatrault L, Abu-Laban RB, Rashidi A, Yau L, Liu N. Effect of Triage Nurse Initiated Radiography Using the Ottawa Ankle Rules on Emergency Department Length of Stay at a Tertiary Centre. *CJEM.* 2016;18(2):90-97.
58. Li Y, Lu Q, Du H, Zhang J, Zhang L. The Impact of Triage Nurse-ordered Diagnostic Studies on Pediatric Emergency Department Length of Stay. *Indian journal of pediatrics.* 2018;85(10):849-854.
59. Lijuan Z. Advanced Triage Protocols in the Emergency Department. *Advanced Triage Protocols in the Emergency Department.* 2017:1-1.
60. Lindley-Jones M, Finlayson BJ. Triage nurse requested x rays--are they worthwhile? *J Accid Emerg Med.* 2000;17(2):103-107.
61. MacKenzie RS, Burmeister DB, Brown JA, et al. Implementation of a rapid assessment unit (intake team): impact on ED length of stay. *American Journal of Emergency Medicine.* 2015;33(2):291-293.
62. Parris W, McCarthy S, Kelly AM, Richardson S. Do triage nurse-initiated X-rays for limb injuries reduce patient transit time? *Accid Emerg Nurs.* 1997;5(1):14-15.
63. Pierce BA, Gormley D. Are Split Flow and Provider in Triage Models in the Emergency Department Effective in Reducing Discharge Length of Stay? *Journal of Emergency Nursing.* 2016;42(6):487-491.
64. Sikkenga T, Dumkow L, Draper H, et al. Implementation of a nursing triage order to improve utilization of rapid diagnostic testing for chlamydia and gonorrhea in the emergency department. *Open Forum Infectious Diseases.* 2016;3(Supplement 1).
65. Thurston J, Field S. Should accident and emergency nurses request radiographs? Results of a multicentre evaluation. *J Accid Emerg Med.* 1996;13(2):86-89.
66. Tsai VW, Sharieff GQ, Kanegaye JT, Carlson LA, Harley J. Rapid medical assessment: improving pediatric emergency department time to provider, length of stay, and left without being seen rates. *Pediatric Emergency Care.* 2012;28(4):354-356.
67. Tucker A, Bernard M. Making the Case for Nurse Practitioners in the Emergency Department: A Clinical Case Study. *Advanced Emergency Nursing Journal.* 2015;37(4):308-312.
68. van den Bersselaar DLCM, Maas M, Thijssen WAMH. Does X-ray imaging by GPC at emergency care access points in the Netherlands change patient flow and reduce ED crowding? A cohort study. *Health science reports.* 2018;1(2):e26.
69. van Gils-van Rooij ESJ, Meijboom BR, Broekman SM, Yzermans CJ, de Bakker DH. Is patient flow more efficient in Urgent Care Collaborations? *European Journal of Emergency Medicine.* 2018;25(1):58-64.
70. Zager K, Taylor YJ. Discharge to medical home: A new care delivery model to treat non-urgent cases in a rural emergency department. *Healthcare.* 2018;21:21.
71. MacKenzie RS, Burmeister DB, Brown JA, et al. Implementation of a rapid assessment unit (intake team): impact on ED length of stay. *Am J Emerg Med.* 2015;33(2):291-293.
72. Rowe BH, Villa-Roel C, Guo X, et al. The role of triage nurse ordering on mitigating overcrowding in emergency departments: a systematic review. *Acad Emerg Med.* 2011;18(12):1349-1357.
73. Jennings N, Clifford S, Fox AR, O'Connell J, Gardner G. The impact of nurse practitioner services on cost, quality of care, satisfaction and waiting times in the emergency department: a systematic review. *International journal of nursing studies.* 2015;52(1):421-435.

For peer review only - http://bmjopen.bmj.com/site/about/guidelines.xhtml

74. Gonçalves-Bradley D, Khangura JK, Flodgren G, Perera R, Rowe BH, Shepperd S. Primary care professionals providing non-urgent care in hospital emergency departments. *Cochrane Database of Systematic Reviews.* 2018(2).

For peer review only

For peer review only - http://bmjopen.bmj.com/site/about/guidelines.xhtml

Table 1: Characteristics of included studies

Study ID (First Author, Year)	Country; Urban/rural ED; Adult/Pediatric/mixed ED	Study Design	Type of PHCP	Intervention	Duration of intervention (months)	Study Quality
Adam, 2014	Saudi Arabia; Urban ED; Pediatric ED	RCT	Nurse	Nurse triage-plus	0.5	Moderate
Al Abri, 2020	Oman; Urban ED; Adult ED	CBA	Nurse	Nurse triage-plus	NR	Low
Al Khadi, 2017	UK; NR; NR	RC study	Nurse	Nurse triage-plus	12	Low
Ashurst, 2014	USA; Urban ED; Mixed ED	PC study	Nurse	Nurse triage-plus	10	Low
Celona, 2018	USA; Urban ED; Adult ED	Pre-post	NP	NP Team triage	12	Low
Cheung, 2002	Canada; Urban ED; NR	RC study	Nurse	Nurse triage-plus	NR	Low
Day, 2013	USA; Urban ED; NR	Pre-post	NP	NP Team triage	1	Low
Demarco, 2010	NR; Urban ED; NR	PC study	Nurse	Nurse triage-plus	1	Low
Dixon, 2014	Canada; Urban ED; Pediatric ED	RCT	Nurse	Nurse triage-plus	12	Low
Edwards, 2011	Australia; Urban ED; Mixed ED	Pre-post	NP	NP Team triage	17	Low
Fan, 2006	Canada; Urban ED; NR	RCT	Nurse	Nurse triage-plus	3	Moderate
Fontanel, 2011	France; NR; Mixed ED	CBA	Nurse	Nurse triage-plus	0.08	Low
Gardner, 2018	USA; Urban ED; Adult ED	Pre-post	NP	NP Team triage	0.5	Low
Gaucher, 2010	Canada; NR; Pediatric ED	RC study	Nurse	Nurse triage-plus	12	Low
Hackman, 2015	USA; Urban ED; NR	RC study	Nurse	Nurse triage-plus	17	Low
Hayden, 2014	USA; NR; Mixed ED	Pre-post	NP	NP Team triage	2	Low
Ho, 2018	China; NR; Mixed ED	RCT	Nurse	Nurse triage-plus	NR	Moderate
Jobe, 2019	France; NR; NR	RCT	Nurse	Nurse triage-plus	NR	Low
Klassen, 1993	Canada; Urban ED; Pediatric ED	RCT	Nurse	Nurse triage-plus	12	Low
Kool, 2008	Netherlands; Both; NR	CBA	GP	GP team triage	12	Low
Lee, 1996	China; Urban ED; Mixed ED	PC study	Nurse	Nurse triage-plus	3	Low
Lee, 2014	Canada, Urban ED; NR	RCT	Nurse	Nurse triage-plus	12	Moderate
Lee, 2016	Canada; Urban ED; Adult ED	RCT	Nurse	Nurse triage-plus	12	Moderate
Li, 2018	China; NR; Pediatric ED	RC study	Nurse	Nurse triage-plus	5	Low
Lijuan, 2017	USA; NR; NR	Pre-post	Nurse	Nurse triage-plus	6	Low
Lindley Jones, 2000	England; Urban ED; NR	RCT	Nurse	Nurse triage-plus	12	Moderate

For peer review only - http://bmjopen.bmj.com/site/about/guidelines.xhtml

Love, 2012	USA; Urban ED; NR	Pre-post	NP	NP Team triage	0.5	Low
MacKenzie, 2015	USA; NR; NR	Pre-post	NP	NP Team triage	2	Low
Parris, 1997	Australia; Urban ED; Adult ED	Quasi-RCT	Nurse	Nurse triage-plus	6	Low
Pierce, 2016	USA; Urban ED; NR	CBA	NP	NP Team triage	5.5	Low
Rogers, 2004	England; Urban ED; NR	Pre-post	NP	NP Team triage	12	Low
Shrimpling, 2002	England; Urban ED; NR	Pre-post	NP	NP Team triage	0.75	Low
Sikkenga, 2016	USA; Urban ED; NR	RC study	Nurse	Nurse triage-plus	NR	Low
Thurston, 1996	England; Urban ED; NR	RCT	Nurse	Nurse triage-plus	2	Moderate
Tsai, 2012	USA; Urban ED; Pediatric ED	Pre-post	NP	NP Team triage	NR	Low
Tucker, 2015	USA; Urban ED; NR	Pre-post	NP	NP Team triage	6	Low
Uthman, 2018	England; Urban ED; NR	RC study	NP	NP Team triage	12	Low
van den Bersselaar, 2018	Netherlands; Urban ED; NR	RC study	GP	GP team triage	11	Low
van Gils-van Rooij, 2018	Netherlands; Both; NR	CS study	GP	GP team triage	NA	Low
Zager, 2018	USA; Rural ED; NR	Pre-post	NP	NP Team triage	4	Low

CS study: Cross-sectional observational study; RC study: Retrospective cohort study; ED: Emergency department; PHCP: Primary healthcare provider; CBA: Controlled before and after study; RCT: Randomized controlled trial; GP: General practitioner; NP: Nurse practitioner; NR: Not reported; NA: Not applicable

For peer review only

For peer review only - http://bmjopen.bmj.com/site/about/guidelines.xhtml

Table 2: Leave without being seen (LWBS) outcome data reported by included studies

Study ID (First Author, Year)	Triage Intervention	Study Design	Intervention (%)	Comparator (%)	Percentage Difference	Reported Statistical Significance
Celona, 2018	NP team triage	Pre-post	4.7	3.3	1.4	NR
Love, 2012	NP team triage	Pre-post	0.93	3.39	-2.46	Significant
MacKenzie, 2015	NP team triage	Pre-post	0.7333	2.96	-2.2267	Significant
Gardner, 2017	NP team triage	Pre-post	2.2	4.6	-2.4	Significant
Hayden, 2014	NP team triage	Pre-post	5.8	5.4	0.4	NS
Tsai, 2012	NP team triage	Pre-post	3	9.7	-6.7	Significant
Tucker, 2015	NP team triage	Pre-post	1.3	5.07	-3.77	NR
Uthman, 2018	NP team triage	Retrospective cohort	2.2	3.9	-1.7	Significant
Li, 2018	Nurse triage-plus	Retrospective cohort	0.7	6.9	-6.2	NS
Lijuan, 2017	Nurse triage-plus	Pre-post	7.13	7.52	-0.39	NS

NP: Nurse practitioner; NR: Not reported; NS: Not significant

For peer review only

For peer review only - http://bmjopen.bmj.com/site/about/guidelines.xhtml

Table 3: Leave Against Medical Advice (LAMA) outcome data reported by included studies

Study ID (First Author, Year)	Triage Intervention	Study Design	Intervention (%)	Comparator (%)	Percentage Difference	Reported Statistical Significance
MacKenzie, 2015	NP team triage	Pre-post	0.22	0.33	-0.11	NS
Tucker, 2015	NP team triage	Pre-post	1.41	1.29	0.12	NS
Hayden, 2014	NP team triage	Pre-post	1.4	0.06	1.34	NR

NP: Nurse practitioner; NR: Not reported; NS: Not significant

For peer review only

For peer review only - http://bmjopen.bmj.com/site/about/guidelines.xhtml

Figure legends

Figure 1: PRISMA study flow diagram

Figure 2: Various models for PHCP involvement in triage of emergency department patients.

Figure 3: Effectiveness of PHCP interventions on time to provider initial assessment (in minutes) sub-grouped by study design. The horizontal black lines represent 95% confidence intervals and the red dots in the middle represents point estimates (mean difference).

Figure 4: Effectiveness of PHCP interventions on ED LOS (in minutes) sub-grouped by study design. The horizontal black lines represent 95% confidence intervals and the red dots in the middle represents point estimates (mean difference).

For peer review only

For peer review only - http://bmjopen.bmj.com/site/about/guidelines.xhtml

Figure 1: PRISMA study flow diagram

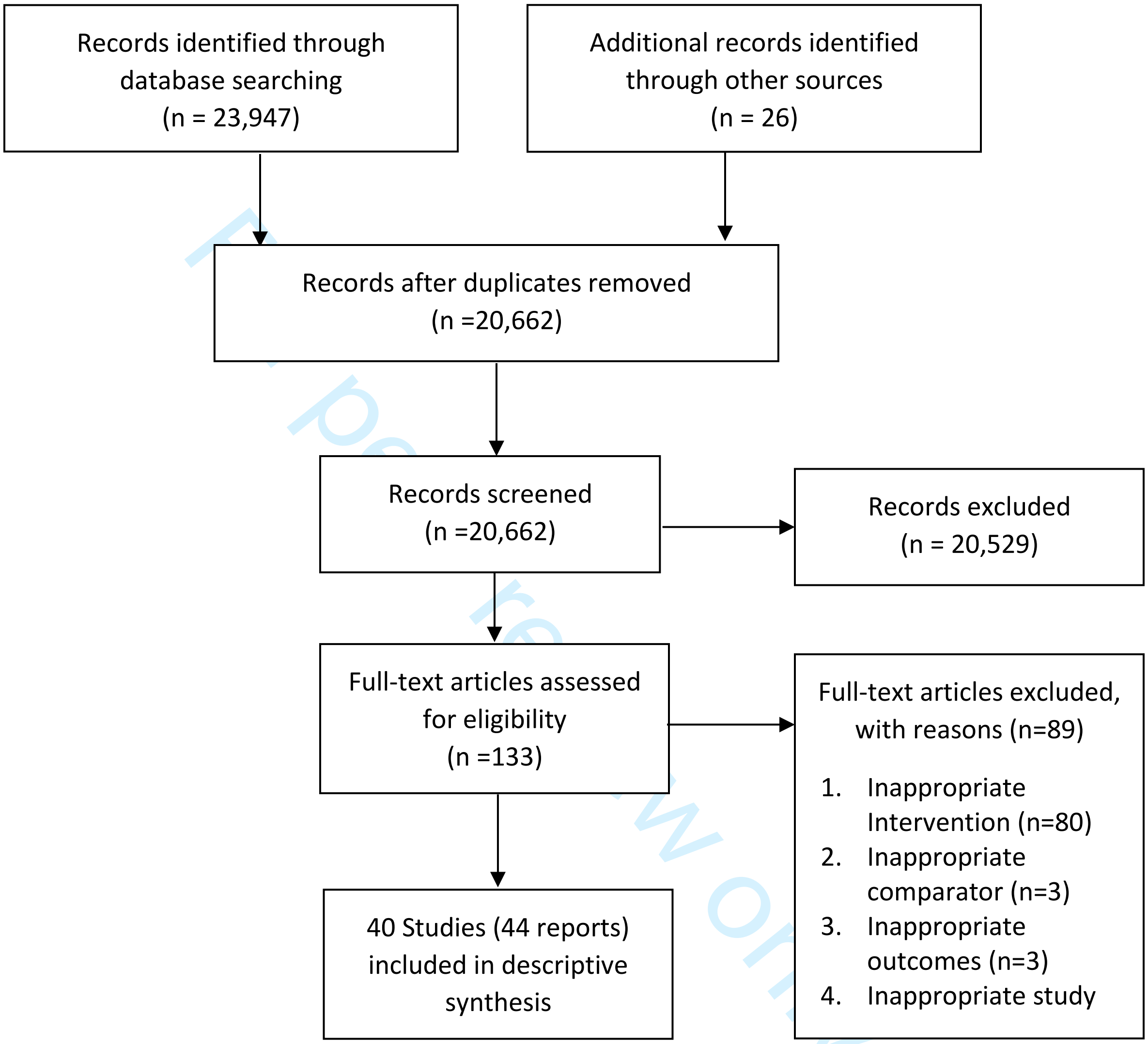

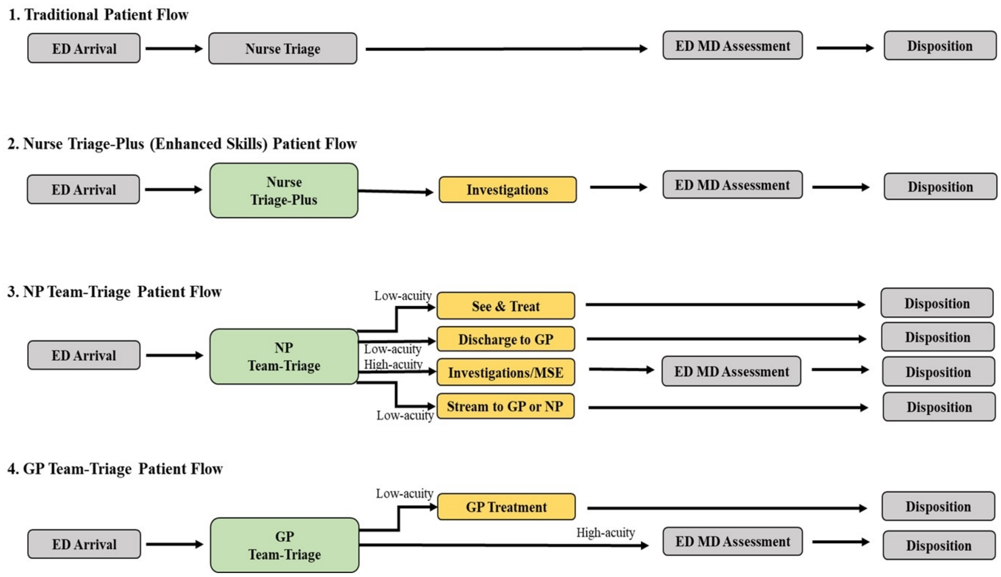

Various models for PHCP involvement in triage of emergency department patients

228x131mm (150 x 150 DPI)

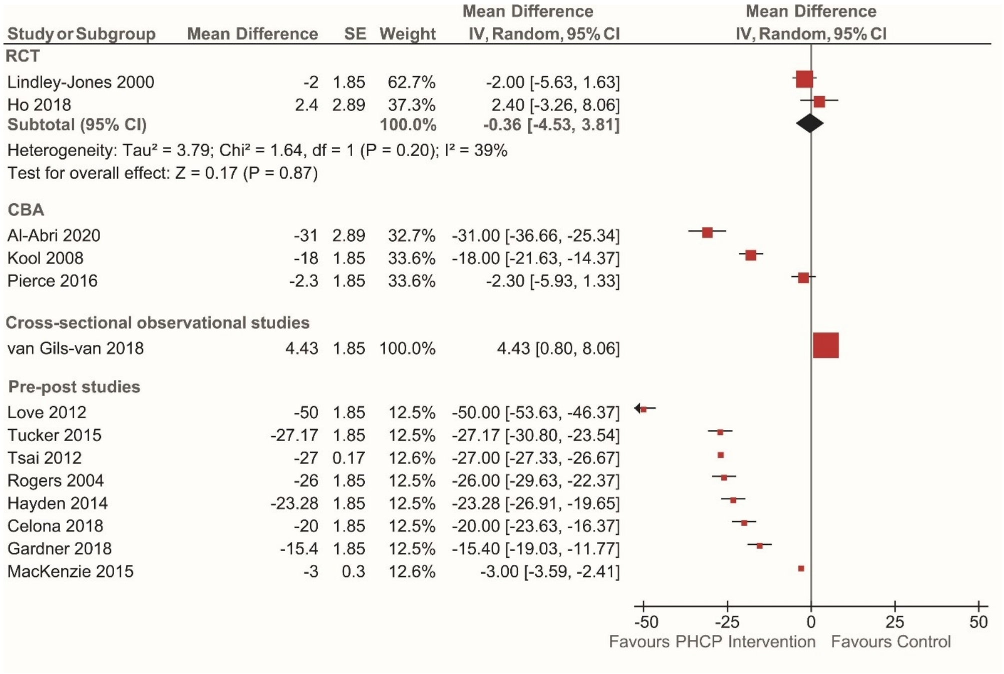

Effectiveness of PHCP interventions on time to provider initial assessment (in minutes) sub-grouped by study design

161x108mm (220 x 220 DPI)

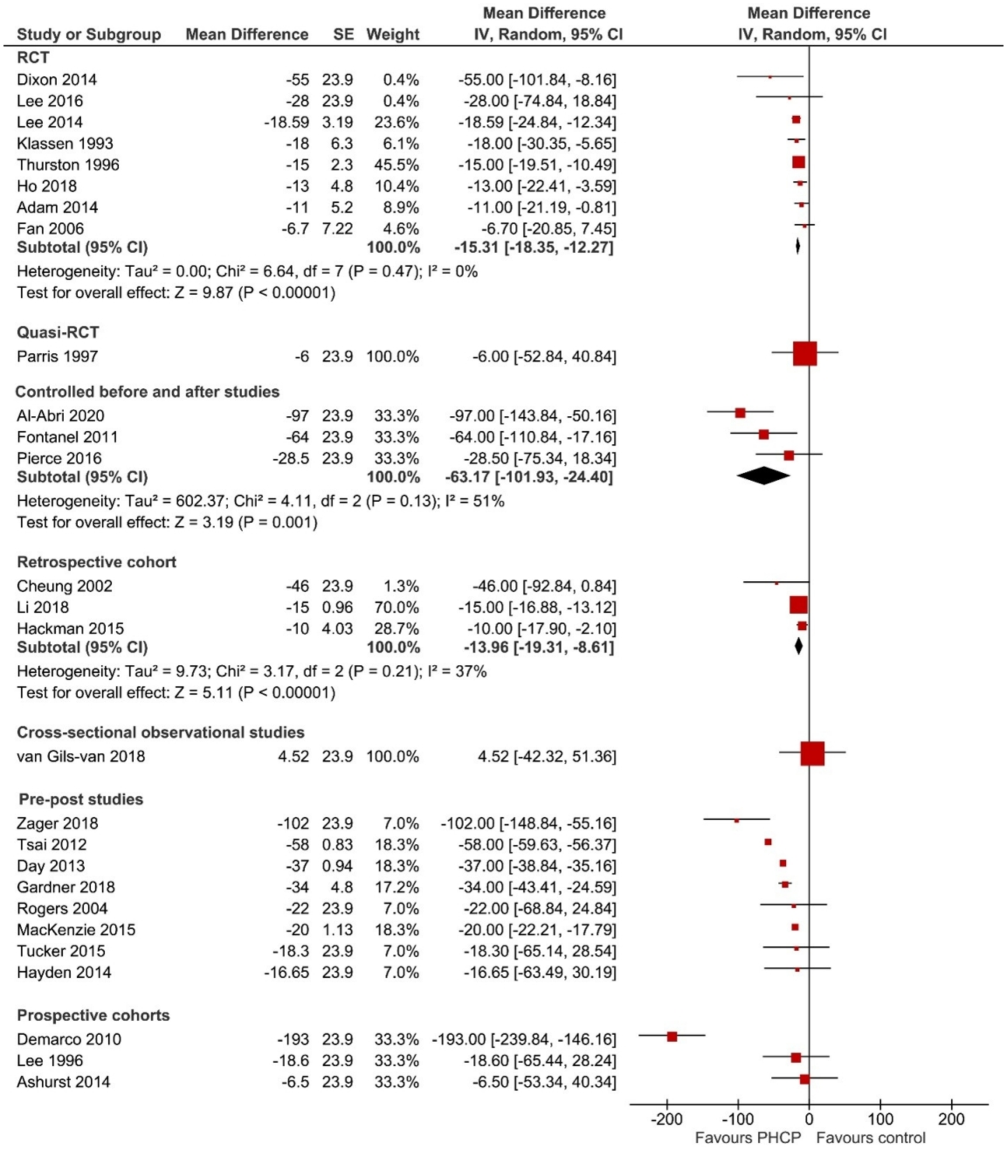

Effectiveness of PHCP interventions on ED LOS (in minutes) sub-grouped by study design

165x189mm (220 x 220 DPI)

Appendix Methods:

The National Institute for Health and Care Excellence (NICE) quality appraisal tool has four domains: characteristics of the population, allocation methods, outcomes, and analyses. Each domain has multiple questions for which there are five response options: (1) study has been conducted in such a way to minimize the risk of bias (++), (2) study has not addressed all potential sources of bias (+), (3) significant sources of bias persists in the study (-), (4) not reported or (5) not applicable. A fifth domain summarizes the overall quality of the included study based on the assessments of the four domains. The overall quality of each included study was assessed as either low quality (-), moderate quality (+) or high quality (++), based on adjudications made on the four individual domains for that study.

Appendix Results:

1. ***Patient triage acuity rating***: Various scales such as Emergency Severity Index (ESI), Australasian Triage Scale (ATS), the Canadian Triage or Acuity Scale (CTAS) or triage acuity rating scale were used to assess the acuity levels patients arriving at the ED. Six included studies[1-6] (15%) reported triaging patients who were triage category 3-5 (one study involved <10% of category 3 patients, two studies[2,7] involved >20% of category 3 patients, and three studies[1,4,5] did not report the percentage of patients in each category 3-5). Five included studies[8-12] (10%), reported triaging both low and high acuity patients (with approximately 90% of category 3-5 patients). Fourteen (35%) studies[13-26] did not report acuity levels of patients.

2. ***Time to physician initial assessment (PIA) sub-grouped by various PHCP interventions:***

   Of the 14 studies, the majority[18,26,32,42,48,61,63,66,67] (n = 9) reported the effect of NP team triage on PIA, and the rest reported either the effect of GP team triage[54,69] (n = 2) or nurse

For peer review only

triage-plus[39,51,60] (n=3) on PIA, respectively. All studies in NP team triage group showed a decrease in PIA (median [range]= -21.7 minutes [-2.3 to -50]) favoring the intervention group. In the nurse triage-plus group all except one[51] showed a decrease in PIA (median [range]= -2.4 minutes [-2 to -31]), Among the two studies[54,69] in GP team triage group, one prospective CBA interventional study[54] reported statistically significant decrease (-18 minutes) in PIA favoring the intervention group. Whereas the second cross-sectional observational study[69] showed an increase (4.43 minutes) in PIA (reported as statistically significant), favoring the traditional nurse-led triage model.

3. *Emergency department length of stay (ED LOS) sub-grouped by various PHCP interventions:*

Twenty studies[7,8,13-19,21-31] reported on the effect of nurse triage-plus on ED LOS, nine studies[1,2,4-6,10,32-34] reported on the effect of NP team triage on ED LOS, and one study[11] on the effect of GP team triage on ED LOS. Seventeen studies[7,8,13,15-19,21,23-25,27-31] in the nurse triage-plus model reported a decrease (median = -18 minutes) in ED LOS favoring the intervention group. All nine studies[1,2,4-6,10,32-34] in the NP team triage model showed a decrease (median = -28.50 minutes) in ED LOS favoring the intervention group. One study in the GP team triage model did not show any significant difference in ED LOS between comparison groups.

Four studies reported percentage of patients discharged within benchmark times (ED specific). Rogers et al. reported 41% of patients discharged from the ED within one hour in the NP team triage group compared to only 16% patients discharged within one hour in the traditional nurse-led triage group. Tsai et al.[35] reported that 30% of low-acuity patients in the NP team triage group discharged in 90 minutes compared to 12% in the

For peer review only

traditional nurse-led triage group. Day et al.[1] reported that 85.7% of patients discharged under 6 hours in the NP team triage group compared to 80.1% in the traditional nurse-led triage group. Uthman et al.[36] reported that 98.1% of patients discharged under 4 hours in the in the NP team triage group compared to 94.7% in the traditional nurse-led triage group.

4. ***Effect of PHCP intervention on number of repeat ED visits***

Zager et al.6 reported a 5% decrease in ED visits in the NP team triage group (conducted triage, medical screening exam (MSE) and discharged low-acuity patients with a same day appointment at the GP clinic co-located with the ED) compared to the traditional nurse-led triage model (statistical significance not reported). Day et al.1 investigating NP team triage (provider at triage model) reported 2194 ED visits (over 6 weeks) during pre-intervention period compared to 1699 patient visits (over one month) during the post-intervention period (statistical significance not reported). Tucker et al.34 investigated the effect of NP team triage on ED visits and reported an increase in the number of patients visiting ED by 51 visits per month (statistical significance not reported) compared to the traditional nurse-led triage model. Bersselaar et al.37 investigated the effect of GP team triage and x-ray requests (at the emergency care access point (ECAP) in which ED and GP work together) on ED visits, and reported that 68% of patient visits were treated by the GP without ED referral leading to a reduction of 4.5% annual ED patient visits. Kool et al.38, a CBA study, investigated the effect of GP team triage at the integrated emergency post (IEP) with a joint reception for the ED and a GP clinic on ED visits compared to the control sites that are not IEP (traditional nurse-led triage model), and reported a statistically significant decrease (6257 to 5715) in the number of patient visits

For peer review only - http://bmjopen.bmj.com/site/about/guidelines.xhtml

at the ED at IEPs and an statistically significant increase (3985 to 4321) in the number of ED attendances at the control sites. Gaucher et al20 reported that number of return ED visits decreased from 8.1% to 6.1% in the nurse triage-plus group compared to the traditional nurse-led triage model.

5. ***Effect of PHCP intervention on patient satisfaction***

Kool et al.[37] reported no differences in patient satisfaction between patients who visited IEPs (GP team triage) compared to those who visited ED's at control sites, but patients who were phone triaged at the IEP were more satisfied (statistically significant) compared to the control sites EDs[37]. Tucker et al.[34] investigated the effect of NP team triage on ED visits and reported that patient satisfaction remained high (greater than 90%; statistical significance not reported) compared to the traditional nurse-led triage model. Gardner et al.[32] reported that with NP team triage, 62-65% of patients were more satisfied with their ED LOS, PIA and quality of care compared to traditional nurse-led triage model. Hayden et al.[2] investigated the impact of NP team triage (provider at triage model) on patient satisfaction and reported that patient satisfaction decreased slightly in the post-intervention period compared to the pre-intervention period but this decrease was not statistically significant. Five[7,12,15,16,25] studies reported an increase in patient satisfaction scores in the nurse triage-plus model compared to the traditional nurse-led triage model, whereas one[18] study reported no difference between groups.

6. ***Effect of PHCP intervention on time to triage***

One RCT[28] showed a non-significant decrease in time to triage in the nurse triage-plus group compared to traditional nurse-led triage group. Two pre-post studies[33,38] reported the effect of NP team triage on time to triage compared to the traditional nurse-led triage

For peer review only - http://bmjopen.bmj.com/site/about/guidelines.xhtml

model. MacKenzie et al.[38] reported statistically significant decrease (pre-intervention time to triage (Median: 4; IQR: (2, 10)); post-intervention time to triage (Median: 3; IQR: (1, 8)) favoring the intervention. Rogers et al.[33] reported that 98% percentage of patients in the NP team triage intervention group were triaged within 15 minutes compared to the comparison group (75% of patients triaged within 15 minutes).

For peer review only

Appendix Table 1: Inclusion and exclusion criteria

	**Inclusion criteria**	**Exclusion criteria**
Population	Patient population (children and adults of any age) visiting the ED	
Intervention	Any ED triage intervention or strategy involving primary healthcare providers (family physicians/general practitioner (GP), nurse practitioner (NP), or nurse given increased authority)	Studies reporting triage intervention involving emergency physicians (ED MD) or exclusively physician assistants
Comparator	Traditional nurse-led triage (standard care)	
Outcomes	*Primary outcomes*: Time to provider initial assessment	
	Secondary outcomes: ED LOS, proportion of patients that left without being seen (LWBS), ED length of stay patient satisfaction, proportion of patients leaving against medical advice (LAMA), time to triage, and number of ED visits.	
Study Design	Any comparative study design (randomized and quasi-randomized clinical trials, non-randomized controlled clinical trial/controlled before and after studies (CBA), case control studies, controlled cohort studies, interrupted time series, pre-post intervention/uncontrolled before and after studies)	Reviews, commentary, case reports, editorials, historical articles, non-human studies

For peer review only

Appendix Table 2: Medline search strategy

Database: Ovid MEDLINE(R) and Epub Ahead of Print, In-Process & Other Non-Indexed Citations and Daily <1946 to January 10, 2020>
Search Strategy:
----
1 exp primary health care/ (140156)
2 physicians, family/ (15853)
3 family practice/ (64029)
4 Physicians, Primary Care/ (2703)
5 general practice/ (11719)
6 general practitioners/ (6381)
7 (primary adj2 (care or health*)).ti,ab,kf. (129329)
8 ((general or family) adj (practice* or practitioner*)).ti,ab,kf. (84810)
9 (GP or GPs).ti,ab,kf. (51935)
10 nurse practitioners/ (16770)
11 primary care nursing/ (392)
12 family nursing/ (1349)
13 community mental health services/ (17905)
14 ((family or community or primary or ambulatory or triage) adj2 (medic* or doctor* or physician* or health* or nurs*)).ti,ab,kf. (68438)
15 Ambulatory Care/ (40524)
16 (ambulatory adj2 care).ti,ab,kf. (11413)
17 Health Services, Indigenous/ (2817)
18 Cultural Competency/ (4632)
19 Culturally Competent Care/ (830)
20 Medicine, Traditional/ (10299)
21 (trauma adj inform*).ti,ab,kf. (617)
22 (aborigin* or indigenous or native).ti,ab,kf. (225451)
23 ((after or out) adj2 hour*).ti,ab,kf. (137459)
24 or/1-23 (917850)
25 exp Emergency Service, Hospital/ (67418)
26 Emergency Medical Services/ (39232)
27 emergency treatment/ (10025)
28 Trauma centers/ (9210)
29 Triage/ (10240)
30 ((emergency or emergent or urgent) adj2 (care or healthcare or department* or unit or units or room* or treatment* or ward or service)).ti,ab,kf. (121497)
31 ("accident and emergency" or "accident & emergency" or ED or EDs or ER or A&E).ti,ab,kf. (162648)
32 (triage adj2 (centre or centres or center or centers or department? or unit or units)).ti,ab,kf. (538)
33 (emergency adj2 (care or healthcare or department? or unit or units or room? or treatment? or care or visit? or utilization or admit or admission?)).ti,ab,kf. (112731)
34 ("accident and emergency" or "accident & emergency" or emergency service?).ti,ab,kf. (10865)
35 (trauma adj2 (centre or centres or center or centers or department? or unit or units)).ti,ab,kf. (15573)
36 (triage adj2 (centre or centres or center or centers or department? or unit or units)).ti,ab,kf. (538)

For peer review only

37 (emergency adj2 (visit? or care or admit or admission?)).ti,ab,kf. (26760)
38 (urgent adj2 (care or healthcare or health care)).ti,ab,kf. (2099)
39 ((semiurgent or semi-urgent or nonemergen$ or non-emergen$) adj2 (treatment? or care or visit?)).ti,ab,kf. (289)
40 ((emergency or non-emergency or nonemergency or urgent or non-urgent or nonurgent or semi-urgent or semiurgent) adj2 patient?).ti,ab,kf. (11636)
41 or/25-40 (367776)
42 organizational efficiency/ (20744)
43 workflow/ (3295)
44 Waiting lists/ (10724)
45 ((wait or waiting) adj2 (time or times or list or lists)).ti. (3351)
46 ((wait or waiting or throughput or service or treatment) adj2 (time or times or list or lists) adj10 (reduce? or reduction or eliminat$ or lower or fewer or intervention or policy or policies or reform$ or effectiveness or impact or improv$ or organi?ational$ or quality or save or saving)).ab. (3119)
47 ((decrease or reduce or streamline or less or minimize or shorten or eliminate or cut or enhance or facilitate or speed or better or accelerate or optimize or reform or delay or change or faster or impact$ or assess$ or eliminat$ or improv$ or lower$ or reduc$) adj3 patient? wait$).ti,ab,kf. (303)
48 CROWDING/ (2930)
49 crowd$.ti,ab,kf. (16513)
50 congest$.ti,ab,kf. (61747)
51 overcrowd$.ti,ab,kf. (3425)
52 gridlock$.ti,ab,kf. (180)
53 queue$.ti,ab,kf. (1011)
54 overload$.ti,ab. (39413)
55 "access block$".ti,ab,kf. (166)
56 (throughput or through-put).ti,ab,kf. (87262)
57 warehous$.ti,ab,kf. (2303)
58 ("left without being seen" or "leave$ without being seen" or lwbs).ti,ab,kf. (284)
59 (patient adj2 elop$).ti,ab,kf. (16)
60 (ambulance$ adj2 diver$).ti,ab,kf. (194)
61 (ambulance$ adj2 redirect$).ti,ab,kf. (3)
62 "fast track$".ti,ab,kf. (3500)
63 delay$.ti,ab,kf. (428757)
64 ("patient flow$" or "flow of patient$").ti,ab,kf. (4939)
65 defer$.ti,ab,kf. (23198)
66 (over* adj3 (capacit$ or occupanc$)).ti,ab,kf. (4603)
67 (lama or (leave$ adj4 ("medical advice" or treatment$)) or (left adj4 ("medical advice" or treatment$))).ti,ab,kf. (8393)
68 ((hallway or corridor) adj2 (care or medicine)).ti,ab,kf. (6)
69 or/42-68 (776721)
70 24 and 41 and 69 (3799)

For peer review only

Appendix Table 3: Grey literature sources

**Grey literature sources**
BMJ Open Quality (https://bmjopenquality.bmj.com) and a Google Custom Search of the following websites:<br>Canadian Foundation for Healthcare Improvement (www.cfhi-fcass.ca), Institute for Healthcare Improvement (www.ihi.org), Agency for Healthcare Research and Quality (www.ahrq.gov), NHS Improvement (https://improvement.nhs.uk), International Society for Quality in Health Care (www.isqua.org), Health Quality Ontario (www.hqontario.ca), Saskatchewan Health Quality Council (https://hqc.sk.ca), Health Quality Council of Alberta (www.hqca.ca), BC Patient Safety & Quality Council (https://bcpsqc.ca), Australian Commission on Safety and Quality in Health Care (www.safetyandquality.gov.au), and Health Quality & Safety Commission New Zealand (www.hqsc.govt.nz).

For peer review only

Appendix Table 4: Quality assessment scores of included studies

Study ID	1.1	1.2	1.3	2.1	2.2	2.3	2.4	2.5	2.6	2.7	2.8	2.9	3.1	3.2	3.3	3.4	3.5	3.6	4.1	4.2	4.3	4.4	4.5	4.6	5.1	5.2
Celona, 2018	2+	2+	-	-	2+	-	-	2+	2+	NR	-	2+	2+	2+	2+	2+	2+	2+	NR	NR	NR	+	+	-	-	-
Cheung, 2002	2+	2+	NR	-	2+	-	NR	2+	NR	NR	-	-	2+	+	2+	2+	2+	2+	NR	NR	NR	+	+	-	-	-
Day, 2013	2+	2+	-	-	2+	-	-	2+	2+	NR	-	2+	2+	2+	2+	2+	2+	2+	NR	NR	NR	2+	+	2+	-	-
Edwards, 2011	2+	2+	2+	-	2+	-	-	+	2+	NR	-	2+	2+	2+	2+	2+	2+	2+	NR	NR	NR	2+	+	-	-	-
Gardner, 2018	+	2+	+	-	2+	-	-	2+	2+	NR	-	2+	2+	2+	2+	2+	2+	2+	NR	NR	NR	2+	+	2+	-	+
Hayden, 2014	+	+	+	-	2+	-	-	2+	2+	NR	-	2+	2+	2+	2+	2+	2+	2+	+	NR	NR	2+	2+	+	-	+
Lee, 2016	2+	+	2+	2+	2+	2+	+	2+	2+	NR	2+	2+	+	+	2+	2+	2+	2+	+	NR	2+	2+	2+	2+	+	+
Lindley Jones, 2000	2+	2+	2+	2+	2+	+	NR	2+	2+	NR	2+	+	2+	2+	2+	2+	2+	2+	NR	NR	NR	+	+	2+	+	2+
Love, 2012	2+	+	+	-	2+	-	-	2+	2+	NR	-	2+	2+	2+	2+	2+	2+	2+	NR	NR	NR	+	+	-	-	+
Mackenzie, 2015	2+	2+	2+	-	2+	-	-	2+	2+	NR	-	2+	2+	2+	2+	2+	2+	2+	-	NR	NR	2+	+	+	-	2+
Parris, 1997	2+	2+	-	-	2+	-	-	2+	2+	NR	-	2+	+	2+	2+	2+	2+	2+	NR	NR	NR	+	+	2+	-	-
Pierce, 2016	2+	+	-	-	2+	NR	-	2+	2+	NR	-	2+	2+	2+	2+	2+	2+	2+	NR	NR	NR	+	NR	NR	-	-
Rogers, 2004	2+	+	NR	-	2+	-	-	2+	2+	NR	-	2+	2+	2+	2+	2+	2+	2+	NR	NR	NR	+	-	-	-	+
Shrimpling, 2002	2+	2+	+	-	2+	-	-	2+	2+	NR	-	2+	+	2+	2+	2+	2+	2+	NR	NR	NR	+	NR	-	-	+
Thurston, 1996	2+	+	2+	+	2+	NR	NR	+	2+	NR	+	2+	+	+	2+	2+	2+	2+	NR	NR	2+	2+	+	2+	+	+

For peer review only

Tsai, 2012	2+	+	+	-	2+	-	-	2+	2+	NR	-	2+	2+	2+	2+	2+	2+	2+	NR	NR	NR	2+	2+	2+	-	+
Tucker, 2015	2+	+	+	-	2+	-	-	2+	2+	NR	-	2+	2+	2+	2+	2+	2+	2+	NR	NR	NR	+	NR	-	-	+
Uthman, 2018	2+	2+	+	-	2+	NR	NR	2+	2+	NR	+	2+	2+	2+	2+	2+	2+	2+	2+	NR	NR	2+	2+	2+	-	+
van den Bersselaar, 2018	2+	2+	+	-	2+	-	NR	2+	2+	NR	-	2+	2+	2+	2+	2+	2+	2+	NR	NR	NR	+	+	-	-	+
van Gils-van Rooij, 2018	+	2+	2+	-	2+	-	NR	2+	2+	NR	-	2+	2+	2+	2+	2+	2+	2+	+	NR	NR	+	2+	+	-	+
Zager, 2018	2+	2+	+	-	2+	-	-	2+	2+	NR	-	2+	2+	2+	2+	2+	2+	2+	NR	NR	NR	+	NR	+	-	+
Kool, 2008	2+	+	-	-	2+	-	-	2+	2+	NR	-	2+	+	-	2+	2+	2+	2+	+	NR	NR	2+	2+	+	-	-
Al Abri, 2020	2+	2+	+	-	2+	-	-	2+	NR	NR	-	2+	2+	2+	2+	2+	2+	2+	+	NR	NR	-	2+	2+	+	+
Ho, 2018	2+	2+	+	+	2+	NR	-	2+	2+	NR	2+	2+	2+	2+	2+	2+	2+	2+	2+	2+	2+	2+	2+	2+	-	+
Li, 2018	+	2+	+	-	2+	-	-	2+	NR	NR	-	2+	2+	2+	2+	2+	2+	2+	-	NR	NR	-	2+	+	-	+
Hackman, 2015	+	+	+	-	2+	-	-	2+	NR	NR	-	2+	2+	2+	2+	2+	2+	2+	-	NR	NR	2+	2+	2+	-	+
Klassen, 1993	2+	2+	+	2+	2+	+	+	2+	NR	NR	2+	2+	2+	2+	2+	2+	2+	2+	2+	-	NR	-	2+	+	-	+
Al Khadi, 2017	+	+	+	-	2+	-	-	2+	NR	NR	-	2+	2+	2+	2+	2+	2+	2+	-	NR	NR	-	2+	+	-	+
Ashurst, 2014	+	2+	+	-	2+	-	-	2+	NR	NR	-	2+	+	2+	2+	2+	2+	2+	-	2+	2+	-	2+	+	-	+
Fan, 2006	+	2+	+	2+	2+	+	+	2+	2+	NR	2+	+	+	+	2+	2+	2+	2+	2+	2+	2+	2+	2+	2+	+	+
Sikkenga, 2016	+	+	+	-	2+	-	-	2+	2+	NR	-	2+	2+	2+	2+	2+	2+	2+	-	NR	NR	NR	2+	2+	-	+
Lee, 1996	+	+	+	-	2+	-	-	2+	2+	NR	-	2+	2+	2+	2+	2+	2+	2+	-	NR	NR	-	2+	2+	-	+

For peer review only

Dixon, 2014	+	2+	+	2+	2+	+	-	2+	2+	NR	2+	2+	2+	2+	2+	2+	2+	2+	2+	-	2+	NR	2+	2+	-	+
Lee, 2014	+	2+	+	+	2+	NR	+	2+	2+	NR	2+	2+	+	2+	2+	2+	2+	2+	2+	NR	2+	+	2+	+	+	+
Adam, 2014	+	2+	+	+	2+	2+	+	2+	2+	NR	2+	2+	2+	2+	2+	2+	2+	2+	2+	NR	2+	+	2+	2+	+	+
Fontanel, 2011	+	2+	+	-	2+	-	-	2+	2+	NR	-	NR	2+	2+	2+	2+	2+	2+	+	NR	NR	NR	NR	2+	-	+
Gaucher, 2010	+	2+	+	-	2+	-	-	2+	2+	NR	-	2+	2+	2+	2+	2+	2+	2+	-	NR	2+	2+	2+	2+	-	+
Demarco, 2010	+	2+	+	-	2+	-	-	2+	2+	NR	-	2+	2+	2+	2+	2+	2+	2+	-	NR	NR	-	2+	+	-	+
Jobe, 2013	+	2+	+	+	2+	NR	NR	2+	NR	NR	2+	2+	2+	+	2+	2+	2+	2+	2+	2+	NR	-	-	-	-	+
Lijuan, 2017	2+	2+	+	-	2+	-	-	2+	2+	NR	-	2+	+	2+	2+	2+	2+	2+	-	NR	NR	-	2+	+	-	+

For peer review only

Appendix Figure 1: Effectiveness of PHCP interventions on time to provide initial assessment (in minutes) sub-grouped by interventions.

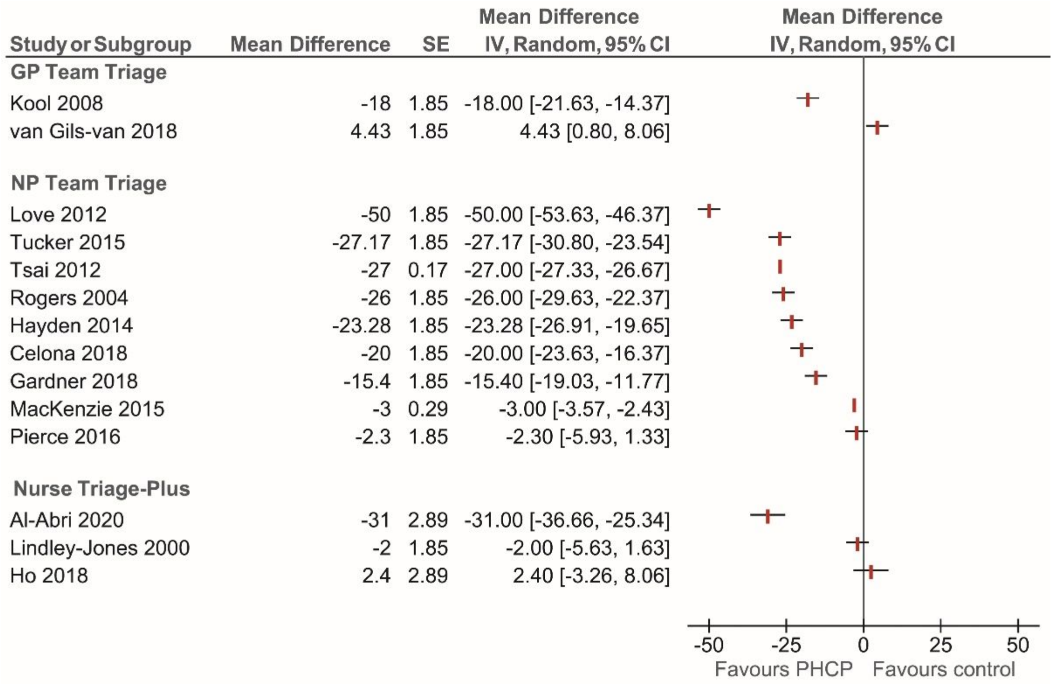

The horizontal black lines represent 95% confidence intervals and the red dots in the middle represents point estimates (mean difference).

review only

Appendix Figure 2: Effectiveness of PHCP interventions on achieving benchmark time to provider initial assessment

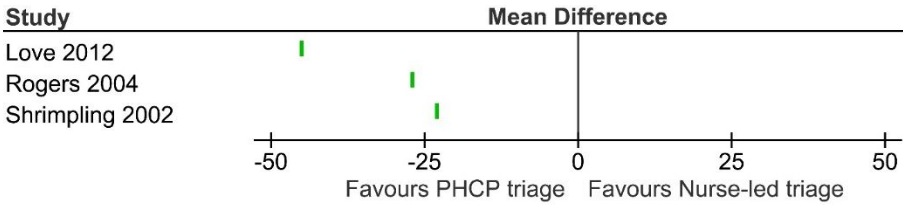

The horizontal black lines represent 95% confidence intervals and the red dots in the middle represents point estimates (mean difference).

For peer review only

Appendix Figure 3: Effectiveness of PHCP interventions on ED LOS (in minutes) sub-grouped by interventions.

Study or Subgroup	Mean Difference	SE	Mean Difference IV, Random, 95% CI
**GP Team Triage**			
van Gils-van 2018	4.52	23.9	4.52 [-42.32, 51.36]
**NP Team Triage**			
Day 2013	-37	0.94	-37.00 [-38.84, -35.16]
Gardner 2018	-34	4.8	-34.00 [-43.41, -24.59]
Hayden 2014	-16.65	23.9	-16.65 [-63.49, 30.19]
MacKenzie 2015	-20	1.13	-20.00 [-22.21, -17.79]
Pierce 2016	-28.5	23.9	-28.50 [-75.34, 18.34]
Rogers 2004	-22	23.9	-22.00 [-68.84, 24.84]
Tsai 2012	-58	0.83	-58.00 [-59.63, -56.37]
Tucker 2015	-18.3	23.9	-18.30 [-65.14, 28.54]
Zager 2018	-102	23.9	-102.00 [-148.84, -55.16]
**Nurse Triage-Plus**			
Adam 2014	-11	5.2	-11.00 [-21.19, -0.81]
Al-Abri 2020	-97	23.9	-97.00 [-143.84, -50.16]
Ashurst 2014	-6.5	23.9	-6.50 [-53.34, 40.34]
Cheung 2002	-46	23.9	-46.00 [-92.84, 0.84]
Demarco 2010	-193	23.9	-193.00 [-239.84, -146.16]
Dixon 2014	-55	23.9	-55.00 [-101.84, -8.16]
Fan 2006	-6.7	7.22	-6.70 [-20.85, 7.45]
Fontanel 2011	-64	23.9	-64.00 [-110.84, -17.16]
Hackman 2015	-10	4.03	-10.00 [-17.90, -2.10]
Ho 2018	-13	4.8	-13.00 [-22.41, -3.59]
Klassen 1993	-18	6.3	-18.00 [-30.35, -5.65]
Lee 1996	-18.6	23.9	-18.60 [-65.44, 28.24]
Lee 2014	-18.59	3.19	-18.59 [-24.84, -12.34]
Lee 2016	-28	23.9	-28.00 [-74.84, 18.84]
Li 2018	-15	0.96	-15.00 [-16.88, -13.12]
Parris 1997	-6	23.9	-6.00 [-52.84, 40.84]
Thurston 1996	-15	2.3	-15.00 [-19.51, -10.49]

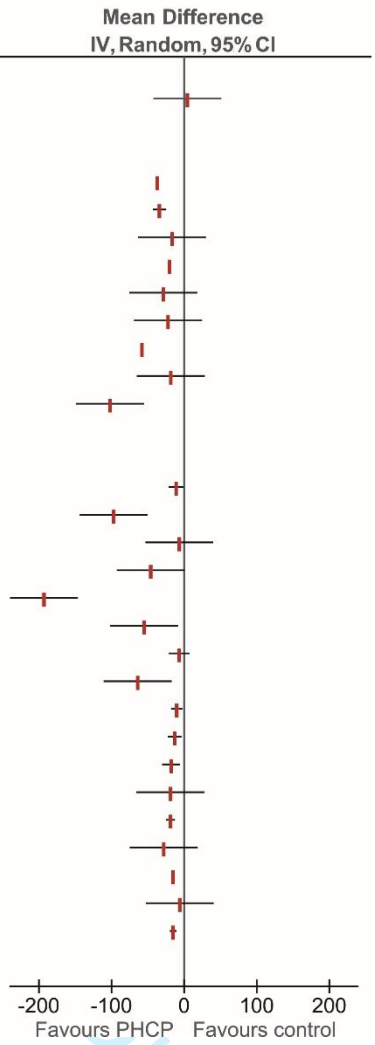

The horizontal black lines represent 95% confidence intervals and the red dots in the middle represents point estimates (mean difference).

References

1. Day TE, Al-Roubaie AR, Goldlust EJ. Decreased length of stay after addition of healthcare provider in emergency department triage: a comparison between computer-simulated and real-world interventions. *Emerg Med J.* 2013;30(2):134-138.
2. Hayden C, Burlingame P, Thompson H, Sabol VK. Improving patient flow in the emergency department by placing a family nurse practitioner in triage: a quality-improvement project. *Journal of Emergency Nursing.* 2014;40(4):346-351.
3. Lindley-Jones M, Finlayson BJ. Triage nurse requested x rays--are they worthwhile? *J Accid Emerg Med.* 2000;17(2):103-107.
4. Pierce BA, Gormley D. Are Split Flow and Provider in Triage Models in the Emergency Department Effective in Reducing Discharge Length of Stay? *Journal of Emergency Nursing.* 2016;42(6):487-491.
5. Tsai VW, Sharieff GQ, Kanegaye JT, Carlson LA, Harley J. Rapid medical assessment: improving pediatric emergency department time to provider, length of stay, and left without being seen rates. *Pediatric Emergency Care.* 2012;28(4):354-356.
6. Zager K, Taylor YJ. Discharge to medical home: A new care delivery model to treat non-urgent cases in a rural emergency department. *Healthcare.* 2018;21:21.
7. Lee WW, Filiatrault L, Abu-Laban RB, Rashidi A, Yau L, Liu N. Effect of Triage Nurse Initiated Radiography Using the Ottawa Ankle Rules on Emergency Department Length of Stay at a Tertiary Centre. *CJEM.* 2016;18(2):90-97.
8. Cheung WWH, Heeney L, Pound JL. An advance triage system. *Accident & Emergency Nursing.* 2002;10(1):10-16.
9. Love RA, Murphy JA, Lietz TE, Jordan KS. The effectiveness of a provider in triage in the emergency department: a quality improvement initiative to improve patient flow. *Advanced Emergency Nursing Journal.* 2012;34(1):65-74.
10. MacKenzie RS, Burmeister DB, Brown JA, et al. Implementation of a rapid assessment unit (intake team): impact on ED length of stay. *American Journal of Emergency Medicine.* 2015;33(2):291-293.
11. van Gils-van Rooij ESJ, Meijboom BR, Broekman SM, Yzermans CJ, de Bakker DH. Is patient flow more efficient in Urgent Care Collaborations? *European Journal of Emergency Medicine.* 2018;25(1):58-64.
12. Lijuan Z. Advanced Triage Protocols in the Emergency Department. *Advanced Triage Protocols in the Emergency Department.* 2017:1-1.
13. Adam H, Tamim H, Altamimi S, et al. Effect of triage nurse ordered distal extremity X-rays on emergency department length of stay: A randomized controlled trial. *Academic Emergency Medicine.* 2014;21(5 SUPPL. 1):S195.
14. Al Kadhi O, Manley K, Natarajan M, et al. A renal colic fast track pathway to improve waiting times and outcomes for patients presenting to the emergency department. *Open access emergency medicine : OAEM.* 2017;9:53-55.
15. Ashurst JV, Nappe T, Digiambattista S, et al. Effect of triage-based use of the Ottawa foot and ankle rules on the number of orders for radiographic imaging. *The Journal of the American Osteopathic Association.* 2014;114(12):890-897.
16. Demarco F, Gerardo CJ, Boardwine A, et al. Effect of a nurse rapid intake initiative on patient length of stay and satisfaction: A project IMPACT initiative. *Academic Emergency Medicine.* 2010;17(SUPPL. 1):S93-S94.

For peer review only

17. Dixon A, Clarkin C, Barrowman N, Correll R, Osmond MH, Plint AC. Reduction of radial-head subluxation in children by triage nurses in the emergency department: A cluster-randomized controlled trial. *CMAJ.* 2014;186(9):E317-E323.
18. Fan J, Woolfrey K. The effect of triage-applied Ottawa Ankle Rules on the length of stay in a Canadian urgent care department: a randomized controlled trial. *Academic emergency medicine : official journal of the Society for Academic Emergency Medicine.* 2006;13(2):153-157.
19. Fontanel A, Besson C, Gallegos C, Vallot C, Droal D. Can X-rays be prescribed by a nurse in an emergency department? *European Journal of Emergency Medicine.* 2011;18(5):310-311.
20. Gaucher N, Bailey B, Gravel J. Triage nurses' counselling influences return visits of children leaving the emergency department before being seen by a physician. *Academic Emergency Medicine.* 2010;17(SUPPL. 1):S118-S119.
21. Hackman JL, Roth ED, Gaddis ML, Gratton MC. The effect of a nurse-initiated chest pain protocol on disposition time: A retrospective review. *Annals of Emergency Medicine.* 2015;66(4 SUPPL. 1):S9-S10.
22. Jobé J, Vandercleyen C, Ghuysen A, D'Orio V. Prospective study of an advanced nurse triage for a target pathology at the admission in the emergency department. *Acta clinica belgica.* 2013;68(6):482-.
23. Klassen TP, Ropp LJ, Sutcliffe T, et al. A randomized, controlled trial of radiograph ordering for extremity trauma in a pediatric emergency department. *Annals of emergency medicine.* 1993;22(10):1524-1529.
24. Lee KM, Wong TW, Chan R, Lau CC, Fu YK, Fung KH. Accuracy and efficiency of X-ray requests initiated by triage nurses in an accident and emergency department. *Accident and emergency nursing.* 1996;4(4):179-181.
25. Lee WW, Filiatrault L, Abu-Laban RB, Rashidi A, Yau L, Liu N. Effect of triage nurse initiated radiography using the Ottawa Ankle Rules on emergency department length of stay at a tertiary care center. *Canadian Journal of Emergency Medicine.* 2014;16(SUPPL. 1):S38.
26. Sikkenga T, Dumkow L, Draper H, et al. Implementation of a nursing triage order to improve utilization of rapid diagnostic testing for chlamydia and gonorrhea in the emergency department. *Open Forum Infectious Diseases.* 2016;3(Supplement 1).
27. Al Abri FH, Muliira JK, Al Awaisi H. Effect of triage nurse-led application of the Ottawa Ankle Rules on number of radiographic tests and length of stay in selected emergency departments in Oman. *Japan journal of nursing science : JJNS.* 2020;17(1):e12270.
28. Ho JK-M, Chau JP-C, Chan JT-S, Yau CH-Y. Nurse-initiated radiographic-test protocol for ankle injuries: A randomized controlled trial. *International emergency nursing.* 2018;41:1-6.
29. Li Y, Lu Q, Du H, Zhang J, Zhang L. The Impact of Triage Nurse-ordered Diagnostic Studies on Pediatric Emergency Department Length of Stay. *Indian journal of pediatrics.* 2018;85(10):849-854.
30. Parris W, McCarthy S, Kelly AM, Richardson S. Do triage nurse-initiated X-rays for limb injuries reduce patient transit time? *Accid Emerg Nurs.* 1997;5(1):14-15.
31. Thurston J, Field S. Should accident and emergency nurses request radiographs? Results of a multicentre evaluation. *J Accid Emerg Med.* 1996;13(2):86-89.
32. Gardner RM, Friedman NA, Carlson M, Bradham TS, Barrett TW. Impact of revised triage to improve throughput in an ED with limited traditional fast track population. *American Journal of Emergency Medicine.* 2018;36(1):124-127.
33. Rogers T, Ross N, Spooner D. Evaluation of a 'see and treat' pilot study introduced to an emergency department. *Accident and Emergency Nursing.* 2004;12(1):24-27.
34. Tucker A, Bernard M. Making the Case for Nurse Practitioners in the Emergency Department: A Clinical Case Study. *Advanced Emergency Nursing Journal.* 2015;37(4):308-312.

For peer review only

35. Tsai VW, Sharieff GQ, Kanegaye JT, Carlson LA, Harley J. Rapid medical assessment: improving pediatric emergency department time to provider, length of stay, and left without being seen rates. *Pediatric emergency care.* 2012;28(4):354-356.
36. Uthman OA, Walker C, Lahiri S, et al. General practitioners providing non-urgent care in emergency department: a natural experiment. *BMJ Open.* 2018;8(5):e019736.
37. Kool RB, Homberg DJ, Kamphuis HC. Towards integration of general practitioner posts and accident and emergency departments: a case study of two integrated emergency posts in the Netherlands. *BMC Health Services Research.* 2008;8:225.
38. MacKenzie RS, Burmeister DB, Brown JA, et al. Implementation of a rapid assessment unit (intake team): impact on ED length of stay. *Am J Emerg Med.* 2015;33(2):291-293.

For peer review only

PRISMA 2009 Checklist

Section/topic	#	Checklist item	Reported on page #
**TITLE**			
Title	1	Identify the report as a systematic review, meta-analysis, or both.	1
**ABSTRACT**			
Structured summary	2	Provide a structured summary including, as applicable: background; objectives; data sources; study eligibility criteria, participants, and interventions; study appraisal and synthesis methods; results; limitations; conclusions and implications of key findings; systematic review registration number.	3
**INTRODUCTION**			
Rationale	3	Describe the rationale for the review in the context of what is already known.	5
Objectives	4	Provide an explicit statement of questions being addressed with reference to participants, interventions, comparisons, outcomes, and study design (PICOS).	7
**METHODS**			
Protocol and registration	5	Indicate if a review protocol exists, if and where it can be accessed (e.g., Web address), and, if available, provide registration information including registration number.	7
Eligibility criteria	6	Specify study characteristics (e.g., PICOS, length of follow-up) and report characteristics (e.g., years considered, language, publication status) used as criteria for eligibility, giving rationale.	7
Information sources	7	Describe all information sources (e.g., databases with dates of coverage, contact with study authors to identify additional studies) in the search and date last searched.	8
Search	8	Present full electronic search strategy for at least one database, including any limits used, such that it could be repeated.	8
Study selection	9	State the process for selecting studies (i.e., screening, eligibility, included in systematic review, and, if applicable, included in the meta-analysis).	8
Data collection process	10	Describe method of data extraction from reports (e.g., piloted forms, independently, in duplicate) and any processes for obtaining and confirming data from investigators.	8
Data items	11	List and define all variables for which data were sought (e.g., PICOS, funding sources) and any assumptions and simplifications made.	7
Risk of bias in individual studies	12	Describe methods used for assessing risk of bias of individual studies (including specification of whether this was done at the study or outcome level), and how this information is to be used in any data synthesis.	9
Summary measures	13	State the principal summary measures (e.g., risk ratio, difference in means).	9

For peer review only

PRISMA 2009 Checklist

Synthesis of results	14	Describe the methods of handling data and combining results of studies, if done, including measures of consistency (e.g., I^2) for each meta-analysis.	Pages 09-10

Section/topic	#	Checklist item	Reported on page #
Risk of bias across studies	15	Specify any assessment of risk of bias that may affect the cumulative evidence (e.g., publication bias, selective reporting within studies).	9
Additional analyses	16	Describe methods of additional analyses (e.g., sensitivity or subgroup analyses, meta-regression), if done, indicating which were pre-specified.	9
**RESULTS**			
Study selection	17	Give numbers of studies screened, assessed for eligibility, and included in the review, with reasons for exclusions at each stage, ideally with a flow diagram.	11
Study characteristics	18	For each study, present characteristics for which data were extracted (e.g., study size, PICOS, follow-up period) and provide the citations.	11
Risk of bias within studies	19	Present data on risk of bias of each study and, if available, any outcome level assessment (see item 12).	12
Results of individual studies	20	For all outcomes considered (benefits or harms), present, for each study: (a) simple summary data for each intervention group (b) effect estimates and confidence intervals, ideally with a forest plot.	13, 14
Synthesis of results	21	Present results of each meta-analysis done, including confidence intervals and measures of consistency.	13, 14, 15
Risk of bias across studies	22	Present results of any assessment of risk of bias across studies (see Item 15).	12
Additional analysis	23	Give results of additional analyses, if done (e.g., sensitivity or subgroup analyses, meta-regression [see Item 16]).	13, 14
**DISCUSSION**			
Summary of evidence	24	Summarize the main findings including the strength of evidence for each main outcome; consider their relevance to key groups (e.g., healthcare providers, users, and policy makers).	17
Limitations	25	Discuss limitations at study and outcome level (e.g., risk of bias), and at review-level (e.g., incomplete retrieval of identified research, reporting bias).	20
Conclusions	26	Provide a general interpretation of the results in the context of other evidence, and implications for future research.	21
**FUNDING**			
Funding	27	Describe sources of funding for the systematic review and other support (e.g., supply of data); role of funders for the systematic review.	23

From: Moher D, Liberati A, Tetzlaff J, Altman DG, The PRISMA Group (2009). Preferred Reporting Items for Systematic Reviews and Meta-Analyses: The PRISMA Statement. PLoS Med 6(7): e1000097.

For peer review only - http://bmjopen.bmj.com/site/about/guidelines.xhtml

PRISMA 2009 Checklist

doi:10.1371/journal.pmed1000097

For more information, visit: **www.prisma-statement.org**.

For peer review only

BMJ Open

The impact of employing primary healthcare professionals in emergency department triage on patient flow outcomes: A systematic review and meta-analysis

Journal:	*BMJ Open*
Manuscript ID	bmjopen-2021-052850.R1
Article Type:	Original research
Date Submitted by the Author:	27-Jan-2022
Complete List of Authors:	Jeyaraman, Maya; University of Manitoba, George and Fay Yee Center for Healthcare Innovation<br>Alder, Rachel; Max Rady College of Medicine, Rady Faculty of Health Sciences, University of Manitoba<br>Copstein, Leslie; George and Fay Yee Center for Healthcare Innovation, University of Manitoba<br>Al-Yousif, Nameer; George and Fay Yee Center for Healthcare Innovation, University of Manitoba<br>Suss, Roger; Department of Family Medicine, Rady Faculty of Health Sciences, University of Manitoba<br>Zarychanski, Ryan ; Department of Medical Oncology and Hematology, Cancer Care Manitoba<br>Doupe, Malcolm; Department of Community Health Sciences, University of Manitoba, Winnipeg, Manitoba<br>Berthelot, Simon; Centre de recherche du CHU de Québec-Université Laval, Axe Santé des populations et Pratiques optimales en santé<br>Mireault, Jean; HEC Pôle santé, Université de Montréal<br>Tardif, Patrick; Department of Emergency Medicine, Cité de la santé de Laval<br>Askin, Nicole; WRHA Virtual Library, University of Manitoba<br>Buchel, Tamara; Manitoba College of Family Physicians<br>Rabbani, Rasheda; George and Fay Yee Center for Healthcare Innovation, University of Manitoba<br>Beaudry, Thomas; Patient and Public Engagement Collaborative Partnership, George & Fay Yee Center for Healthcare Innovation<br>Hartwell, Melissa; rimary and Integrated Health care Innovation Network<br>Shimmin, Carolyn; George and Fay Yee Center for Healthcare Innovation<br>Edwards, Jeanette; Community Health Quality and Learning, Shared Health Manitoba<br>Halas , Gayle; Manitoba Primary and Integrated Health care Innovation Network<br>Sevcik, William; Department of Emergency Medicine, Faculty of Medicine & Dentistry, University of Alberta<br>Tricco, Andrea; Knowledge Translation Program, St. Michael's Hospital Li Ka Shing Knowledge Institute, Unity Health Toronto<br>Chochinov, Alecs; epartment of Emergency Medicine, Faculty of Medicine, University of Manitoba<br>Rowe, Brian; Department of Emergency Medicine, Faculty of Medicine & Dentistry, University of Alberta; School of Public Health, University of

	Alberta<br>Abou-Setta, Ahmed; George and Fay Yee Center for Healthcare Innovation, University of Manitoba

**Primary Subject Heading**:	Emergency medicine
Secondary Subject Heading:	Evidence based practice, Health services research
Keywords:	ACCIDENT & EMERGENCY MEDICINE, INTENSIVE & CRITICAL CARE, Adult intensive & critical care < INTENSIVE & CRITICAL CARE

SCHOLARONE™
Manuscripts

I, the Submitting Author has the right to grant and does grant on behalf of all authors of the Work (as defined in the below author licence), an exclusive licence and/or a non-exclusive licence for contributions from authors who are: i) UK Crown employees; ii) where BMJ has agreed a CC-BY licence shall apply, and/or iii) in accordance with the terms applicable for US Federal Government officers or employees acting as part of their official duties; on a worldwide, perpetual, irrevocable, royalty-free basis to BMJ Publishing Group Ltd ("BMJ") its licensees and where the relevant Journal is co-owned by BMJ to the co-owners of the Journal, to publish the Work in this journal and any other BMJ products and to exploit all rights, as set out in our licence.

The Submitting Author accepts and understands that any supply made under these terms is made by BMJ to the Submitting Author unless you are acting as an employee on behalf of your employer or a postgraduate student of an affiliated institution which is paying any applicable article publishing charge ("APC") for Open Access articles. Where the Submitting Author wishes to make the Work available on an Open Access basis (and intends to pay the relevant APC), the terms of reuse of such Open Access shall be governed by a Creative Commons licence – details of these licences and which Creative Commons licence will apply to this Work are set out in our licence referred to above.

Other than as permitted in any relevant BMJ Author's Self Archiving Policies, I confirm this Work has not been accepted for publication elsewhere, is not being considered for publication elsewhere and does not duplicate material already published. I confirm all authors consent to publication of this Work and authorise the granting of this licence.

For peer review only

The impact of employing primary healthcare professionals in emergency department triage on patient flow outcomes: A systematic review and meta-analysis

Review Authors: Maya M. Jeyaraman[1], Rachel N. Alder[2], Leslie Copstein[1], Nameer Al-Yousif[1], Roger Suss[3], Ryan Zarychanski[4], Malcolm Doupe[5], Simon Berthelot[6], Jean Mireault[7], Patrick Tardif[8], Nicole Askin[9], Tamara Buchel[10], Rasheda Rabbani[1], Thomas Beaudry[11], Melissa Hartwell[12], Carolyn Shimmin[1], Jeanette Edwards[13], Gayle Halas[14], William Sevcik[15], Andrea C. Tricco[16], Alecs Chochinov[17], Brian H. Rowe[15,18], Ahmed M. Abou-Setta[1]

Affiliations:

[1] George & Fay Yee Center for Healthcare Innovation, University of Manitoba, Winnipeg, Manitoba;

[2] Max Rady College of Medicine, Rady Faculty of Health Sciences, University of Manitoba, Winnipeg, Manitoba;

[3] Department of Family Medicine, Rady Faculty of Health Sciences, University of Manitoba, Winnipeg, Manitoba;

[4] Department of Medical Oncology and Hematology, Cancer Care Manitoba, Winnipeg, Manitoba;

[5] Department of Community Health Sciences, University of Manitoba, Winnipeg, Manitoba;

[6] Centre de recherche du CHU de Québec-Université Laval, Axe Santé des populations et Pratiques optimales en santé, Laval, Québec;

[7] HEC Pôle santé, Université de Montréal, Montreal, Quebec;

[8] Department of Emergency Medicine, Cité de la santé de Laval, Laval, Quebec;

[9] WRHA Virtual Library, University of Manitoba, Winnipeg, Manitoba;

[10] Manitoba College of Family Physicians, Winnipeg, Manitoba;

[11] Patient and Public Engagement Collaborative Partnership, George & Fay Yee Center for Healthcare Innovation, Winnipeg, Manitoba;

[12] Primary and Integrated Health care Innovation Network, Edmonton, Alberta;

[13] Community Health Quality and Learning, Shared Health Manitoba, Winnipeg, Manitoba;

[14] Manitoba Primary and Integrated Health care Innovation Network, Winnipeg, Manitoba;

[15] Department of Emergency Medicine, Faculty of Medicine & Dentistry, University of Alberta, Edmonton, Alberta;

For peer review only - http://bmjopen.bmj.com/site/about/guidelines.xhtml

For peer review only

[16] Knowledge Translation Program, St. Michael's Hospital Li Ka Shing Knowledge Institute, Unity Health Toronto, Toronto, Ontario;

[17] Department of Emergency Medicine, Faculty of Medicine, University of Manitoba, Winnipeg, Manitoba;

[18] School of Public Health, University of Alberta, Edmonton, Alberta;

all in *Canada.*

.

Corresponding author: Maya Jeyaraman, MD PhD

George & Fay Yee Center for Healthcare Innovation

753 McDermot Ave, Winnipeg MB R3E 0T6

Email: maya.jeyaraman@umanitoba.ca

Phone: (204) 594-5362; Fax: (204) 594-5394.

Short Title: Impact of employing primary healthcare professionals in emergency department triage.

Key Words: ED overcrowding, ED Patient flow, ED Triage, Primary healthcare provider

Total word count: 4398 words; **Abstract**: 352 words**; Figures**: 4; **Tables**: 3

For peer review only

Abstract

Objectives: To identify, critically-appraise and summarize evidence on the impact of employing primary healthcare professionals (PHCPs: family physicians/general practitioners (GP), nurse practitioners (NP) and nurses with increased authority) in the emergency department (ED) triage, on patient flow outcomes.

Methods: We searched Medline (Ovid), EMBASE (Ovid), Cochrane Library (Wiley) and CINAHL (EBSCO) (inception to January 2020). Our primary outcome was the time to provider initial assessment (PIA). Secondary outcomes included time to triage, proportion of patients leaving without being seen (LWBS), length of stay (ED LOS), proportion of patients leaving against medical advice (LAMA), number of repeat ED visits, and patient satisfaction. Two independent reviewers selected studies, extracted data, and assessed study quality using the NICE quality assessment tool.

Results: From 23,973 records, 40 comparative studies including 10 randomized controlled trials (RCTs) and 13 pre-post studies were included. PHCP interventions were led by NP (n=14), GP (n=3) or nurses with increased authority (n=23) at triage. In all studies PHCP-led intervention effectiveness was compared to the traditional nurse-led triage model. Median duration of the interventions was 6 months. Study quality was generally low (confounding bias); 7 RCTs were classified as moderate quality. Most studies reported that PHCP-led triage interventions decreased the PIA (13/14), ED LOS (29/30), proportion of patients LWBS (8/10), time to triage (3/3), and repeat ED visits (5/6), and increased the patient satisfaction (8/10). The proportion of patients LAMA did not differ between groups (3/3). Evidence from RCT’s (n=8) as well as other study designs showed a significant decrease in ED LOS favoring the PHCP-led interventions.

For peer review only - http://bmjopen.bmj.com/site/about/guidelines.xhtml

Conclusions: Overall, PHCP-led triage interventions improved ED patient flow metrics. There was a significant decrease in ED LOS irrespective of the study design, favoring the PHCP-led interventions. Evidence from well-designed high quality RCTs is required prior to widespread implementation.

PROSPERO trial registration number: CRD42020148053

For peer review only

For peer review only - http://bmjopen.bmj.com/site/about/guidelines.xhtml

Article Summary

Strengths and limitations of this study

- The main strength of our systematic review is that our study team engaged and collaborated with patient and public partners during the design, conduct and dissemination phases of the study by following the criteria identified for patient-oriented research which emphasizes the active and meaningful engagement of patients as research partners.
- This systematic review was conducted using the rigorous Cochrane systematic review methodology and used an a priori registered protocol.
- A main limitation of this systematic review is that we did not include non-English language publications.

For peer review only

For peer review only - http://bmjopen.bmj.com/site/about/guidelines.xhtml

INTRODUCTION

Healthcare systems worldwide experience emergency departments (ED) overcrowding[1-5] which impacts the timely delivery of healthcare[6,7], patient and provider dissatisfaction[8], and other adverse outcomes[9]. ED overcrowding is a complex phenomenon and is associated with input (increased patient volume), throughput (ED boarding), and especially output (lack of hospital beds) factors, as well as system-wide influences[10]. A large volume of lower acuity patients presenting to ED leads to demand-capacity mismatch and entry block (e.g., delays in ED assessment)[10,11].

Lower acuity ED patients generally include patients: a) having low acuity triage codes; b) being discharged quickly; or c) being seen by an alternative primary healthcare provider[12]. These alternative primary healthcare providers are typically physicians (family physicians/general practitioners [GP]), nurse practitioners (NP), nurses with increased authority, or physician assistants who are legally authorized to provide or coordinate healthcare to patients[13]. Studies have reported that 8-62% of all ED presentations are lower acuity[14-17]. With ED visits increasing by 20% each year, along with a decrease in operational EDs[18], lower acuity visits may lead to unnecessary diagnostic testing, greater healthcare spending, lost opportunity for continuity of care with primary care physicians, sub-optimal care due to hurried management, and prolonged ED length of stay (LOS)[12,14,19,20]. Increased demand for ED services also leads to increased ED wait times and patients choosing to leave ED without being-seen, thus potentially compromising patient safety[18].

Worldwide, there is growing interest in interventions and strategies, either to discourage lower acuity ED visits or to reduce the impact of lower acuity visits in the ED by improving patient flow. Studies have investigated the impact of interventions such as public and patient

For peer review only - http://bmjopen.bmj.com/site/about/guidelines.xhtml

education[14], financial disincentives (higher co-payments for lower acuity ED visits)[21], increasing after-hours primary care[22], patient redirection to non-ED care alternatives[23], and advanced access[24,25] to discourage unnecessary ED utilization. Since EDs have no control over the volume of presenting patients and ED presentations continue to be on the rise[14,18], recommendations have been made to focus on strategies to improve patient flow within the ED[26]. Studies have investigated various strategies to improve ED patient flow, including triage related interventions[8,27].

While the precise role of the NPs, GPs or nurses given increased authority (all referred to as primary healthcare professionals [PHCPs]) in an ED is unclear, they may provide potential benefits to improve ED times and outcomes. Studies have reported the following roles of the PHCPs at ED triage: (1) GP either triaging (seeing and treating, streaming) or supervising triage[28-30]; (2) NP either alone or working alongside a triage nurse (ordering investigations, streaming, seeing and treating, or assessing patients and discharging/re-directing) [18-20,26,31-40]; (3) Triage nurse with increased authority given extra capacities outside of their usual scope of practice to order investigations for patients before streaming to the ED MD[41-63]. Although, many primary research studies have investigated the impact of PHCP's[20,33,37,38,41,64,65] at triage on ED patient flow, to the best of our knowledge there are no systematic reviews that have summarized evidence from these studies.

The main objective of our systematic review was to identify, critically-appraise and summarize evidence on the effectiveness of employing PHCP's at ED triage to improve ED patient flow metrics.

For peer review only - http://bmjopen.bmj.com/site/about/guidelines.xhtml

METHODS

Using an *a priori* systematic review protocol (CRD42020148053) developed in collaboration with patient partners, we conducted this review according to guidelines enumerated in the Methodological Expectations of Cochrane Intervention Reviews (MECIR). Our systematic review is reported using the Preferred Reporting Items for Systematic reviews and Meta-Analyses (PRISMA) guideline[66].

Eligibility criteria

We included comparative studies (only English language) of any ED triage intervention that involved a PHCP and was designed to improve ED (adult and pediatric) patient flow metrics. We excluded primary studies involving exclusively emergency physicians (ED MD), such as the triage liaison physician (TLP)[27]. The primary outcome was the time to provider initial assessment (PIA: time from ED arrival to the time when the patient is first assessed by an ED provider (ED MD, NP, or a GP in the ED)). Secondary outcomes were ED length of stay (LOS: time from ED arrival to disposition), the proportion of patients who left without being seen (LWBS), proportion of patients leaving against medical advice (LAMA), time to triage, number of repeat ED visits, and patient satisfaction. The outcome measures were selected *a priori* in collaboration with the patient partners in the research team. We have reported a more detailed list of the inclusion and exclusion criteria in ***Appendix Table 1***.

Literature search methods for identifying relevant citations

In conjunction with a health librarian (TR) we designed a search strategy for Medline (Ovid) to identify literature relevant to the objective (from inception until June 2018, and later updated in

For peer review only - http://bmjopen.bmj.com/site/about/guidelines.xhtml

January 2020). Since most of the potentially relevant studies would be performed in the US, Europe and Commonwealth countries, search results were limited to English language publications. Our Medline search was peer-reviewed by a second librarian (JJ)[67], principal investigators (MJ, AMAS), and patient partners (MH, TB). Once finalized, the Medline search strategy (***Appendix Table 2***) was adapted for replication in the following databases: EMBASE (Ovid), Cochrane Library (Wiley), and CINAHL (EBSCO). An experienced librarian (NA) searched the included databases up to January 2020. The bibliographic search was supplemented with searching the grey literature (i.e., difficult to locate unpublished studies) as listed in ***Appendix Table 3.*** We also searched the reference lists of all the included publications for additional relevant studies. We used EndNote™ (Version X7, Thomson Reuters) for reference management.

Selection of sources of evidence

Two reviewers (RA & (LC or NA)) independently screened the titles and abstracts, and full texts of relevant citations using pilot tested screening forms. Any disagreement on inclusion was resolved through consensus or third party (MJ) adjudication.

Data Extraction, Data analysis and Quality assessment

Standardized data extraction forms were developed to record data from each of included studies after pilot testing. At least two review authors independently extracted baseline characteristics (RA, LC, NA), outcome data (RA, LC) and assessed methodological quality (MJ, RA, LC) on these studies. Disagreements among reviewers were resolved through consensus or third-party adjudication (MJ or AMAS). A meta-analysis of mean differences (MD) in ED times with 95%

For peer review only - http://bmjopen.bmj.com/site/about/guidelines.xhtml

confidence intervals (CIs) was planned *a priori* to derive pooled summary estimates. Heterogeneity among included studies was quantified and tested using I-squared (I^2) statistic and chi-squared statistic, respectively. An I^2 value >50% was considered high heterogeneity; we made an *a priori* methodological decision that heterogeneity indicated by $I^2 > 50\%$ was too high to justify data pooling to generate a summary measure. For studies that did not report any measure of variance we imputed the largest standard error (SE) from among the included studies. In the event that meta-analysis was not possible, the effect estimates (mean differences and SE) from included studies reporting data for the primary outcome and ED LOS were depicted in the form of a forest plot for various a priori subgroups (study designs or PHCP interventions). In these cases, where appropriate, the median of the primary study outcome was reported as the average measure.

We assessed the included studies using the National Institute for Health and Care Excellence (NICE) quality appraisal tool for quantitative studies of intervention[68] as it can be used for multiple study designs. A detailed description is reported under ***Appendix methods***.

Patient and Public Involvement

We collaborated with a diverse group of 13 patient partners (self-identified as Indigenous, Immigrant, White and/or living with disability) during the design phase and the conduct phase of this project, to refine the review question, refine the inclusion criteria, and select patient-important outcomes. Two (TB, MH) of these patient partners collaborated and supported our grant application to obtain funding for this project. During the conduct phase of this systematic review three patient partners helped refine the search strategy (by identifying missing search terms and suggesting additional search terms in the preliminary search strategy), review to

For peer review only - http://bmjopen.bmj.com/site/about/guidelines.xhtml

confirm included studies, and in knowledge dissemination (co-presented abstract at a conference and co-authoring the manuscript). We have reported the patient partner involvement in this systematic review according to GRIPP2 checklist (short form)[69].

For peer review only

For peer review only - http://bmjopen.bmj.com/site/about/guidelines.xhtml

RESULTS

We identified 23,973 relevant citations from database search, of which 40 met the inclusion criteria[18,20,26,28-34,36-63,70,71] (44 study reports). The study selection process is reported using the PRISMA study flow chart (***Figure 1***).

Study characteristics

Included studies were full-length journal articles[18,20,26,28-34,36-45,47-49,51,52,56,58,59,61,70,71] (n = 31; 77.5%), abstracts (n=8)[46,50,53-55,57,60,63] or thesis (n=1)[62] published from 1993 to 2020. More than half were conducted in North America[18,26,31,32,34,36,39-42,49,51,52,54,55,58,60,62,63,70,71] (n = 21; 52.5%), and the rest were conducted in Europe[20,28-30,37,38,43,45,48,53,57] (n = 11; 27.5%), Asia[56,59,61] (n = 3; 7.5%), Australia[33,44] (n = 2; 5%), Middle East[46,47] (n = 2; 5%) or the location was not reported[50] (n = 1; 2.5%) (***Table 1***). Most studies utilized a pre-post intervention design[18,26,31-34,37-40,62,70,71] (n = 13; 32.5%) and the remaining were characterized as randomized controlled trials (RCT)[42,43,45,46,51,52,56-58,60] (n = 10; 25%), observational retrospective cohort studies[20,29,41,48,54,55,61,63] (n = 8, 20%), controlled before and after studies (CBA)[28,36,47,53] (n = 4; 10%), quasi-randomized trials[44] (n = 1; 2.5%) observational prospective cohort studies[49,50,59] (n = 3; 7.5%), or cross-sectional observational studies[30] (n = 1; 2.5%). The median duration of intervention reported among included studies was 6 months (range: 2.5 days to 17 months). Studies were mostly conducted in urban EDs[20,26,29,31-34,36-39,41-47,49-52,55,58-60,63,71] (n = 28; 70%), one (2.5%) was conducted in a rural ED and two[28,30] (5%) were conducted in a combination of urban and rural facilities; nine[18,48,53,54,56,57,61,62,70] (22.5%) studies did not report their setting. We classified the EDs reported in the included studies into pediatric EDs (age < 18)[46,51,54,58,61,71] (n = 6; 15%), mixed EDs seeing both adults and children[18,33,49,53,56,59] (n = 6; 15%) and adult only EDs[31,34,42,44,47] (n = 5; 12.5%), depending on the age of the population that the EDs served. More

For peer review only - http://bmjopen.bmj.com/site/about/guidelines.xhtml

than half of the studies (n = 23, 57.5%) did not specifically report the age of the population that their EDs served[20,26,28-30,32,36-41,43,45,48,50,52,55,57,60,62,63,70]. The majority (n = 15, 38%) of included studies[20,28,29,31,33,34,37-40,44,45,47,56,61] reported enrolling only patients with triage category 4-5 (additional details reported in ***Appendix Results 1***).

The majority (82.5%) of included studies were of low methodological quality and the remaining seven (17.5%) included studies (RCTs)[42,43,45,46,52,56,60] were of a moderate methodological quality (***Table 1 & Appendix Table 4***).

We categorized the triage interventions involving PHCP reported by the included studies, in comparison to the traditional (nurse-led) triage model (***Figure 2),*** as follows: (1) *GP team-triage*[28-30] *(n = 3, 7%)*: where GP was involved in the ED triage (triaging or supervising triage) either seeing and treating low-acuity patients or streaming moderate to high-acuity patients to the ED MD; (2) *NP team-triage*[18-20,26,31-40] *(n =14, 35%):* where the NP was located at the ED triage area working alongside a triage nurse, either ordering investigations at triage before streaming to ED MD, seeing and treating low-acuity patients, directing low-acuity patients to a GP located within ED for treatment, or assessing patients and discharging/re-directing with a same day appointment with a GP at an adjoining GP clinic; (3) *Nurse triage-plus*[41-63] *(n = 23, 58%)*: triage nurse with increased authority (extra capacities outside of their usual scope of practice) to order investigations for patients before streaming to the ED MD. The traditional ED care model with an ED nurse-led triage followed by the ED MD assessment was considered standard of care and the comparator in all the included studies (***Figure 2)***.

PIA

For peer review only - http://bmjopen.bmj.com/site/about/guidelines.xhtml

Fourteen studies[18,26,28,30,31,34,36,37,39,43,47,56,70,71] (35%) reported the effect of PHCP triage interventions on PIA in comparison to a traditional nurse-led triage. Using a forest plot, we depicted the effectiveness of the PHCP triage interventions on PIA sub-grouped by study design (***Figure 3***). Two RCTs[43,56] (of moderate quality), reported a non-significant small decrease in PIA in the PHCP triage intervention (nurse triage-plus) group compared to the traditional nurse-led triage model (mean difference (MD) -0.36 minutes [95% CI -4.53 to 3.81]; 2 studies; I^2: 39%; P=0.20; moderate quality). Three CBA studies[28,36,47] (low quality) reported a decrease in PIA in the PHCP triage intervention group (median [range] = -18 minutes [-2.3 to -31]).

All eight pre-post studies[18,26,31,34,37,39,70,71] (low quality) reported a significant decrease in PIA (median [range] = -24.65 minutes [-3 to -50]) in the PHCP triage intervention group, compared to the traditional nurse-led triage model. Exploration of heterogeneity among pre-post studies (I^2: 100%) revealed four studies[26,31,34,70] that contributed to all the observed heterogeneity; however, we were unable to identify specific reasons for heterogeneity. A sensitivity analysis without these four studies showed a significant mean decrease of PIA by 26 minutes favoring the PHCP triage intervention group (NP team triage). One cross-sectional observational study[30] (low quality) failed to identify a difference in PIA in the PHCP intervention group (4.4 minutes). The results for PIA sub-grouped by various PHCP interventions is reported under ***Appendix Results 2.*** We have depicted the effectiveness of each of the three models of PHCP triage interventions on PIA separately using a forest plot (***Appendix Figure 1***).

Three studies[26,33,37,38] reported greater percentage of patients seen within benchmark times in the NP team triage intervention groups compared to the traditional nurse-led triage model (***Appendix Figure 2***). A fourth study[33] reported that greater percentage of patients (all

For peer review only - http://bmjopen.bmj.com/site/about/guidelines.xhtml

ATS categories) were seen within benchmark times in the NP team triage group compared to the traditional nurse-led triage group (data not shown).

ED LOS

ED LOS was reported by thirty studies (75%)[18,19,30,32,34-37,39-42,44-53,55-61,63]. Using a forest plot, we have depicted the effectiveness of the PHCP triage interventions on ED LOS sub-grouped by study design (***Figure 4***). Eight RCTs[42,45,46,51,52,56,58,60] (six[42,45,46,52,56,60] of moderate quality and two[51,58] of low quality), reported a significant decrease in ED LOS (MD -15.31 minutes [95% CI -18.35 to -12.27]; 8 studies; I^2: 0%; P<0.00001) in the PHCP triage intervention (nurse triage-plus) group compared to the traditional nurse-led triage model. The CBA studies[36,47,53] (low quality) reported a significant decrease in ED LOS (mean difference -63.17 minutes [95% CI -101.93 to -24.40]; 3 studies; I^2: 51%; P=0.001) in the PHCP triage intervention group (2 nurse-triage plus and one NP team triage) compared to the traditional nurse-led triage model, and the three retrospective cohorts[41,55,61] (low quality) also reported a significant decrease in the ED LOS (MD -13.96 minutes [95% CI -19.31 to -8.61]; 3 studies; I^2: 37%; P<0.00001) in the PHCP triage intervention group (nurse triage-plus), compared to the traditional nurse-led triage model.

Among eight pre-post studies[18,32,34,37,39,40,70,71] (low quality), all reported a decrease (5 were significant) in ED LOS (median [range] = -28 minutes [-16.65 to -102) favoring the PHCP triage intervention group (NP team triage). Exploration of heterogeneity among pre-post studies (I^2: 99%) revealed four studies[32,34,40,71] that contributed to all the observed heterogeneity; however, we were unable to identify specific reasons for heterogeneity. A sensitivity analysis without these four studies showed a significant mean decrease of ED LOS by 17 minutes favoring the PHCP triage intervention group (NP team triage). One quasi-RCT[44], and one cross-

For peer review only - http://bmjopen.bmj.com/site/about/guidelines.xhtml

sectional observational study[30] reported no significant differences in ED LOS between comparison groups. Among the three prospective observational cohorts[49,50,59], one reported significant decrease in ED LOS whereas other two reported a non-significant decrease in ED LOS favoring PHCP intervention group. The ED LOS sub-grouped by various PHCP interventions is reported under ***Appendix Results 3***. We have depicted the effectiveness of each of the three models of PHCP triage interventions on ED LOS separately, using a forest plot (***Appendix Figure 3***).

Other outcomes

Ten studies[18-20,26,31,34,35,39,61,62] reported data for percentage of patients LWBS (***Table 2).*** Eight studies reported a reduction in percentage of patient LWBS in the NP team triage intervention group, except one[18] (five[19,20,26,34,35] reported statistically significant decrease). Two[61,62] studies reported a non-significant decrease in percentage of patients LWBS in nurse triage-plus intervention group. The median effect of all estimates is a reduction in LWBS of -2.31% (IQR: -0.39, -3.77). Three pre-post studies[18,35,39] reported the effect of NP team triage intervention on percentage of patients discharged as LAMA (***Table 3***). One study showed a non-significant decrease favoring the intervention group and another two showed a non-significant increase in the percentage of patients LAMA.

Six studies[28,29,32,39,40,54] reported the impact of PHCP interventions on the number of repeat ED visits, and the majority of them reported a decrease in the number of repeat ED visits after PHCP intervention (***Appendix Results 4***). Ten studies[18,28,34,39,42,49,50,52,60,62] reported the effect of PHCP intervention on patient satisfaction, and the majority of them reported an increase in patient satisfaction (***Appendix Results 5***). Three studies[35,37,56] reported the impact of PHCP

For peer review only - http://bmjopen.bmj.com/site/about/guidelines.xhtml

interventions on the time to triage, and all of them reported a decrease in the time to triage after PHCP intervention (***Appendix Results 6***).

For peer review only

For peer review only - http://bmjopen.bmj.com/site/about/guidelines.xhtml

DISCUSSION

Main Findings

This systematic review has summarized the best available evidence from 40 unique comparative studies on the effectiveness of the PHCP triage interventions to improve ED patient flow metrics and mitigate the negative impacts of ED overcrowding. The findings in this systematic review shows that the PHCP-led triage interventions significantly decrease the ED LOS and lead to improvements in key ED patient flow metrics such as PIA, proportion of patients who LWBS, triage time, ED visits and patient satisfaction.

Although this systematic review highlights the positive impact of three unique PHCP triage models on key ED patient flow metrics, it is important to note that the most comprehensive evidence (data for the primary review outcome and all of the secondary outcomes) was available mainly for the nurse triage-plus and NP team triage models, with the least evidence available for the GP team triage model.

Comparison with Other Reviews

To the best of our knowledge this is the first review to investigate specific triage interventions involving NPs and GPs. Previous work had focussed specifically on the impact of TLP[27] or triage nurse ordering[72] on ED patient flow metrics. In 2011, Rowe et al.[27] investigated the impact of TLP's and reported reductions in ED LOS and PIA. However, the interventions mainly involved emergency physicians. Previously, Jennings et al[73] published a systematic review on the impact of emergency NP services in the ED and narratively concluded that although not enough data were available for meta-analysis, NPs within ED may have a positive impact on waiting times, patient satisfaction, and quality of care. Again, this review did not focus on NP at triage. A recent Cochrane review investigated the role of primary care professionals

For peer review only - http://bmjopen.bmj.com/site/about/guidelines.xhtml

(emergency NP and GP) in the ED[74] and concluded that due to limited evidence and suspected bias in allocations of ED patients it was unclear if hiring primary care professionals would decrease PIA, ED LOS, and other ED metrics. It is important to note, however, that this Cochrane review did not investigate the role of primary care professionals at ED triage.

ED LOS and PIA

ED wait times for care delivery is a key performance indicator in many ED settings and our systematic review findings indicate that the PHCP-led triage intervention consistently decreases ED wait times (PIA) and ED LOS. In this review, pre-post studies contributed to the majority of the evidence for effectiveness of PIA (NP team triage). Although heterogeneous and of low quality, the results indicate important potential for the role of NPs in the triage process to reduce ED wait times, improve patient satisfaction and other key ED metrics. A significant decrease in ED LOS was observed with the RCTs (median: -16.8 minutes) although this was comparatively smaller than the significant decrease observed with the CBA (median: -64 minutes) or the pre-post studies (median: -28 minutes). As the minimal clinically important difference in ED LOS is generally accepted to be approximately 30 minutes (clinically significant), the PHCP-led triage interventions could potentially have a positive impact on ED LOS, if implemented.

Type of PHCP

One may argue that similar results could be seen with ED MD at triage. Although true, the cost of adding an NP could be far less than adding an ED MD[39]. In our review, we found only three studies reporting evidence on the role of GP's in ED triage, with one CBA study[28] reporting statistically significant decrease in PIA, and a cross-sectional study[30] reporting an increase in PIA when triaged and treated by a GP. The increase in PIA, however, was reported to be due to an increase in the number of self-referrals in order to be seen by the GP involved in

For peer review only - http://bmjopen.bmj.com/site/about/guidelines.xhtml

triage and treatment of low-acuity patients[30]. In the reported GP team triage interventions, an ED and a GP clinic were co-located and had a joint common entrance, with the GP assistant (supervised by GP) and/or a GP being responsible for the triage of patients (for both ED and GP clinic) and for the treatment of low-acuity patients. The third study[29] reported that GP team triage and x-ray requests at the joint triage reduced the annual ED patient visits. High-quality studies investigating the effectiveness of GPs at ED triage would be valuable.

In our review, the evidence on effectiveness of nurse triage-plus model came mostly from moderate quality studies (RCT or CBA) and showed significant decrease in PIA, ED LOS, and an improved patient satisfaction. Many factors such as patient acuity, EMS traffic/volume and referral patterns often dictate the degree of ED crowding and each ED has their own "signature". For example, in settings where most patients present with ambulatory, single system problems, a nurse triage with extra skills might be effective. Conversely, a nurse triage-plus intervention may be less effective when faced with the challenges of an ED setting with high volumes of trauma, EMS traffic, and high acuity patients.

It would be generally expected that the addition of any qualified staff in the ED, including addition of NP in triage, would tend to make efficiency of the ED operations better. Although we did not assess staff satisfaction in our review, it would be intuitive to think that the addition of NP in the ED triage may also help improve ED staff satisfaction. Many government-funded EDs are cash-constrained and often cannot add additional resource without strong justification and/or reducing funding elsewhere. While addition of NPs, TLPs, or GPs at triage may help, there is still lack of published comparative effectiveness and economic evaluation research to produce a clear cost-effectiveness recommendation. While comparative effectiveness research may prove logistically difficult in the ED and outcome measurements need to be granular and robust (e.g.,

For peer review only - http://bmjopen.bmj.com/site/about/guidelines.xhtml

including intended and unintended consequences), these studies are critical to developing recommendations.

Overall, the evidence synthesized by our review indicates that the PHCP-led triage interventions significantly decrease PIA or ED LOS compared to the traditional nurse-led triage model. The studies in this review demonstrate promise to improve ED patient flow metrics by either seeing and treating non-urgent patients in the triage area, starting investigations at triage for moderate to low acuity patients, or assessing and making decision to re-direct very low-acuity patients to an adjoining GP clinic with same day appointments. All of these could mitigate ED overcrowding. Since ED wait times are multi-factorial it cannot be expected that one solution will solve such a complex problem. Each ED will need an individualized approach. Moreover, while calling for improved research quality, we believe comparative effectiveness studies with health economic outcomes are required to fully weigh the costs and benefits associated with any intervention.

Strengths and Limitations

We acknowledge the following limitations in interpreting the results of this systematic review. All systematic reviews are susceptible to publication and selection bias. Selection bias was minimized by using a comprehensive, peer-reviewed search strategy developed by an experienced information specialist. Selection bias was also addressed by using two independent reviewers and third-party adjudication. We evaluated the quality of each included study using the NICE Quality Appraisal Tool[68]; that is tailored to quantitative studies investigating public health interventions. A few included studies reported the effectiveness of GP team triage intervention on the review outcomes, thus limiting conclusions on GP-led triage interventions. Most of the included studies were of pre-post intervention design providing low quality evidence. Even the

For peer review only - http://bmjopen.bmj.com/site/about/guidelines.xhtml

For peer review only

included RCT's were only of moderate quality, thus evidence from high quality studies is lacking, limiting the confidence that can be placed on the results. Nevertheless, irrespective of the study design, we observed a significant decrease in ED LOS favoring PHCP-led triage intervention. Although we used a comprehensive search strategy, for the sake of feasibility we did not consider non-English language studies and the possibility of missing some of the other language studies remains. Despite the compressive search strategy, publication bias is likely since many operational studies never reach publication and many of those would be negative. We also encountered issues with missing data in some of the included studies and resorted to imputation techniques as we were unable to obtain data from study authors. The included studies in this systematic review did not focus on clinical outcomes, such as delayed or missed diagnosis, but it would be important for future studies to quantify relevant clinical outcomes. As included studies were conducted in various countries, health systems and societal contexts, the results from one may not be compatible with evidence from other jurisdictions.

Notwithstanding the above concerns, we believe this review has many strengths, including the rigorous Cochrane systematic review methodology employed and the use of an *a priori* registered protocol. In addition, our study team included patient partners who collaborated with the investigators during the design, conduct and dissemination phases of the study. Following the criteria identified for patient-oriented research which emphasizes the active and meaningful engagement of patients as research partners, twelve diverse group of patient partners from three Canadian provinces (Manitoba, Alberta, and Quebec) were engaged from the design stage and throughout the research process around decisions and in knowledge dissemination.

CONCLUSIONS

For peer review only - http://bmjopen.bmj.com/site/about/guidelines.xhtml

PHCP-led triage interventions could be an effective strategy to improve ED patient flow overall by decreasing ED LOS, PIA, time to triage or ED visits, and by improving patient satisfaction. While these triage interventions may work in specific settings, each ED is unique, and policy would have to be evaluated specific to that facility and system. High quality methods are also necessary to further support PHCP's role in ED triage, and it is important for future studies to focus on cost efficiency or incremental value for money as these are critical real-world issues. Additionally, future research could focus on generating high quality evidence on the effectiveness of GP triage intervention. The acceptability of a PHCP-led interventions in an ED could also be formally ascertained in future studies as experience and beliefs of ED staff may play a role in the success or failure of the policy to implement PHCPs in triage. Finally, the research gap involving rural EDs needs to be addressed.

Acknowledgements

We are very grateful for the Canadian Institutes of Health Research (CIHR) grant (NKS 158643) and the Manitoba Medical Services Foundation (#8-2018-06) and Winnipeg Foundation (#8-2018-06) for providing financial support for this project. Dr. Rowe's research was supported by a Scientific Director's Grant (SOP 168483) from CIHR. Dr. Tricco's research is funded by a Tier 2 Canada Research Chair in Knowledge Synthesis. We are also very grateful to all the patient partners who collaborated with us throughout the duration of this project. We thank Dr. Cristina Villa-Roel for her support during grant and manuscript preparation and the Emergency Medicine Research Group (EMeRG) in the Department of Emergency Medicine at the University of Alberta for in-kind resources. We are very grateful to all the patient partners who collaborated with us in the design, conduct and dissemination stages of this project. We are thankful to Frank

For peer review only - http://bmjopen.bmj.com/site/about/guidelines.xhtml

Krupka for the kind support of this project during his time as the Executive Director of CHI. We thank Tamara Rader, Christine Nielson, and Janet Joyce for help with the search strategy for this study. We thank our partner organizations (Strategy for Patient-Oriented Research Support for People and Patient-Oriented Research and Trials units [Manitoba, Alberta and Quebec], EMeRG, and Network in Primary and Integrated Healthcare Innovations), and the knowledge users (Winnipeg Regional Health Authority, Emergency Strategic Clinical Network, Shared Health Manitoba, and Manitoba College of Family Physicians) for their kind support and feedback throughout the duration of this project.

Funding: This work was supported by research grant from Canadian Institutes of Health Research (NKS 158643), Manitoba Medical Services Foundation (# 8-2018-06) and Winnipeg Foundation (# 8-2018-06).

Competing interests: None declared.

Data sharing statement: All data relevant to the study are included in the article or uploaded as supplementary information.

Ethics Approval: Not applicable. This study is a systematic review and did not involve human participants.

Author contributions: MJ, AMAS, TB and MH contributed to the design and conception of the study. MJ drafted the manuscript with feedback from co-authors. MJ, AMAS and RR contributed to the data analysis. NA contributed to developing the search strategy. LC, RA, and NAY were involved in study selection process and in data extraction. MD, SB, JM, PT, BHR, AC, WS brought content expertise in emergency medicine. RS, GH, TB, and JE brought content expertise in family medicine. CS brought content expertise in patient engagement. ACT, RZ, MJ, and

For peer review only - http://bmjopen.bmj.com/site/about/guidelines.xhtml

AMAS brought content expertise in systematic review methodology. All study authors approved the final manuscript.

For peer review only - http://bmjopen.bmj.com/site/about/guidelines.xhtml

Abbreviations:

ATS: Australian triage scale

COPD: Chronic obstructive pulmonary disease

CTAS: Canadian triage and acuity scale

CBA: Controlled before and after

ED: Emergency department

ESI: Emergency Severity Index

GP: General practitioner

LAMA: Leaving against medical advice

LOS: Length of stay

LWBS: Leaving without being seen

NICE: National Institute for Health and Care Excellence

NP: Nurse practitioner

PHCP: Primary healthcare provider

PRISMA: Preferred Reporting Items for Systematic reviews and Meta-Analyses

RCT: Randomized controlled trial

TT: Time to triage

PIA: Time to provider initial assessment

For peer review only

For peer review only - http://bmjopen.bmj.com/site/about/guidelines.xhtml

References

1. Hsia RY, Kellermann AL, Shen Y-C. Factors Associated With Closures of Emergency Departments in the United States. *JAMA.* 2011;305(19):1978-1985.
2. Pitts SR, Pines JM, Handrigan MT, Kellermann AL. National trends in emergency department occupancy, 2001 to 2008: effect of inpatient admissions versus emergency department practice intensity. *Annals of emergency medicine.* 2012;60(6):679-686. e673.
3. Schull MJ, Szalai JP, Schwartz B, Redelmeier DA. Emergency department overcrowding following systematic hospital restructuring trends at twenty hospitals over ten years. *Academic Emergency Medicine.* 2001;8(11):1037-1043.
4. Skinner H, Blanchard J, Elixhauser A. Trends in emergency department visits, 2006–2011: Statistical brief# 179. 2006.
5. Trzeciak S, Rivers EP. Emergency department overcrowding in the United States: an emerging threat to patient safety and public health. *Emergency Medicine Journal.* 2003;20(5):402-405.
6. Pines JM, Hollander JE. Emergency department crowding is associated with poor care for patients with severe pain. *Annals of emergency medicine.* 2008;51(1):1-5.
7. Richardson DB. Increase in patient mortality at 10 days associated with emergency department overcrowding. *Medical journal of Australia.* 2006;184(5):213-216.
8. Oredsson S, Jonsson H, Rognes J, et al. A systematic review of triage-related interventions to improve patient flow in emergency departments. *Scandinavian journal of trauma, resuscitation and emergency medicine.* 2011;19(1):43.
9. Morley C, Unwin M, Peterson GM, Stankovich J, Kinsman L. Emergency department crowding: A systematic review of causes, consequences and solutions. *PloS one.* 2018;13(8):e0203316.
10. Asplin BR, Magid DJ, Rhodes KV, Solberg LI, Lurie N, Camargo CA, Jr. A conceptual model of emergency department crowding. *Ann Emerg Med.* 2003;42(2):173-180.
11. Fatovich DM, Hirsch RL. Entry overload, emergency department overcrowding, and ambulance bypass. *Emerg Med J.* 2003;20(5):406-409.
12. Platter MEM, Kurvers RAJ, Janssen L, Verweij MMJ, Barten DG. The impact of an emergency care access point on pediatric attendances at the emergency department: An observational study. *The American journal of emergency medicine.* 2019.
13. Primary Care Provider. https://www.healthcare.gov/glossary/primary-care-provider/.
14. Uscher-Pines L, Pines J, Kellermann A, Gillen E, Mehrotra A. Deciding to visit the emergency department for non-urgent conditions: a systematic review of the literature. *The American journal of managed care.* 2013;19(1):47.
15. Morgan SR, Smith MA, Pitts SR, et al. Measuring value for low-acuity care across settings. *Am J Manag Care.* 2012;18(9):e356-363.
16. Sancton K, Sloss L, Berkowitz J, Strydom N, McCracken R. Low-acuity presentations to the emergency department: Reasons for and access to other health care providers before presentation. *Can Fam Physician.* 2018;64(8):e354-e360.
17. Uscher-Pines L, Pines J, Kellermann A, Gillen E, Mehrotra A. Emergency department visits for nonurgent conditions: systematic literature review. *Am J Manag Care.* 2013;19(1):47-59.
18. Hayden C, Burlingame P, Thompson H, Sabol VK. Improving patient flow in the emergency department by placing a family nurse practitioner in triage: a quality-improvement project. *Journal of Emergency Nursing.* 2014;40(4):346-351.
19. Tsai VW, Sharieff GQ, Kanegaye JT, Carlson LA, Harley J. Rapid medical assessment: improving pediatric emergency department time to provider, length of stay, and left without being seen rates. *Pediatric emergency care.* 2012;28(4):354-356.

For peer review only - http://bmjopen.bmj.com/site/about/guidelines.xhtml

20. Uthman OA, Walker C, Lahiri S, et al. General practitioners providing non-urgent care in emergency department: a natural experiment. *BMJ Open.* 2018;8(5):e019736.
21. Mortensen K. Copayments did not reduce medicaid enrollees' nonemergency use of emergency departments. *Health Aff (Millwood).* 2010;29(9):1643-1650.
22. Ismail SA, Gibbons DC, Gnani S. Reducing inappropriate accident and emergency department attendances:: a systematic review of primary care service interventions. *Br J Gen Pract.* 2013;63(617):e813-e820.
23. Kirkland SW, Soleimani A, Rowe BH, Newton AS. A systematic review examining the impact of redirecting low-acuity patients seeking emergency department care: is the juice worth the squeeze? *Emerg Med J.* 2019;36(2):97-106.
24. Rose KD, Ross JS, Horwitz LI. Advanced access scheduling outcomes: a systematic review. *Arch Intern Med.* 2011;171(13):1150-1159.
25. Degani N. Impact of advanced (open) access scheduling on patients with chronic diseases: an evidence-based analysis. Ont Health Technol Assess Ser [Internet]. 2013 September;13(7):1–48. Available from: https://www.hqontario.ca/Portals/0/Documents/evidence/reports/full-report-advanced-access-130911-en.pdf.
26. Love RA, Murphy JA, Lietz TE, Jordan KS. The effectiveness of a provider in triage in the emergency department: a quality improvement initiative to improve patient flow. *Advanced Emergency Nursing Journal.* 2012;34(1):65-74.
27. Rowe BH, Guo X, Villa-Roel C, et al. The role of triage liaison physicians on mitigating overcrowding in emergency departments: a systematic review. *Academic Emergency Medicine.* 2011;18(2):111-120.
28. Kool RB, Homberg DJ, Kamphuis HC. Towards integration of general practitioner posts and accident and emergency departments: a case study of two integrated emergency posts in the Netherlands. *BMC Health Services Research.* 2008;8:225.
29. van den Bersselaar DLCM, Maas M, Thijssen WAMH. Does X-ray imaging by GPC at emergency care access points in the Netherlands change patient flow and reduce ED crowding? A cohort study. *Health science reports.* 2018;1(2):e26.
30. van Gils-van Rooij ESJ, Meijboom BR, Broekman SM, Yzermans CJ, de Bakker DH. Is patient flow more efficient in Urgent Care Collaborations? *European Journal of Emergency Medicine.* 2018;25(1):58-64.
31. Celona CA, Amaranto A, Ferrer R, et al. Interdisciplinary Design to Improve Fast Track in the Emergency Department. *Advanced Emergency Nursing Journal.* 2018;40(3):198-203.
32. Day TE, Al-Roubaie AR, Goldlust EJ. Decreased length of stay after addition of healthcare provider in emergency department triage: a comparison between computer-simulated and real-world interventions. *Emerg Med J.* 2013;30(2):134-138.
33. Edwards T. How rapid assessment at triage can improve care outcomes. *Emergency Nurse.* 2011;19(6):27-30.
34. Gardner RM, Friedman NA, Carlson M, Bradham TS, Barrett TW. Impact of revised triage to improve throughput in an ED with limited traditional fast track population. *American Journal of Emergency Medicine.* 2018;36(1):124-127.
35. MacKenzie RS, Burmeister DB, Brown JA, et al. Implementation of a rapid assessment unit (intake team): impact on ED length of stay. *Am J Emerg Med.* 2015;33(2):291-293.
36. Pierce BA, Gormley D. Are Split Flow and Provider in Triage Models in the Emergency Department Effective in Reducing Discharge Length of Stay? *Journal of Emergency Nursing.* 2016;42(6):487-491.
37. Rogers T, Ross N, Spooner D. Evaluation of a 'see and treat' pilot study introduced to an emergency department. *Accident and Emergency Nursing.* 2004;12(1):24-27.

For peer review only - http://bmjopen.bmj.com/site/about/guidelines.xhtml

38. Shrimpling M. Redesigning triage to reduce waiting times. *Emerg Nurse.* 2002;10(2):34-37.
39. Tucker A, Bernard M. Making the Case for Nurse Practitioners in the Emergency Department: A Clinical Case Study. *Advanced Emergency Nursing Journal.* 2015;37(4):308-312.
40. Zager K, Taylor YJ. Discharge to medical home: A new care delivery model to treat non-urgent cases in a rural emergency department. *Healthcare.* 2018;21:21.
41. Cheung WWH, Heeney L, Pound JL. An advance triage system. *Accident & Emergency Nursing.* 2002;10(1):10-16.
42. Lee WW, Filiatrault L, Abu-Laban RB, Rashidi A, Yau L, Liu N. Effect of Triage Nurse Initiated Radiography Using the Ottawa Ankle Rules on Emergency Department Length of Stay at a Tertiary Centre. *CJEM.* 2016;18(2):90-97.
43. Lindley-Jones M, Finlayson BJ. Triage nurse requested x rays--are they worthwhile? *J Accid Emerg Med.* 2000;17(2):103-107.
44. Parris W, McCarthy S, Kelly AM, Richardson S. Do triage nurse-initiated X-rays for limb injuries reduce patient transit time? *Accid Emerg Nurs.* 1997;5(1):14-15.
45. Thurston J, Field S. Should accident and emergency nurses request radiographs? Results of a multicentre evaluation. *J Accid Emerg Med.* 1996;13(2):86-89.
46. Adam H, Tamim H, Altamimi S, et al. Effect of triage nurse ordered distal extremity X-rays on emergency department length of stay: A randomized controlled trial. *Academic Emergency Medicine.* 2014;21(5 SUPPL. 1):S195.
47. Al Abri FH, Muliira JK, Al Awaisi H. Effect of triage nurse-led application of the Ottawa Ankle Rules on number of radiographic tests and length of stay in selected emergency departments in Oman. *Japan journal of nursing science : JJNS.* 2020;17(1):e12270.
48. Al Kadhi O, Manley K, Natarajan M, et al. A renal colic fast track pathway to improve waiting times and outcomes for patients presenting to the emergency department. *Open access emergency medicine : OAEM.* 2017;9:53-55.
49. Ashurst JV, Nappe T, Digiambattista S, et al. Effect of triage-based use of the Ottawa foot and ankle rules on the number of orders for radiographic imaging. *The Journal of the American Osteopathic Association.* 2014;114(12):890-897.
50. Demarco F, Gerardo CJ, Boardwine A, et al. Effect of a nurse rapid intake initiative on patient length of stay and satisfaction: A project IMPACT initiative. *Academic Emergency Medicine.* 2010;17(SUPPL. 1):S93-S94.
51. Dixon A, Clarkin C, Barrowman N, Correll R, Osmond MH, Plint AC. Reduction of radial-head subluxation in children by triage nurses in the emergency department: A cluster-randomized controlled trial. *CMAJ.* 2014;186(9):E317-E323.
52. Fan J, Woolfrey K. The effect of triage-applied Ottawa Ankle Rules on the length of stay in a Canadian urgent care department: a randomized controlled trial. *Academic emergency medicine : official journal of the Society for Academic Emergency Medicine.* 2006;13(2):153-157.
53. Fontanel A, Besson C, Gallegos C, Vallot C, Droal D. Can X-rays be prescribed by a nurse in an emergency department? *European Journal of Emergency Medicine.* 2011;18(5):310-311.
54. Gaucher N, Bailey B, Gravel J. Triage nurses' counselling influences return visits of children leaving the emergency department before being seen by a physician. *Academic Emergency Medicine.* 2010;17(SUPPL. 1):S118-S119.
55. Hackman JL, Roth ED, Gaddis ML, Gratton MC. The effect of a nurse-initiated chest pain protocol on disposition time: A retrospective review. *Annals of Emergency Medicine.* 2015;66(4 SUPPL. 1):S9-S10.
56. Ho JK-M, Chau JP-C, Chan JT-S, Yau CH-Y. Nurse-initiated radiographic-test protocol for ankle injuries: A randomized controlled trial. *International emergency nursing.* 2018;41:1-6.

For peer review only - http://bmjopen.bmj.com/site/about/guidelines.xhtml

57. Jobé J, Vandercleyen C, Ghuysen A, D'Orio V. Prospective study of an advanced nurse triage for a target pathology at the admission in the emergency department. *Acta clinica belgica.* 2013;68(6):482-.
58. Klassen TP, Ropp LJ, Sutcliffe T, et al. A randomized, controlled trial of radiograph ordering for extremity trauma in a pediatric emergency department. *Annals of emergency medicine.* 1993;22(10):1524-1529.
59. Lee KM, Wong TW, Chan R, Lau CC, Fu YK, Fung KH. Accuracy and efficiency of X-ray requests initiated by triage nurses in an accident and emergency department. *Accident and emergency nursing.* 1996;4(4):179-181.
60. Lee WW, Filiatrault L, Abu-Laban RB, Rashidi A, Yau L, Liu N. Effect of triage nurse initiated radiography using the Ottawa Ankle Rules on emergency department length of stay at a tertiary care center. *Canadian Journal of Emergency Medicine.* 2014;16(SUPPL. 1):S38.
61. Li Y, Lu Q, Du H, Zhang J, Zhang L. The Impact of Triage Nurse-ordered Diagnostic Studies on Pediatric Emergency Department Length of Stay. *Indian journal of pediatrics.* 2018;85(10):849-854.
62. Lijuan Z. Advanced Triage Protocols in the Emergency Department. *Advanced Triage Protocols in the Emergency Department.* 2017:1-1.
63. Sikkenga T, Dumkow L, Draper H, et al. Implementation of a nursing triage order to improve utilization of rapid diagnostic testing for chlamydia and gonorrhea in the emergency department. *Open Forum Infectious Diseases.* 2016;3(Supplement 1).
64. Nash K, Nguyen H, Tillman M. Using medical screening examinations to reduce emergency department overcrowding. *Journal of Emergency Nursing.* 2009;35(2):109-113.
65. Organ K, Chinnick P, Higgison I, Stanhope B, Hoskins R, Benger J. Evaluating the introduction of a paediatric emergency nurse practitioner service. *Emergency nurse : the journal of the RCN Accident and Emergency Nursing Association.* 2005;13(7):8-11.
66. Liberati A, Altman DG, Tetzlaff J, et al. The PRISMA statement for reporting systematic reviews and meta-analyses of studies that evaluate health care interventions: explanation and elaboration. *PLoS medicine.* 2009;6(7):e1000100.
67. McGowan J, Sampson M, Salzwedel D, Cogo E, Foerster V, Lefebvre C. PRESS Peer Review of Electronic Search Strategies: 2015 Guideline Statement. *Journal of clinical epidemiology.*75:40-46.
68. Appendix F Quality appraisal checklist-quantitative intervention studies. 2012; https://www.nice.org.uk/process/pmg4/chapter/appendix-f-quality-appraisal-checklist-quantitative-intervention-studies. Accessed July 24, 2019, 2019.
69. Staniszewska S, Brett J, Simera I, et al. GRIPP2 reporting checklists: tools to improve reporting of patient and public involvement in research. *BMJ.* 2017;358:j3453.
70. MacKenzie RS, Burmeister DB, Brown JA, et al. Implementation of a rapid assessment unit (intake team): impact on ED length of stay. *American Journal of Emergency Medicine.* 2015;33(2):291-293.
71. Tsai VW, Sharieff GQ, Kanegaye JT, Carlson LA, Harley J. Rapid medical assessment: improving pediatric emergency department time to provider, length of stay, and left without being seen rates. *Pediatric Emergency Care.* 2012;28(4):354-356.
72. Rowe BH, Villa-Roel C, Guo X, et al. The role of triage nurse ordering on mitigating overcrowding in emergency departments: a systematic review. *Acad Emerg Med.* 2011;18(12):1349-1357.
73. Jennings N, Clifford S, Fox AR, O’Connell J, Gardner G. The impact of nurse practitioner services on cost, quality of care, satisfaction and waiting times in the emergency department: a systematic review. *International journal of nursing studies.* 2015;52(1):421-435.

For peer review only - http://bmjopen.bmj.com/site/about/guidelines.xhtml

74. Gonçalves-Bradley D, Khangura JK, Flodgren G, Perera R, Rowe BH, Shepperd S. Primary care professionals providing non-urgent care in hospital emergency departments. *Cochrane Database of Systematic Reviews.* 2018(2).

For peer review only

For peer review only - http://bmjopen.bmj.com/site/about/guidelines.xhtml

Table 1: Characteristics of included studies

Study ID (First Author, Year)	Country; Urban/rural ED; Adult/Pediatric/mixed ED	Study Design	Type of PHCP	Intervention	Duration of intervention (months)	Study Quality
Adam, 2014	Saudi Arabia; Urban ED; Pediatric ED	RCT	Nurse	Nurse triage-plus	0.5	Moderate
Al Abri, 2020	Oman; Urban ED; Adult ED	CBA	Nurse	Nurse triage-plus	NR	Low
Al Khadi, 2017	UK; NR; NR	RC study	Nurse	Nurse triage-plus	12	Low
Ashurst, 2014	USA; Urban ED; Mixed ED	PC study	Nurse	Nurse triage-plus	10	Low
Celona, 2018	USA; Urban ED; Adult ED	Pre-post	NP	NP Team triage	12	Low
Cheung, 2002	Canada; Urban ED; NR	RC study	Nurse	Nurse triage-plus	NR	Low
Day, 2013	USA; Urban ED; NR	Pre-post	NP	NP Team triage	1	Low
Demarco, 2010	NR; Urban ED; NR	PC study	Nurse	Nurse triage-plus	1	Low
Dixon, 2014	Canada; Urban ED; Pediatric ED	RCT	Nurse	Nurse triage-plus	12	Low
Edwards, 2011	Australia; Urban ED; Mixed ED	Pre-post	NP	NP Team triage	17	Low
Fan, 2006	Canada; Urban ED; NR	RCT	Nurse	Nurse triage-plus	3	Moderate
Fontanel, 2011	France; NR; Mixed ED	CBA	Nurse	Nurse triage-plus	0.08	Low
Gardner, 2018	USA; Urban ED; Adult ED	Pre-post	NP	NP Team triage	0.5	Low
Gaucher, 2010	Canada; NR; Pediatric ED	RC study	Nurse	Nurse triage-plus	12	Low
Hackman, 2015	USA; Urban ED; NR	RC study	Nurse	Nurse triage-plus	17	Low
Hayden, 2014	USA; NR; Mixed ED	Pre-post	NP	NP Team triage	2	Low
Ho, 2018	China; NR; Mixed ED	RCT	Nurse	Nurse triage-plus	NR	Moderate
Jobe, 2019	France; NR; NR	RCT	Nurse	Nurse triage-plus	NR	Low
Klassen, 1993	Canada; Urban ED; Pediatric ED	RCT	Nurse	Nurse triage-plus	12	Low
Kool, 2008	Netherlands; Both; NR	CBA	GP	GP team triage	12	Low
Lee, 1996	China; Urban ED; Mixed ED	PC study	Nurse	Nurse triage-plus	3	Low
Lee, 2014	Canada, Urban ED; NR	RCT	Nurse	Nurse triage-plus	12	Moderate
Lee, 2016	Canada; Urban ED; Adult ED	RCT	Nurse	Nurse triage-plus	12	Moderate
Li, 2018	China; NR; Pediatric ED	RC study	Nurse	Nurse triage-plus	5	Low
Lijuan, 2017	USA; NR; NR	Pre-post	Nurse	Nurse triage-plus	6	Low
Lindley Jones, 2000	England; Urban ED; NR	RCT	Nurse	Nurse triage-plus	12	Moderate

For peer review only - http://bmjopen.bmj.com/site/about/guidelines.xhtml

Love, 2012	USA; Urban ED; NR	Pre-post	NP	NP Team triage	0.5	Low
MacKenzie, 2015	USA; NR; NR	Pre-post	NP	NP Team triage	2	Low
Parris, 1997	Australia; Urban ED; Adult ED	Quasi-RCT	Nurse	Nurse triage-plus	6	Low
Pierce, 2016	USA; Urban ED; NR	CBA	NP	NP Team triage	5.5	Low
Rogers, 2004	England; Urban ED; NR	Pre-post	NP	NP Team triage	12	Low
Shrimpling, 2002	England; Urban ED; NR	Pre-post	NP	NP Team triage	0.75	Low
Sikkenga, 2016	USA; Urban ED; NR	RC study	Nurse	Nurse triage-plus	NR	Low
Thurston, 1996	England; Urban ED; NR	RCT	Nurse	Nurse triage-plus	2	Moderate
Tsai, 2012	USA; Urban ED; Pediatric ED	Pre-post	NP	NP Team triage	NR	Low
Tucker, 2015	USA; Urban ED; NR	Pre-post	NP	NP Team triage	6	Low
Uthman, 2018	England; Urban ED; NR	RC study	NP	NP Team triage	12	Low
van den Bersselaar, 2018	Netherlands; Urban ED; NR	RC study	GP	GP team triage	11	Low
van Gils-van Rooij, 2018	Netherlands; Both; NR	CS study	GP	GP team triage	NA	Low
Zager, 2018	USA; Rural ED; NR	Pre-post	NP	NP Team triage	4	Low

CS study: Cross-sectional observational study; RC study: Retrospective cohort study; ED: Emergency department; PHCP: Primary healthcare provider; CBA: Controlled before and after study; RCT: Randomized controlled trial; GP: General practitioner; NP: Nurse practitioner; NR: Not reported; NA: Not applicable

For peer review only - http://bmjopen.bmj.com/site/about/guidelines.xhtml

Table 2: Leave without being seen (LWBS) outcome data reported by included studies

Study ID (First Author, Year)	Triage Intervention	Study Design	Intervention (%)	Comparator (%)	Percentage Difference	Reported Statistical Significance
Celona, 2018	NP team triage	Pre-post	4.7	3.3	1.4	NR
Love, 2012	NP team triage	Pre-post	0.93	3.39	-2.46	Significant
MacKenzie, 2015	NP team triage	Pre-post	0.7333	2.96	-2.2267	Significant
Gardner, 2017	NP team triage	Pre-post	2.2	4.6	-2.4	Significant
Hayden, 2014	NP team triage	Pre-post	5.8	5.4	0.4	NS
Tsai, 2012	NP team triage	Pre-post	3	9.7	-6.7	Significant
Tucker, 2015	NP team triage	Pre-post	1.3	5.07	-3.77	NR
Uthman, 2018	NP team triage	Retrospective cohort	2.2	3.9	-1.7	Significant
Li, 2018	Nurse triage-plus	Retrospective cohort	0.7	6.9	-6.2	NS
Lijuan, 2017	Nurse triage-plus	Pre-post	7.13	7.52	-0.39	NS

NP: Nurse practitioner; NR: Not reported; NS: Not significant

For peer review only

For peer review only - http://bmjopen.bmj.com/site/about/guidelines.xhtml

Table 3: Leave Against Medical Advice (LAMA) outcome data reported by included studies

Study ID (First Author, Year)	Triage Intervention	Study Design	Intervention (%)	Comparator (%)	Percentage Difference	Reported Statistical Significance
MacKenzie, 2015	NP team triage	Pre-post	0.22	0.33	-0.11	NS
Tucker, 2015	NP team triage	Pre-post	1.41	1.29	0.12	NS
Hayden, 2014	NP team triage	Pre-post	1.4	0.06	1.34	NR

NP: Nurse practitioner; NR: Not reported; NS: Not significant

For peer review only

For peer review only - http://bmjopen.bmj.com/site/about/guidelines.xhtml

Figure legends

Figure 1: PRISMA study flow diagram

Figure 2: Various models for PHCP involvement in triage of emergency department patients.

Figure 3: Effectiveness of PHCP interventions on time to provider initial assessment (in minutes) sub-grouped by study design. The horizontal black lines represent 95% confidence intervals and the red dots in the middle represents point estimates (mean difference).

Figure 4: Effectiveness of PHCP interventions on ED LOS (in minutes) sub-grouped by study design. The horizontal black lines represent 95% confidence intervals and the red dots in the middle represents point estimates (mean difference).

For peer review only

For peer review only - http://bmjopen.bmj.com/site/about/guidelines.xhtml

Figure 1: PRISMA study flow diagram

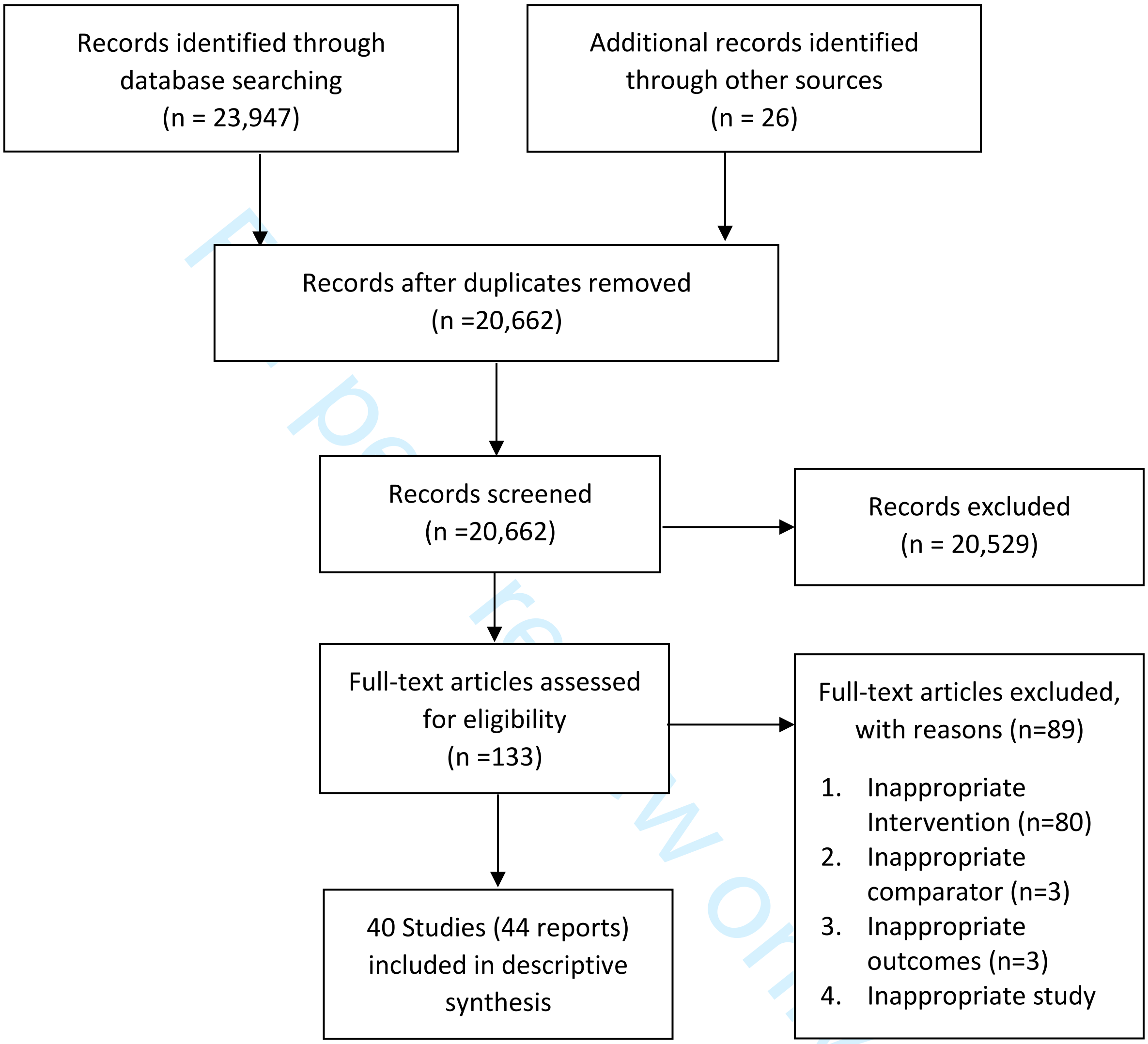

For peer review only - http://bmjopen.bmj.com/site/about/guidelines.xhtml

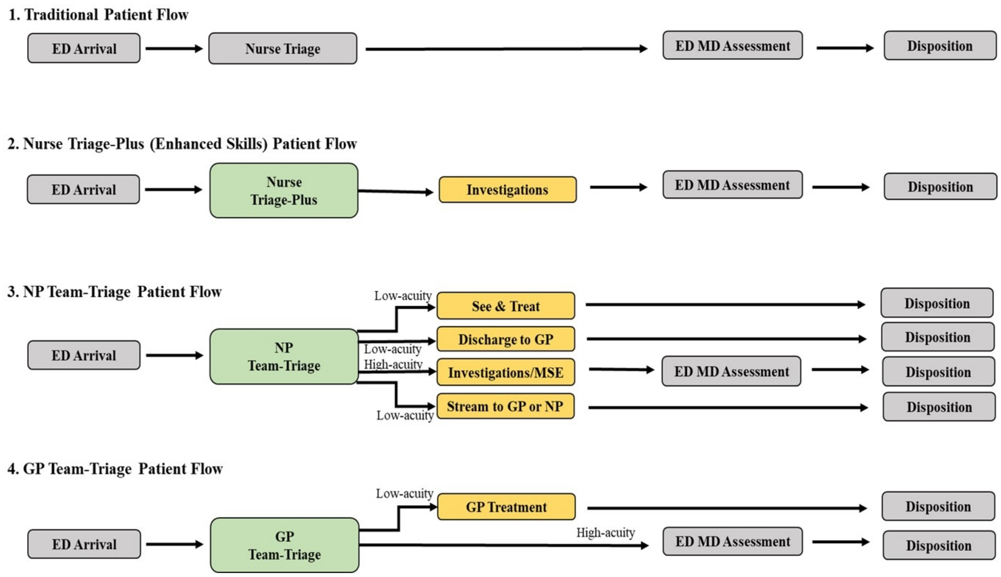

Various models for PHCP involvement in triage of emergency department patients

228x131mm (150 x 150 DPI)

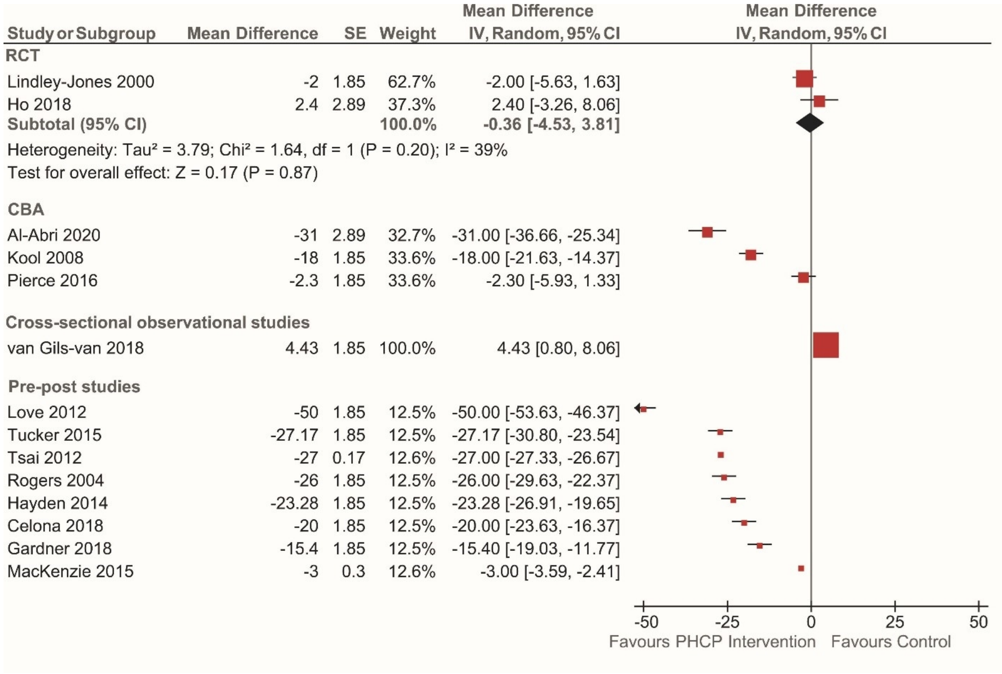

Effectiveness of PHCP interventions on time to provider initial assessment (in minutes) sub-grouped by study design

161x108mm (220 x 220 DPI)

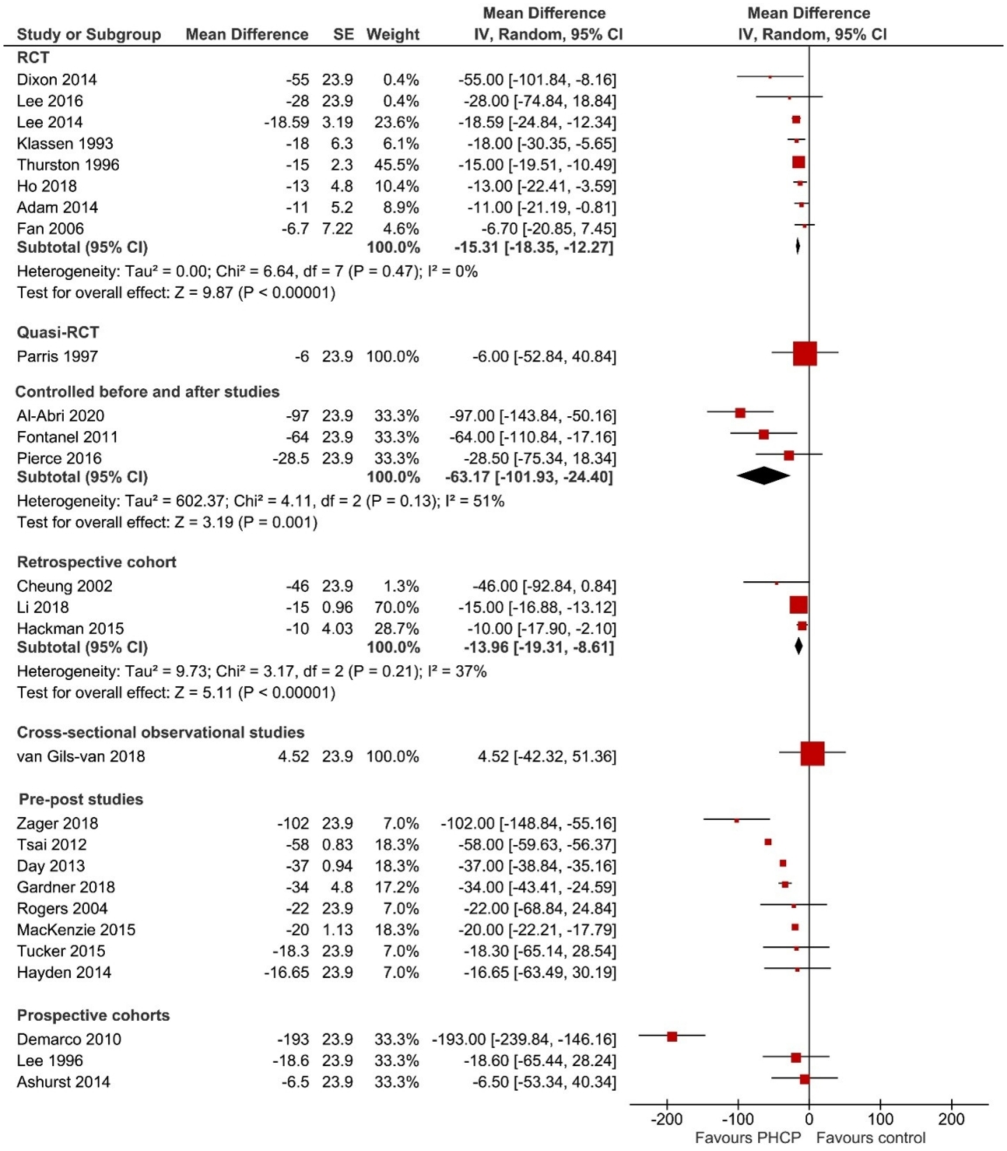

Effectiveness of PHCP interventions on ED LOS (in minutes) sub-grouped by study design

165x189mm (220 x 220 DPI)

Appendix Methods:

The National Institute for Health and Care Excellence (NICE) quality appraisal tool has four domains: characteristics of the population, allocation methods, outcomes, and analyses. Each domain has multiple questions for which there are five response options: (1) study has been conducted in such a way to minimize the risk of bias (++), (2) study has not addressed all potential sources of bias (+), (3) significant sources of bias persists in the study (-), (4) not reported or (5) not applicable. A fifth domain summarizes the overall quality of the included study based on the assessments of the four domains. The overall quality of each included study was assessed as either low quality (-), moderate quality (+) or high quality (++), based on adjudications made on the four individual domains for that study.

Appendix Results:

1. ***Patient triage acuity rating***: Various scales such as Emergency Severity Index (ESI), Australasian Triage Scale (ATS), the Canadian Triage or Acuity Scale (CTAS) or triage acuity rating scale were used to assess the acuity levels patients arriving at the ED. Six included studies[1-6] (15%) reported triaging patients who were triage category 3-5 (one study involved <10% of category 3 patients, two studies[2,7] involved >20% of category 3 patients, and three studies[1,4,5] did not report the percentage of patients in each category 3-5). Five included studies[8-12] (10%), reported triaging both low and high acuity patients (with approximately 90% of category 3-5 patients). Fourteen (35%) studies[13-26] did not report acuity levels of patients.

2. ***Time to physician initial assessment (PIA) sub-grouped by various PHCP interventions:***

   Of the 14 studies, the majority[18,26,32,42,48,61,63,66,67] (n = 9) reported the effect of NP team triage on PIA, and the rest reported either the effect of GP team triage[54,69] (n = 2) or nurse

For peer review only

triage-plus[39,51,60] (n=3) on PIA, respectively. All studies in NP team triage group showed a decrease in PIA (median [range]= -21.7 minutes [-2.3 to -50]) favoring the intervention group. In the nurse triage-plus group all except one[51] showed a decrease in PIA (median [range]= -2.4 minutes [-2 to -31]), Among the two studies[54,69] in GP team triage group, one prospective CBA interventional study[54] reported statistically significant decrease (-18 minutes) in PIA favoring the intervention group. Whereas the second cross-sectional observational study[69] showed an increase (4.43 minutes) in PIA (reported as statistically significant), favoring the traditional nurse-led triage model.

3. ***Emergency department length of stay (ED LOS) sub-grouped by various PHCP interventions:***

Twenty studies[7,8,13-19,21-31] reported on the effect of nurse triage-plus on ED LOS, nine studies[1,2,4-6,10,32-34] reported on the effect of NP team triage on ED LOS, and one study[11] on the effect of GP team triage on ED LOS. Seventeen studies[7,8,13,15-19,21,23-25,27-31] in the nurse triage-plus model reported a decrease (median = -18 minutes) in ED LOS favoring the intervention group. All nine studies[1,2,4-6,10,32-34] in the NP team triage model showed a decrease (median = -28.50 minutes) in ED LOS favoring the intervention group. One study in the GP team triage model did not show any significant difference in ED LOS between comparison groups.

Four studies reported percentage of patients discharged within benchmark times (ED specific). Rogers et al. reported 41% of patients discharged from the ED within one hour in the NP team triage group compared to only 16% patients discharged within one hour in the traditional nurse-led triage group. Tsai et al.[35] reported that 30% of low-acuity patients in the NP team triage group discharged in 90 minutes compared to 12% in the

For peer review only

traditional nurse-led triage group. Day et al.[1] reported that 85.7% of patients discharged under 6 hours in the NP team triage group compared to 80.1% in the traditional nurse-led triage group. Uthman et al.[36] reported that 98.1% of patients discharged under 4 hours in the in the NP team triage group compared to 94.7% in the traditional nurse-led triage group.

4. ***Effect of PHCP intervention on number of repeat ED visits***

Zager et al.6 reported a 5% decrease in ED visits in the NP team triage group (conducted triage, medical screening exam (MSE) and discharged low-acuity patients with a same day appointment at the GP clinic co-located with the ED) compared to the traditional nurse-led triage model (statistical significance not reported). Day et al.1 investigating NP team triage (provider at triage model) reported 2194 ED visits (over 6 weeks) during pre-intervention period compared to 1699 patient visits (over one month) during the post-intervention period (statistical significance not reported). Tucker et al.34 investigated the effect of NP team triage on ED visits and reported an increase in the number of patients visiting ED by 51 visits per month (statistical significance not reported) compared to the traditional nurse-led triage model. Bersselaar et al.37 investigated the effect of GP team triage and x-ray requests (at the emergency care access point (ECAP) in which ED and GP work together) on ED visits, and reported that 68% of patient visits were treated by the GP without ED referral leading to a reduction of 4.5% annual ED patient visits. Kool et al.38, a CBA study, investigated the effect of GP team triage at the integrated emergency post (IEP) with a joint reception for the ED and a GP clinic on ED visits compared to the control sites that are not IEP (traditional nurse-led triage model), and reported a statistically significant decrease (6257 to 5715) in the number of patient visits

at the ED at IEPs and an statistically significant increase (3985 to 4321) in the number of ED attendances at the control sites. Gaucher et al20 reported that number of return ED visits decreased from 8.1% to 6.1% in the nurse triage-plus group compared to the traditional nurse-led triage model.

5. ***Effect of PHCP intervention on patient satisfaction***

Kool et al.[37] reported no differences in patient satisfaction between patients who visited IEPs (GP team triage) compared to those who visited ED's at control sites, but patients who were phone triaged at the IEP were more satisfied (statistically significant) compared to the control sites EDs[37]. Tucker et al.[34] investigated the effect of NP team triage on ED visits and reported that patient satisfaction remained high (greater than 90%; statistical significance not reported) compared to the traditional nurse-led triage model. Gardner et al.[32] reported that with NP team triage, 62-65% of patients were more satisfied with their ED LOS, PIA and quality of care compared to traditional nurse-led triage model. Hayden et al.[2] investigated the impact of NP team triage (provider at triage model) on patient satisfaction and reported that patient satisfaction decreased slightly in the post-intervention period compared to the pre-intervention period but this decrease was not statistically significant. Five[7,12,15,16,25] studies reported an increase in patient satisfaction scores in the nurse triage-plus model compared to the traditional nurse-led triage model, whereas one[18] study reported no difference between groups.

6. ***Effect of PHCP intervention on time to triage***

One RCT[28] showed a non-significant decrease in time to triage in the nurse triage-plus group compared to traditional nurse-led triage group. Two pre-post studies[33,38] reported the effect of NP team triage on time to triage compared to the traditional nurse-led triage

model. MacKenzie et al.[38] reported statistically significant decrease (pre-intervention time to triage (Median: 4; IQR: (2, 10)); post-intervention time to triage (Median: 3; IQR: (1, 8)) favoring the intervention. Rogers et al.[33] reported that 98% percentage of patients in the NP team triage intervention group were triaged within 15 minutes compared to the comparison group (75% of patients triaged within 15 minutes).

For peer review only

Appendix Table 1: Inclusion and exclusion criteria

	**Inclusion criteria**	**Exclusion criteria**
Population	Patient population (children and adults of any age) visiting the ED	
Intervention	Any ED triage intervention or strategy involving primary healthcare providers (family physicians/general practitioner (GP), nurse practitioner (NP), or nurse given increased authority)	Studies reporting triage intervention involving emergency physicians (ED MD) or exclusively physician assistants
Comparator	Traditional nurse-led triage (standard care)	
Outcomes	*Primary outcomes*: Time to provider initial assessment	
	Secondary outcomes: ED LOS, proportion of patients that left without being seen (LWBS), ED length of stay patient satisfaction, proportion of patients leaving against medical advice (LAMA), time to triage, and number of ED visits.	
Study Design	Any comparative study design (randomized and quasi-randomized clinical trials, non-randomized controlled clinical trial/controlled before and after studies (CBA), case control studies, controlled cohort studies, interrupted time series, pre-post intervention/uncontrolled before and after studies)	Reviews, commentary, case reports, editorials, historical articles, non-human studies

For peer review only

Appendix Table 2: Medline search strategy

Database: Ovid MEDLINE(R) and Epub Ahead of Print, In-Process & Other Non-Indexed Citations and Daily <1946 to January 10, 2020>
Search Strategy:
----
1 exp primary health care/ (140156)
2 physicians, family/ (15853)
3 family practice/ (64029)
4 Physicians, Primary Care/ (2703)
5 general practice/ (11719)
6 general practitioners/ (6381)
7 (primary adj2 (care or health*)).ti,ab,kf. (129329)
8 ((general or family) adj (practice* or practitioner*)).ti,ab,kf. (84810)
9 (GP or GPs).ti,ab,kf. (51935)
10 nurse practitioners/ (16770)
11 primary care nursing/ (392)
12 family nursing/ (1349)
13 community mental health services/ (17905)
14 ((family or community or primary or ambulatory or triage) adj2 (medic* or doctor* or physician* or health* or nurs*)).ti,ab,kf. (68438)
15 Ambulatory Care/ (40524)
16 (ambulatory adj2 care).ti,ab,kf. (11413)
17 Health Services, Indigenous/ (2817)
18 Cultural Competency/ (4632)
19 Culturally Competent Care/ (830)
20 Medicine, Traditional/ (10299)
21 (trauma adj inform*).ti,ab,kf. (617)
22 (aborigin* or indigenous or native).ti,ab,kf. (225451)
23 ((after or out) adj2 hour*).ti,ab,kf. (137459)
24 or/1-23 (917850)
25 exp Emergency Service, Hospital/ (67418)
26 Emergency Medical Services/ (39232)
27 emergency treatment/ (10025)
28 Trauma centers/ (9210)
29 Triage/ (10240)
30 ((emergency or emergent or urgent) adj2 (care or healthcare or department* or unit or units or room* or treatment* or ward or service)).ti,ab,kf. (121497)
31 ("accident and emergency" or "accident & emergency" or ED or EDs or ER or A&E).ti,ab,kf. (162648)
32 (triage adj2 (centre or centres or center or centers or department? or unit or units)).ti,ab,kf. (538)
33 (emergency adj2 (care or healthcare or department? or unit or units or room? or treatment? or care or visit? or utilization or admit or admission?)).ti,ab,kf. (112731)
34 ("accident and emergency" or "accident & emergency" or emergency service?).ti,ab,kf. (10865)
35 (trauma adj2 (centre or centres or center or centers or department? or unit or units)).ti,ab,kf. (15573)
36 (triage adj2 (centre or centres or center or centers or department? or unit or units)).ti,ab,kf. (538)

37 (emergency adj2 (visit? or care or admit or admission?)).ti,ab,kf. (26760)
38 (urgent adj2 (care or healthcare or health care)).ti,ab,kf. (2099)
39 ((semiurgent or semi-urgent or nonemergen$ or non-emergen$) adj2 (treatment? or care or visit?)).ti,ab,kf. (289)
40 ((emergency or non-emergency or nonemergency or urgent or non-urgent or nonurgent or semi-urgent or semiurgent) adj2 patient?).ti,ab,kf. (11636)
41 or/25-40 (367776)
42 organizational efficiency/ (20744)
43 workflow/ (3295)
44 Waiting lists/ (10724)
45 ((wait or waiting) adj2 (time or times or list or lists)).ti. (3351)
46 ((wait or waiting or throughput or service or treatment) adj2 (time or times or list or lists) adj10 (reduce? or reduction or eliminat$ or lower or fewer or intervention or policy or policies or reform$ or effectiveness or impact or improv$ or organi?ational$ or quality or save or saving)).ab. (3119)
47 ((decrease or reduce or streamline or less or minimize or shorten or eliminate or cut or enhance or facilitate or speed or better or accelerate or optimize or reform or delay or change or faster or impact$ or assess$ or eliminat$ or improv$ or lower$ or reduc$) adj3 patient? wait$).ti,ab,kf. (303)
48 CROWDING/ (2930)
49 crowd$.ti,ab,kf. (16513)
50 congest$.ti,ab,kf. (61747)
51 overcrowd$.ti,ab,kf. (3425)
52 gridlock$.ti,ab,kf. (180)
53 queue$.ti,ab,kf. (1011)
54 overload$.ti,ab. (39413)
55 "access block$".ti,ab,kf. (166)
56 (throughput or through-put).ti,ab,kf. (87262)
57 warehous$.ti,ab,kf. (2303)
58 ("left without being seen" or "leave$ without being seen" or lwbs).ti,ab,kf. (284)
59 (patient adj2 elop$).ti,ab,kf. (16)
60 (ambulance$ adj2 diver$).ti,ab,kf. (194)
61 (ambulance$ adj2 redirect$).ti,ab,kf. (3)
62 "fast track$".ti,ab,kf. (3500)
63 delay$.ti,ab,kf. (428757)
64 ("patient flow$" or "flow of patient$").ti,ab,kf. (4939)
65 defer$.ti,ab,kf. (23198)
66 (over* adj3 (capacit$ or occupanc$)).ti,ab,kf. (4603)
67 (lama or (leave$ adj4 ("medical advice" or treatment$)) or (left adj4 ("medical advice" or treatment$))).ti,ab,kf. (8393)
68 ((hallway or corridor) adj2 (care or medicine)).ti,ab,kf. (6)
69 or/42-68 (776721)
70 24 and 41 and 69 (3799)

For peer review only

Appendix Table 3: Grey literature sources

**Grey literature sources**
BMJ Open Quality (https://bmjopenquality.bmj.com) and a Google Custom Search of the following websites:<br>Canadian Foundation for Healthcare Improvement (www.cfhi-fcass.ca), Institute for Healthcare Improvement (www.ihi.org), Agency for Healthcare Research and Quality (www.ahrq.gov), NHS Improvement (https://improvement.nhs.uk), International Society for Quality in Health Care (www.isqua.org), Health Quality Ontario (www.hqontario.ca), Saskatchewan Health Quality Council (https://hqc.sk.ca), Health Quality Council of Alberta (www.hqca.ca), BC Patient Safety & Quality Council (https://bcpsqc.ca), Australian Commission on Safety and Quality in Health Care (www.safetyandquality.gov.au), and Health Quality & Safety Commission New Zealand (www.hqsc.govt.nz).

For peer review only

Appendix Table 4: Quality assessment scores of included studies

Study ID	1.1	1.2	1.3	2.1	2.2	2.3	2.4	2.5	2.6	2.7	2.8	2.9	3.1	3.2	3.3	3.4	3.5	3.6	4.1	4.2	4.3	4.4	4.5	4.6	5.1	5.2
Celona, 2018	2+	2+	-	-	2+	-	-	2+	2+	NR	-	2+	2+	2+	2+	2+	2+	2+	NR	NR	NR	+	+	-	-	-
Cheung, 2002	2+	2+	NR	-	2+	-	NR	2+	NR	NR	-	-	2+	+	2+	2+	2+	2+	NR	NR	NR	+	+	-	-	-
Day, 2013	2+	2+	-	-	2+	-	-	2+	2+	NR	-	2+	2+	2+	2+	2+	2+	2+	NR	NR	NR	2+	+	2+	-	-
Edwards, 2011	2+	2+	2+	-	2+	-	-	+	2+	NR	-	2+	2+	2+	2+	2+	2+	2+	NR	NR	NR	2+	+	-	-	-
Gardner, 2018	+	2+	+	-	2+	-	-	2+	2+	NR	-	2+	2+	2+	2+	2+	2+	2+	NR	NR	NR	2+	+	2+	-	+
Hayden, 2014	+	+	+	-	2+	-	-	2+	2+	NR	-	2+	2+	2+	2+	2+	2+	2+	+	NR	NR	2+	2+	+	-	+
Lee, 2016	2+	+	2+	2+	2+	2+	+	2+	2+	NR	2+	2+	+	+	2+	2+	2+	2+	+	NR	2+	2+	2+	2+	+	+
Lindley Jones, 2000	2+	2+	2+	2+	2+	+	NR	2+	2+	NR	2+	+	2+	2+	2+	2+	2+	2+	NR	NR	NR	+	+	2+	+	2+
Love, 2012	2+	+	+	-	2+	-	-	2+	2+	NR	-	2+	2+	2+	2+	2+	2+	2+	NR	NR	NR	+	+	-	-	+
Mackenzie, 2015	2+	2+	2+	-	2+	-	-	2+	2+	NR	-	2+	2+	2+	2+	2+	2+	2+	-	NR	NR	2+	+	+	-	2+
Parris, 1997	2+	2+	-	-	2+	-	-	2+	2+	NR	-	2+	+	2+	2+	2+	2+	2+	NR	NR	NR	+	+	2+	-	-
Pierce, 2016	2+	+	-	-	2+	NR	-	2+	2+	NR	-	2+	2+	2+	2+	2+	2+	2+	NR	NR	NR	+	NR	NR	-	-
Rogers, 2004	2+	+	NR	-	2+	-	-	2+	2+	NR	-	2+	2+	2+	2+	2+	2+	2+	NR	NR	NR	+	-	-	-	+
Shrimpling, 2002	2+	2+	+	-	2+	-	-	2+	2+	NR	-	2+	+	2+	2+	2+	2+	2+	NR	NR	NR	+	NR	-	-	+
Thurston, 1996	2+	+	2+	+	2+	NR	NR	+	2+	NR	+	2+	+	+	2+	2+	2+	2+	NR	NR	2+	2+	+	2+	+	+

For peer review only

Tsai, 2012	2+	+	+	-	2+	-	-	2+	2+	NR	-	2+	2+	2+	2+	2+	2+	2+	NR	NR	NR	2+	2+	2+	-	+
Tucker, 2015	2+	+	+	-	2+	-	-	2+	2+	NR	-	2+	2+	2+	2+	2+	2+	2+	NR	NR	NR	+	NR	-	-	+
Uthman, 2018	2+	2+	+	-	2+	NR	NR	2+	2+	NR	+	2+	2+	2+	2+	2+	2+	2+	2+	NR	NR	2+	2+	2+	-	+
van den Bersselaar, 2018	2+	2+	+	-	2+	-	NR	2+	2+	NR	-	2+	2+	2+	2+	2+	2+	2+	NR	NR	NR	+	+	-	-	+
van Gils-van Rooij, 2018	+	2+	2+	-	2+	-	NR	2+	2+	NR	-	2+	2+	2+	2+	2+	2+	2+	+	NR	NR	+	2+	+	-	+
Zager, 2018	2+	2+	+	-	2+	-	-	2+	2+	NR	-	2+	2+	2+	2+	2+	2+	2+	NR	NR	NR	+	NR	+	-	+
Kool, 2008	2+	+	-	-	2+	-	-	2+	2+	NR	-	2+	+	-	2+	2+	2+	2+	+	NR	NR	2+	2+	+	-	-
Al Abri, 2020	2+	2+	+	-	2+	-	-	2+	NR	NR	-	2+	2+	2+	2+	2+	2+	2+	+	NR	NR	-	2+	2+	+	+
Ho, 2018	2+	2+	+	+	2+	NR	-	2+	2+	NR	2+	2+	2+	2+	2+	2+	2+	2+	2+	2+	2+	2+	2+	2+	-	+
Li, 2018	+	2+	+	-	2+	-	-	2+	NR	NR	-	2+	2+	2+	2+	2+	2+	2+	-	NR	NR	-	2+	+	-	+
Hackman, 2015	+	+	+	-	2+	-	-	2+	NR	NR	-	2+	2+	2+	2+	2+	2+	2+	-	NR	NR	2+	2+	2+	-	+
Klassen, 1993	2+	2+	+	2+	2+	+	+	2+	NR	NR	2+	2+	2+	2+	2+	2+	2+	2+	2+	-	NR	-	2+	+	-	+
Al Khadi, 2017	+	+	+	-	2+	-	-	2+	NR	NR	-	2+	2+	2+	2+	2+	2+	2+	-	NR	NR	-	2+	+	-	+
Ashurst, 2014	+	2+	+	-	2+	-	-	2+	NR	NR	-	2+	+	2+	2+	2+	2+	2+	-	2+	2+	-	2+	+	-	+
Fan, 2006	+	2+	+	2+	2+	+	+	2+	2+	NR	2+	+	+	+	2+	2+	2+	2+	2+	2+	2+	2+	2+	2+	+	+
Sikkenga, 2016	+	+	+	-	2+	-	-	2+	2+	NR	-	2+	2+	2+	2+	2+	2+	2+	-	NR	NR	NR	2+	2+	-	+
Lee, 1996	+	+	+	-	2+	-	-	2+	2+	NR	-	2+	2+	2+	2+	2+	2+	2+	-	NR	NR	-	2+	2+	-	+

For peer review only

Dixon, 2014	+	2+	+	2+	2+	+	-	2+	2+	NR	2+	2+	2+	2+	2+	2+	2+	2+	2+	-	2+	NR	2+	2+	-	+
Lee, 2014	+	2+	+	+	2+	NR	+	2+	2+	NR	2+	2+	+	2+	2+	2+	2+	2+	2+	NR	2+	+	2+	+	+	+
Adam, 2014	+	2+	+	+	2+	2+	+	2+	2+	NR	2+	2+	2+	2+	2+	2+	2+	2+	2+	NR	2+	+	2+	2+	+	+
Fontanel, 2011	+	2+	+	-	2+	-	-	2+	2+	NR	-	NR	2+	2+	2+	2+	2+	2+	+	NR	NR	NR	NR	2+	-	+
Gaucher, 2010	+	2+	+	-	2+	-	-	2+	2+	NR	-	2+	2+	2+	2+	2+	2+	2+	-	NR	2+	2+	2+	2+	-	+
Demarco, 2010	+	2+	+	-	2+	-	-	2+	2+	NR	-	2+	2+	2+	2+	2+	2+	2+	-	NR	NR	-	2+	+	-	+
Jobe, 2013	+	2+	+	+	2+	NR	NR	2+	NR	NR	2+	2+	2+	+	2+	2+	2+	2+	2+	2+	NR	-	-	-	-	+
Lijuan, 2017	2+	2+	+	-	2+	-	-	2+	2+	NR	-	2+	+	2+	2+	2+	2+	2+	-	NR	NR	-	2+	+	-	+

For peer review only

Appendix Figure 1: Effectiveness of PHCP interventions on time to provide initial assessment (in minutes) sub-grouped by interventions.

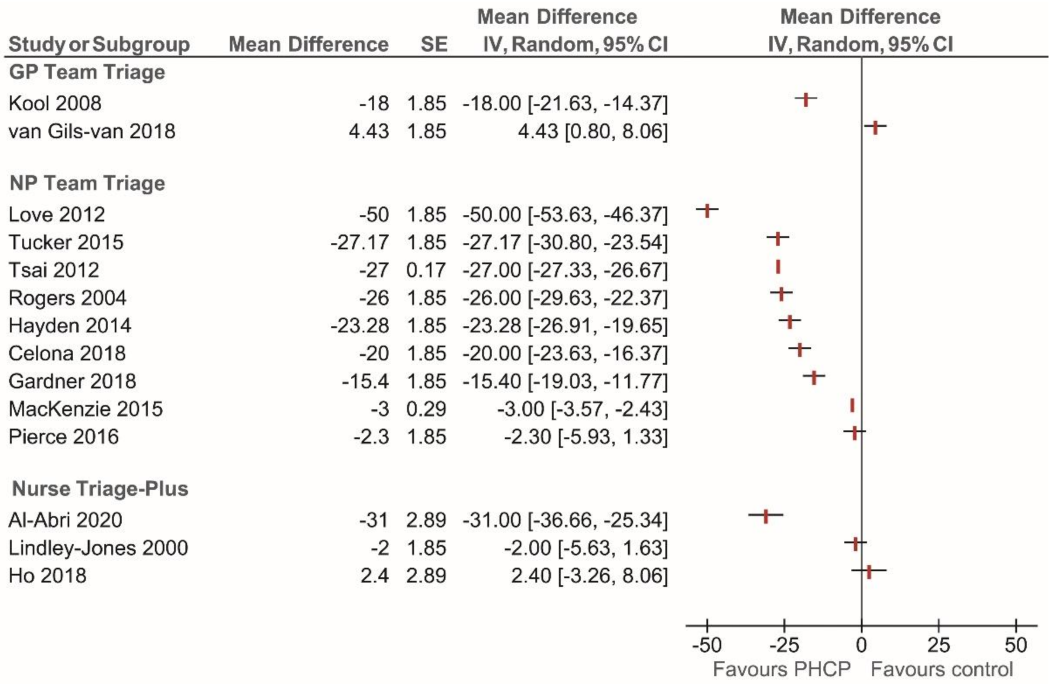

The horizontal black lines represent 95% confidence intervals and the red dots in the middle represents point estimates (mean difference).

review only

Appendix Figure 2: Effectiveness of PHCP interventions on achieving benchmark time to provider initial assessment

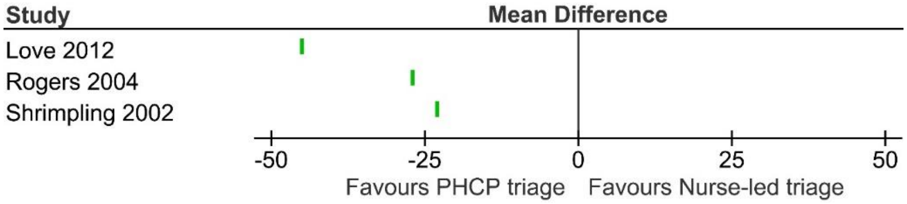

The horizontal black lines represent 95% confidence intervals and the red dots in the middle represents point estimates (mean difference).

For peer review only

Appendix Figure 3: Effectiveness of PHCP interventions on ED LOS (in minutes) sub-grouped by interventions.

Study or Subgroup	Mean Difference	SE	Mean Difference IV, Random, 95% CI
**GP Team Triage**			
van Gils-van 2018	4.52	23.9	4.52 [-42.32, 51.36]
**NP Team Triage**			
Day 2013	-37	0.94	-37.00 [-38.84, -35.16]
Gardner 2018	-34	4.8	-34.00 [-43.41, -24.59]
Hayden 2014	-16.65	23.9	-16.65 [-63.49, 30.19]
MacKenzie 2015	-20	1.13	-20.00 [-22.21, -17.79]
Pierce 2016	-28.5	23.9	-28.50 [-75.34, 18.34]
Rogers 2004	-22	23.9	-22.00 [-68.84, 24.84]
Tsai 2012	-58	0.83	-58.00 [-59.63, -56.37]
Tucker 2015	-18.3	23.9	-18.30 [-65.14, 28.54]
Zager 2018	-102	23.9	-102.00 [-148.84, -55.16]
**Nurse Triage-Plus**			
Adam 2014	-11	5.2	-11.00 [-21.19, -0.81]
Al-Abri 2020	-97	23.9	-97.00 [-143.84, -50.16]
Ashurst 2014	-6.5	23.9	-6.50 [-53.34, 40.34]
Cheung 2002	-46	23.9	-46.00 [-92.84, 0.84]
Demarco 2010	-193	23.9	-193.00 [-239.84, -146.16]
Dixon 2014	-55	23.9	-55.00 [-101.84, -8.16]
Fan 2006	-6.7	7.22	-6.70 [-20.85, 7.45]
Fontanel 2011	-64	23.9	-64.00 [-110.84, -17.16]
Hackman 2015	-10	4.03	-10.00 [-17.90, -2.10]
Ho 2018	-13	4.8	-13.00 [-22.41, -3.59]
Klassen 1993	-18	6.3	-18.00 [-30.35, -5.65]
Lee 1996	-18.6	23.9	-18.60 [-65.44, 28.24]
Lee 2014	-18.59	3.19	-18.59 [-24.84, -12.34]
Lee 2016	-28	23.9	-28.00 [-74.84, 18.84]
Li 2018	-15	0.96	-15.00 [-16.88, -13.12]
Parris 1997	-6	23.9	-6.00 [-52.84, 40.84]
Thurston 1996	-15	2.3	-15.00 [-19.51, -10.49]

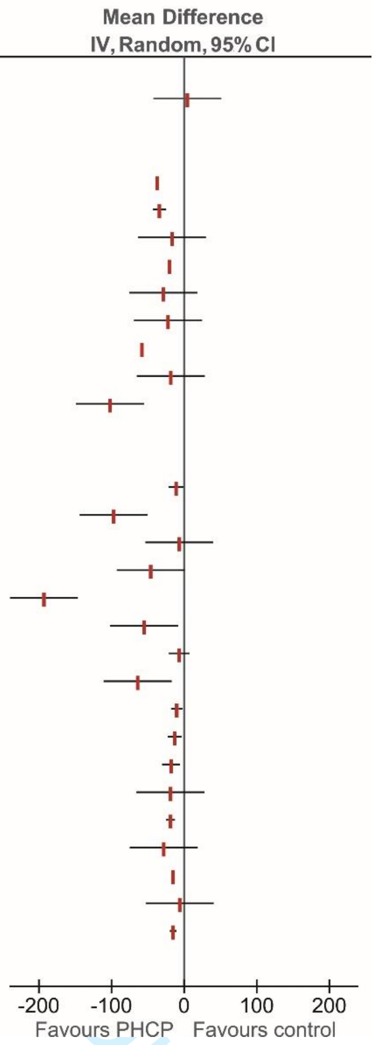

The horizontal black lines represent 95% confidence intervals and the red dots in the middle represents point estimates (mean difference).

References

1. Day TE, Al-Roubaie AR, Goldlust EJ. Decreased length of stay after addition of healthcare provider in emergency department triage: a comparison between computer-simulated and real-world interventions. *Emerg Med J.* 2013;30(2):134-138.
2. Hayden C, Burlingame P, Thompson H, Sabol VK. Improving patient flow in the emergency department by placing a family nurse practitioner in triage: a quality-improvement project. *Journal of Emergency Nursing.* 2014;40(4):346-351.
3. Lindley-Jones M, Finlayson BJ. Triage nurse requested x rays--are they worthwhile? *J Accid Emerg Med.* 2000;17(2):103-107.
4. Pierce BA, Gormley D. Are Split Flow and Provider in Triage Models in the Emergency Department Effective in Reducing Discharge Length of Stay? *Journal of Emergency Nursing.* 2016;42(6):487-491.
5. Tsai VW, Sharieff GQ, Kanegaye JT, Carlson LA, Harley J. Rapid medical assessment: improving pediatric emergency department time to provider, length of stay, and left without being seen rates. *Pediatric Emergency Care.* 2012;28(4):354-356.
6. Zager K, Taylor YJ. Discharge to medical home: A new care delivery model to treat non-urgent cases in a rural emergency department. *Healthcare.* 2018;21:21.
7. Lee WW, Filiatrault L, Abu-Laban RB, Rashidi A, Yau L, Liu N. Effect of Triage Nurse Initiated Radiography Using the Ottawa Ankle Rules on Emergency Department Length of Stay at a Tertiary Centre. *CJEM.* 2016;18(2):90-97.
8. Cheung WWH, Heeney L, Pound JL. An advance triage system. *Accident & Emergency Nursing.* 2002;10(1):10-16.
9. Love RA, Murphy JA, Lietz TE, Jordan KS. The effectiveness of a provider in triage in the emergency department: a quality improvement initiative to improve patient flow. *Advanced Emergency Nursing Journal.* 2012;34(1):65-74.
10. MacKenzie RS, Burmeister DB, Brown JA, et al. Implementation of a rapid assessment unit (intake team): impact on ED length of stay. *American Journal of Emergency Medicine.* 2015;33(2):291-293.
11. van Gils-van Rooij ESJ, Meijboom BR, Broekman SM, Yzermans CJ, de Bakker DH. Is patient flow more efficient in Urgent Care Collaborations? *European Journal of Emergency Medicine.* 2018;25(1):58-64.
12. Lijuan Z. Advanced Triage Protocols in the Emergency Department. *Advanced Triage Protocols in the Emergency Department.* 2017:1-1.
13. Adam H, Tamim H, Altamimi S, et al. Effect of triage nurse ordered distal extremity X-rays on emergency department length of stay: A randomized controlled trial. *Academic Emergency Medicine.* 2014;21(5 SUPPL. 1):S195.
14. Al Kadhi O, Manley K, Natarajan M, et al. A renal colic fast track pathway to improve waiting times and outcomes for patients presenting to the emergency department. *Open access emergency medicine : OAEM.* 2017;9:53-55.
15. Ashurst JV, Nappe T, Digiambattista S, et al. Effect of triage-based use of the Ottawa foot and ankle rules on the number of orders for radiographic imaging. *The Journal of the American Osteopathic Association.* 2014;114(12):890-897.
16. Demarco F, Gerardo CJ, Boardwine A, et al. Effect of a nurse rapid intake initiative on patient length of stay and satisfaction: A project IMPACT initiative. *Academic Emergency Medicine.* 2010;17(SUPPL. 1):S93-S94.

For peer review only

17. Dixon A, Clarkin C, Barrowman N, Correll R, Osmond MH, Plint AC. Reduction of radial-head subluxation in children by triage nurses in the emergency department: A cluster-randomized controlled trial. *CMAJ.* 2014;186(9):E317-E323.
18. Fan J, Woolfrey K. The effect of triage-applied Ottawa Ankle Rules on the length of stay in a Canadian urgent care department: a randomized controlled trial. *Academic emergency medicine : official journal of the Society for Academic Emergency Medicine.* 2006;13(2):153-157.
19. Fontanel A, Besson C, Gallegos C, Vallot C, Droal D. Can X-rays be prescribed by a nurse in an emergency department? *European Journal of Emergency Medicine.* 2011;18(5):310-311.
20. Gaucher N, Bailey B, Gravel J. Triage nurses' counselling influences return visits of children leaving the emergency department before being seen by a physician. *Academic Emergency Medicine.* 2010;17(SUPPL. 1):S118-S119.
21. Hackman JL, Roth ED, Gaddis ML, Gratton MC. The effect of a nurse-initiated chest pain protocol on disposition time: A retrospective review. *Annals of Emergency Medicine.* 2015;66(4 SUPPL. 1):S9-S10.
22. Jobé J, Vandercleyen C, Ghuysen A, D'Orio V. Prospective study of an advanced nurse triage for a target pathology at the admission in the emergency department. *Acta clinica belgica.* 2013;68(6):482-.
23. Klassen TP, Ropp LJ, Sutcliffe T, et al. A randomized, controlled trial of radiograph ordering for extremity trauma in a pediatric emergency department. *Annals of emergency medicine.* 1993;22(10):1524-1529.
24. Lee KM, Wong TW, Chan R, Lau CC, Fu YK, Fung KH. Accuracy and efficiency of X-ray requests initiated by triage nurses in an accident and emergency department. *Accident and emergency nursing.* 1996;4(4):179-181.
25. Lee WW, Filiatrault L, Abu-Laban RB, Rashidi A, Yau L, Liu N. Effect of triage nurse initiated radiography using the Ottawa Ankle Rules on emergency department length of stay at a tertiary care center. *Canadian Journal of Emergency Medicine.* 2014;16(SUPPL. 1):S38.
26. Sikkenga T, Dumkow L, Draper H, et al. Implementation of a nursing triage order to improve utilization of rapid diagnostic testing for chlamydia and gonorrhea in the emergency department. *Open Forum Infectious Diseases.* 2016;3(Supplement 1).
27. Al Abri FH, Muliira JK, Al Awaisi H. Effect of triage nurse-led application of the Ottawa Ankle Rules on number of radiographic tests and length of stay in selected emergency departments in Oman. *Japan journal of nursing science : JJNS.* 2020;17(1):e12270.
28. Ho JK-M, Chau JP-C, Chan JT-S, Yau CH-Y. Nurse-initiated radiographic-test protocol for ankle injuries: A randomized controlled trial. *International emergency nursing.* 2018;41:1-6.
29. Li Y, Lu Q, Du H, Zhang J, Zhang L. The Impact of Triage Nurse-ordered Diagnostic Studies on Pediatric Emergency Department Length of Stay. *Indian journal of pediatrics.* 2018;85(10):849-854.
30. Parris W, McCarthy S, Kelly AM, Richardson S. Do triage nurse-initiated X-rays for limb injuries reduce patient transit time? *Accid Emerg Nurs.* 1997;5(1):14-15.
31. Thurston J, Field S. Should accident and emergency nurses request radiographs? Results of a multicentre evaluation. *J Accid Emerg Med.* 1996;13(2):86-89.
32. Gardner RM, Friedman NA, Carlson M, Bradham TS, Barrett TW. Impact of revised triage to improve throughput in an ED with limited traditional fast track population. *American Journal of Emergency Medicine.* 2018;36(1):124-127.
33. Rogers T, Ross N, Spooner D. Evaluation of a 'see and treat' pilot study introduced to an emergency department. *Accident and Emergency Nursing.* 2004;12(1):24-27.
34. Tucker A, Bernard M. Making the Case for Nurse Practitioners in the Emergency Department: A Clinical Case Study. *Advanced Emergency Nursing Journal.* 2015;37(4):308-312.

For peer review only

35. Tsai VW, Sharieff GQ, Kanegaye JT, Carlson LA, Harley J. Rapid medical assessment: improving pediatric emergency department time to provider, length of stay, and left without being seen rates. *Pediatric emergency care.* 2012;28(4):354-356.
36. Uthman OA, Walker C, Lahiri S, et al. General practitioners providing non-urgent care in emergency department: a natural experiment. *BMJ Open.* 2018;8(5):e019736.
37. Kool RB, Homberg DJ, Kamphuis HC. Towards integration of general practitioner posts and accident and emergency departments: a case study of two integrated emergency posts in the Netherlands. *BMC Health Services Research.* 2008;8:225.
38. MacKenzie RS, Burmeister DB, Brown JA, et al. Implementation of a rapid assessment unit (intake team): impact on ED length of stay. *Am J Emerg Med.* 2015;33(2):291-293.

For peer review only

PRISMA 2009 Checklist

Section/topic	#	Checklist item	Reported on page #
**TITLE**			
Title	1	Identify the report as a systematic review, meta-analysis, or both.	1
**ABSTRACT**			
Structured summary	2	Provide a structured summary including, as applicable: background; objectives; data sources; study eligibility criteria, participants, and interventions; study appraisal and synthesis methods; results; limitations; conclusions and implications of key findings; systematic review registration number.	3
**INTRODUCTION**			
Rationale	3	Describe the rationale for the review in the context of what is already known.	5
Objectives	4	Provide an explicit statement of questions being addressed with reference to participants, interventions, comparisons, outcomes, and study design (PICOS).	7
**METHODS**			
Protocol and registration	5	Indicate if a review protocol exists, if and where it can be accessed (e.g., Web address), and, if available, provide registration information including registration number.	7
Eligibility criteria	6	Specify study characteristics (e.g., PICOS, length of follow-up) and report characteristics (e.g., years considered, language, publication status) used as criteria for eligibility, giving rationale.	7
Information sources	7	Describe all information sources (e.g., databases with dates of coverage, contact with study authors to identify additional studies) in the search and date last searched.	8
Search	8	Present full electronic search strategy for at least one database, including any limits used, such that it could be repeated.	8
Study selection	9	State the process for selecting studies (i.e., screening, eligibility, included in systematic review, and, if applicable, included in the meta-analysis).	8
Data collection process	10	Describe method of data extraction from reports (e.g., piloted forms, independently, in duplicate) and any processes for obtaining and confirming data from investigators.	8
Data items	11	List and define all variables for which data were sought (e.g., PICOS, funding sources) and any assumptions and simplifications made.	7
Risk of bias in individual studies	12	Describe methods used for assessing risk of bias of individual studies (including specification of whether this was done at the study or outcome level), and how this information is to be used in any data synthesis.	9
Summary measures	13	State the principal summary measures (e.g., risk ratio, difference in means).	9

For peer review only

PRISMA 2009 Checklist

Synthesis of results	14	Describe the methods of handling data and combining results of studies, if done, including measures of consistency (e.g., I^2) for each meta-analysis.	Pages 09-10

Section/topic	#	Checklist item	Reported on page #
Risk of bias across studies	15	Specify any assessment of risk of bias that may affect the cumulative evidence (e.g., publication bias, selective reporting within studies).	9
Additional analyses	16	Describe methods of additional analyses (e.g., sensitivity or subgroup analyses, meta-regression), if done, indicating which were pre-specified.	9
**RESULTS**			
Study selection	17	Give numbers of studies screened, assessed for eligibility, and included in the review, with reasons for exclusions at each stage, ideally with a flow diagram.	11
Study characteristics	18	For each study, present characteristics for which data were extracted (e.g., study size, PICOS, follow-up period) and provide the citations.	11
Risk of bias within studies	19	Present data on risk of bias of each study and, if available, any outcome level assessment (see item 12).	12
Results of individual studies	20	For all outcomes considered (benefits or harms), present, for each study: (a) simple summary data for each intervention group (b) effect estimates and confidence intervals, ideally with a forest plot.	13, 14
Synthesis of results	21	Present results of each meta-analysis done, including confidence intervals and measures of consistency.	13, 14, 15
Risk of bias across studies	22	Present results of any assessment of risk of bias across studies (see Item 15).	12
Additional analysis	23	Give results of additional analyses, if done (e.g., sensitivity or subgroup analyses, meta-regression [see Item 16]).	13, 14
**DISCUSSION**			
Summary of evidence	24	Summarize the main findings including the strength of evidence for each main outcome; consider their relevance to key groups (e.g., healthcare providers, users, and policy makers).	17
Limitations	25	Discuss limitations at study and outcome level (e.g., risk of bias), and at review-level (e.g., incomplete retrieval of identified research, reporting bias).	20
Conclusions	26	Provide a general interpretation of the results in the context of other evidence, and implications for future research.	21
**FUNDING**			
Funding	27	Describe sources of funding for the systematic review and other support (e.g., supply of data); role of funders for the systematic review.	23

From: Moher D, Liberati A, Tetzlaff J, Altman DG, The PRISMA Group (2009). Preferred Reporting Items for Systematic Reviews and Meta-Analyses: The PRISMA Statement. PLoS Med 6(7): e1000097.

For peer review only - http://bmjopen.bmj.com/site/about/guidelines.xhtml

PRISMA 2009 Checklist

doi:10.1371/journal.pmed1000097

For more information, visit: **www.prisma-statement.org**.

Page 2 of 2

For peer review only
